# PolyQ length-based molecular encoding of vocalization frequency in FOXP2

**DOI:** 10.1016/j.isci.2023.108036

**Published:** 2023-09-27

**Authors:** Serena Vaglietti, Veronica Villeri, Marco Dell’Oca, Chiara Marchetti, Federico Cesano, Francesca Rizzo, Dave Miller, Louis LaPierre, Ilaria Pelassa, Francisco J. Monje, Luca Colnaghi, Mirella Ghirardi, Ferdinando Fiumara

**Affiliations:** 1 *Rita Levi Montalcini* Department of Neuroscience, University of Turin, 10125 Turin, Italy; 2 Department of Chemistry, University of Turin, 10125 Turin, Italy; 3 *Jockey Club* College of Veterinary Medicine and Life Sciences, City University of Hong Kong, Kowloon Tong, Hong Kong SAR 518057, China; 4 Cascades Pika Watch, Oregon Zoo, Portland, OR 97221, USA; 5 Deptartment of Natural Science, Lower Columbia College, Longview, WA 98632, USA; 6 Department of Neurophysiology and Neuropharmacology, Medical University of Vienna, 1090 Vienna, Austria; 7 Division of Neuroscience, IRCCS *San Raffaele* Scientific Institute, 20132 Milan, Italy; 8 School of Medicine, Vita-Salute San Raffaele University, 20132 Milan, Italy

**Keywords:** Zoology, Molecular biology, Evolutionary biology

## Abstract

The transcription factor FOXP2, a regulator of vocalization- and speech/language-related phenotypes, contains two long polyQ repeats (Q_1_ and Q_2_) displaying marked, still enigmatic length variation across mammals. We found that the Q_1_/Q_2_ length ratio quantitatively encodes vocalization frequency ranges, from the infrasonic to the ultrasonic, displaying striking convergent evolution patterns. Thus, species emitting ultrasonic vocalizations converge with bats in having a low ratio, whereas species vocalizing in the low-frequency/infrasonic range converge with elephants and whales, which have higher ratios. Similar, taxon-specific patterns were observed for the FOXP2-related protein FOXP1. At the molecular level, we observed that the FOXP2 polyQ tracts form coiled coils, assembling into condensates and fibrils, and drive liquid-liquid phase separation (LLPS). By integrating evolutionary and molecular analyses, we found that polyQ length variation related to vocalization frequency impacts FOXP2 structure, LLPS, and transcriptional activity, thus defining a novel form of polyQ length-based molecular encoding of vocalization frequency.

## Highlights

• The lengths of two polyQ repeats (Q_1_ and Q_2_) in FOXP2 vary broadly across mammals
• The Q_1_/Q_2_ length ratio correlates quantitatively with vocalization frequency ranges
• The FOXP2 polyQ repeats form α-helical coiled coil structures and promote LLPS
• Evolutionary polyQ length variation regulates FOXP2 structure, LLPS, and function

## Graphical abstract

**Figure:**
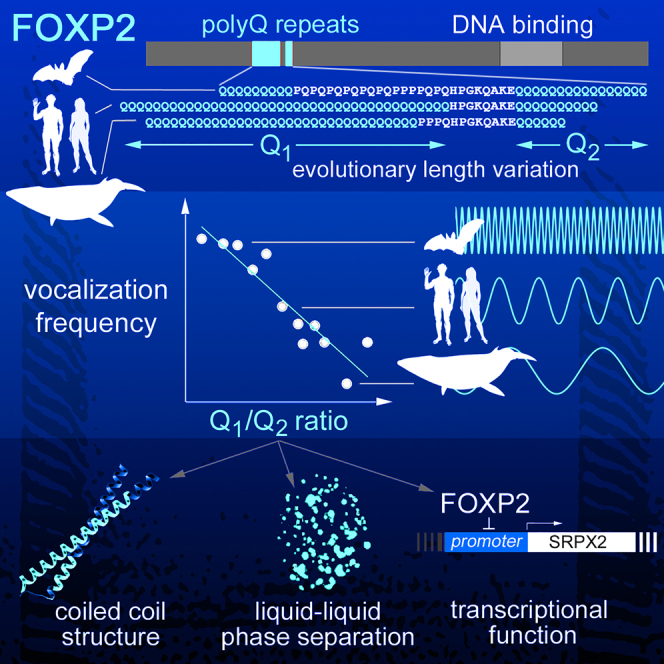

## Introduction

Vocalization is a biological process based on the emission of sound through the respiratory system underlying various forms of animal communication, e.g., human speech and oral language, intra-/interspecific signaling,^1^ as well as echolocation-based spatial navigation and prey hunting.^2^ Vocalizations can be emitted in the sonic frequency range, as defined by the human hearing sensitivity (∼20–20,000 Hz), and/or in the infrasonic (<20 Hz) or ultrasonic (>20 kHz) ranges.^2^^,^^3^ Vocalization and hearing frequency ranges generally co-evolve to allow efficient intraspecific communication.^4^^,^^5^ Thus, the ability of mammals to vocalize in frequency ranges inaccessible to other vertebrates arose also in relation with key evolutionary innovations in their hearing systems.^6^^,^^7^

The genetic and molecular bases of vocalization are only partially understood. In particular, it is unclear how specific features of vocalization, such as its frequency range in each species, are encoded at the genetic and molecular levels. Some genes related to vocalization, speech, language, and their evolution, have been identified^8^^,^^9^^,^^10^^,^^11^ and, among these, FOXP2 has been the most intensively studied.^12^^,^^13^

FOXP2 and its paralog gene FOXP1, both encoding for transcription factors, are involved in neural development and function,^13^ and are expressed in areas of the nervous system and sensory organs related to the emission and auditory processing of vocalizations (e.g., cerebral cortex, basal ganglia, auditory pathways, cochlea).^14^^,^^15^ These two transcription factors are also involved in the development of non-neural structures relevant to vocalization, such as the lung and the craniofacial skeleton.^16^^,^^17^^,^^18^

Mutations in both genes can give rise to various forms of speech and language impairments in humans.^19^^,^^20^

FOXP2 mutations can cause the ‘FOXP2-related speech and language disorder’ (FOXP2-SLD) with childhood apraxia of speech (CAS) as a core phenotype.^8^^,^^12^^,^^19^^,^^21^^,^^22^^,^^23^ FOXP2-SLD can also include oral-motor dyspraxia, dysarthria, moderate-to-severe receptive and expressive language disorder, reading impairments, and fine motor difficulties.^12^ Large 7q31 deletions or reciprocal-balanced translocations disrupting FOXP2 together with neighboring genes give rise to more complex phenotypes (‘FOXP2-plus-related disorder’).^12^^,^^19^ While hearing is not impaired in FOXP2-SLD, the possibility exists that higher-level auditory processing may be affected, as suggested by studies in genetically modified animal models.^14^^,^^24^

FOXP1 mutations can also lead to distinct speech and language phenotypes in the context of a broader neurodevelopmental ‘FOXP1-related disorder’, with prominent dysarthria and motor planning deficits, linguistic-based phonological errors, and generally more severe language and social impairments than those observed in the FOXP2-SLD.^20^

The molecular evolution of FOXP2 has also been associated with the emergence of specialized vocalization phenotypes. Indeed, two amino acid substitutions in human FOXP2, which are not present in the great apes, and intronic variants, have been related to the acquisition of speech and language.^25^^,^^26^^,^^27^ Furthermore, accelerated molecular evolution of FOXP2 has been detected in taxa with highly differentiated vocalization/hearing phenotypes related to echolocation, such as Chiroptera.^28^^,^^29^ Nevertheless, the specific impact of most of the observed evolutionary FOXP2 mutations on molecular and organismal phenotypes are still largely unclear.

Interestingly, both the FOXP2 and FOXP1 proteins contain two major polyglutamine (polyQ) repeats. One of these in FOXP2 represents the longest polyQ repeat in the human proteome.

PolyQ tracts can form α-helical coiled coils (CCs) that mediate protein oligo-/poly-merization and interactions,^30^^,^^31^ regulating the activity of transcription factors in a length-dependent manner.^32^^,^^33^ PolyQ CC regions, as CC domains in general,^34^ can drive the formation of functional molecular condensates through liquid-liquid phase separation (LLPS), as found for RUNX2^35^ which bears polyQ repeats forming CCs.^36^ However, the structural and functional roles of these long repeats in FOXP2 and FOXP1 are essentially unknown.

The evolutionary variation of polyQ and other amino acid repeats, in terms of occurrence and length, can have an evolutionary impact by adjusting, as ‘tuning knobs’, the transcriptional activity of developmental proteins.^32^^,^^36^^,^^37^^,^^38^^,^^39^^,^^40^ Thus, quantitative analyses of amino acid repeats, in terms of occurrence rate, length, and length ratios, can help characterize molecular evolution processes.^36^^,^^39^^,^^40^^,^^41^

PolyQ repeats are enriched in human proteins with speech- and language-related roles like FOXP2,^41^ suggesting a potential effect of polyQ length variation on vocalization-/speech-related molecular and organismal phenotypes, consistent with initial observations of FOXP2 polyQ length variants in humans and other primates.^42^^,^^43^ However, the full range of evolutionary polyQ length variation in FOXP2, and its possible structural and functional impact, are not yet defined.

Vocalization and hearing frequency ranges varied extensively throughout phylogenesis, and similar specialized forms of vocalization/hearing developed in different branches of the mammalian evolutionary tree by convergent evolution. Thus, high- and low-frequency specialist species, vocalizing/hearing in the ultra- or infra-sonic ranges, respectively, are found in multiple orders including Primates, Rodentia, Chiroptera, Cetartiodactyla, and Afrosoricida.^44^^,^^45^^,^^46^^,^^47^

Convergent evolution of organismal phenotypes between distant species/taxa can arise from convergent changes at the molecular level. For instance, in chiropteran and cetacean species using ultrasonic echolocation, *prestin* and other hearing-related proteins display similar amino acid substitutions which regulate protein function.^48^^,^^49^^,^^50^^,^^51^ However, none of these convergent mutations is common to all high-frequency specialist species,^51^^,^^52^ as convergence at the organismal level may arise at the molecular level from multiple mutational pathways in partially overlapping sets of genes/proteins.

Limited knowledge exists on convergent molecular evolution in vocalization-related proteins. Lee et al.^53^ identified amino acid substitutions shared by echolocating Chiroptera and Cetacea in proteins expressed in fast-twitch muscles involved in the emission of high-frequency vocalizations. FOXP2 underwent accelerated molecular evolution in Chiroptera,^28^ suggesting a role for the protein in the evolution of echolocation, although no molecular convergence has been observed between Chiroptera and Cetacea in this protein. Even though FOXP2 polyQ repeats display considerable length variability across mammalian species, their possible convergent evolution in relation to vocalization frequency ranges remains entirely unexplored.

Thus, in search of possible genetic and molecular encodings of vocalization frequency, we focused here on the evolution of FOXP2 and its polyQ repeats in Mammalia, a taxon characterized at the same time by highly diversified forms of vocalization and by a considerable degree of polyQ length variation in FOXP2.

## Results

### FOXP2 polyQ lengths encode quantitatively ultrasonic vocalization frequency in Chiroptera

**Figure 1:**
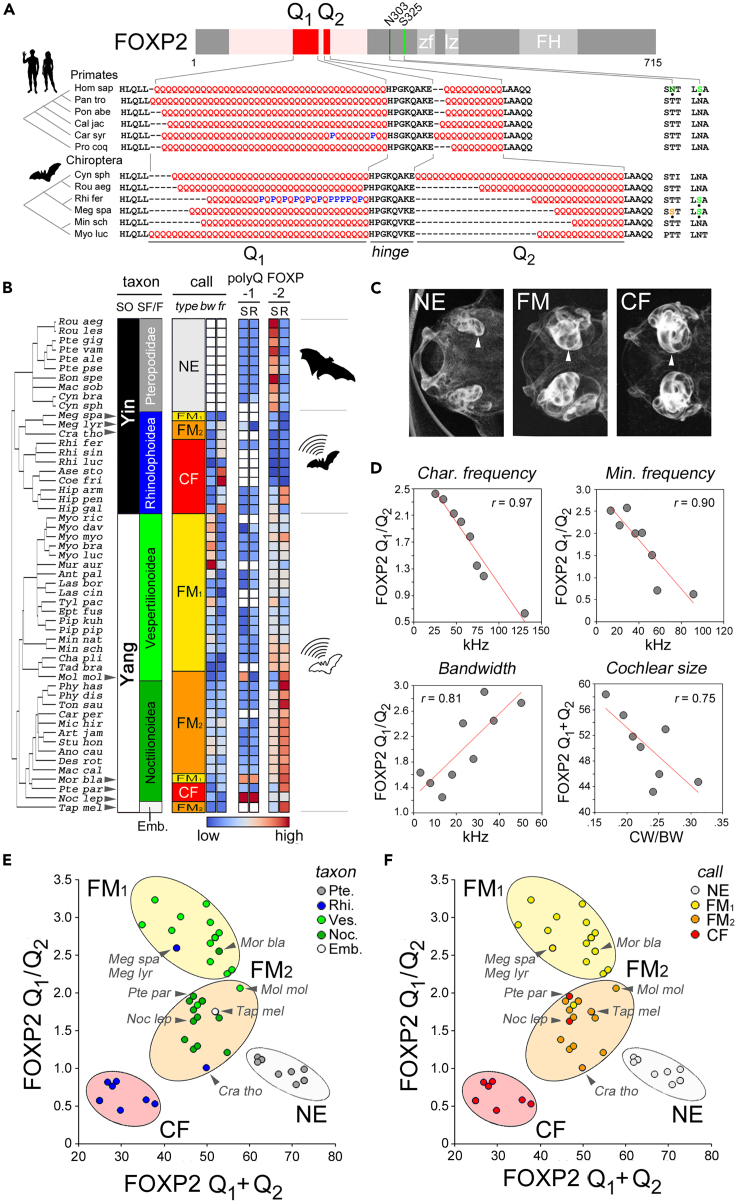
FOXP2 polyQ lengths encode ultrasonic vocalization frequency in Chiroptera (A) *Upper panel*: Scheme of the primary sequence of the human FOXP2 protein. The two major polyQ repeats (Q_1_ and Q_2_) are highlighted in *red* within a broader Q-rich N-terminal region in *pink*. Known domains are in *light gray*, i.e., *zf*: Zinc-finger; *lz*: leucine zipper; *FH*: Fork-head DNA binding domain. Residues N303 and S325, distinguishing the human ortholog among Primates,^25^ are in shades of *green*. *Lower panel*: partial sequence alignments of the polyQ regions (*left*) and of the region flanking the 303/325 positions of the human FOXP2 ortholog (*right*) in representative species of Primates and Chiroptera. Species names are abbreviated using the first three letters of genus and species, as reported in Table S1. On the *left*, unscaled phylogenetic trees (from TimeTree.org). Note how polyQ lengths vary also by proline residues insertion in Q_1_ and how certain bats bear human-like or other substitutions at positions corresponding to 303/325 in the human ortholog. (B) On the *left*, phylogenetic tree of the chiropteran species that were analyzed from TimeTree.org (species names in Table S1). On the *right*, the *taxon* bars specify for each species the suborder (SO; Yinpterochiroptera, YIN, and Yangochiroptera, YANG), and superfamily (SF) or family (F). The *call* panel indicates the call type (NE, FM_1_, FM_2_, CF, see results), bandwidth (*bw*) and characteristic frequency (*fr*), as reported in Collen.^55^ The *polyQ FOXP-1/-2* grids represent the total Q_1_ and Q_2_ length sum (*S*) and ratio (*R*) in FOXP1/2. *Bw*, *fr*, *S*, and *R* values are color-coded from *blue* (low) to *red* (high), as indicated *below*. Silhouettes represent NE megabats (*upper*) and USV-emitting microbats in Yinptero- (*middle*) and Yango-chiroptera (*lower*). *Black arrowheads* highlight species emitting USVs of a different type than the one prevalent in their superfamily. (C) Basicranium μ-CT stacks of representative species of the NE (*Pteropus alecto*), FM (*Macrotus californicus*), and CF (*Aselliscus stoliczkanus*) call type groups. Images are scaled to basicranium width to illustrate how the size of the cochlea (*white arrowheads*) relative to the basicranium increases progressively from NE to FM and CF.^56^ The scans were obtained from MorphoSource (ID: 0000S9128, 000036439, 000036171). (D) Scatterplots with regression lines (*red*) displaying significant correlations between mean (total or relative) FOXP2 polyQ lengths and mean USV frequency or cochlear parameters (i.e., characteristic and minimum frequencies, bandwidth, CW/BW) in groups of species binned by the same parameters (see STAR methods). (E and F) FOXP2 polyQ sum (Q_1_+Q_2_) versus ratio (Q_1_/Q_2_) plots for the available chiropteran sequences. Species are represented by *circles* colored based on phylogeny (taxa, *panel E*) or call type (NE, FM_1_, FM_2_, CF, *panel F*; species names abbreviations as in Table S1). The species group into four clusters (*ovals*) based on call type rather than phylogeny. The NE cluster is defined by a sum >60, while USV-emitting species clusters are defined by a sum <60 and increasing ratios for the CF (<1), FM_2_ (1–2.1) and FM_1_ (>2.1) groups. A*rrowheads* highlight the 8 species converging, in terms of call type, with species of other superfamilies. Six of them display a parallel convergence of polyQ lengths at the molecular level in FOXP2. *P. parnellii* (*Pte par*) and *N. leporinus* (*Noc lep*) are exceptions to this FOXP2 polyQ-based clustering but display, in comparison with their closest relatives, polyQ length changes in FOXP1 and, in *P. parnellii*, a unique Q-to-P substitution in the hinge region (see Figure S1E). See also Figure S1 and Tables S1, S2, S3, and S4.

To identify a possible link between FOXP2 polyQ repeat lengths and vocalization frequency, we initially focused on Chiroptera (∼20% of mammalian species)^54^ as a model taxon characterized by species with highly diversified ultrasonic vocalizations (USVs).^29^ Primary sequence analyses showed considerable length variation of FOXP2 polyQ repeats in Chiroptera in comparison with other taxa like Primates (Figure 1A). Thus, we reasoned that it would be easier in this taxon to reveal relations between polyQ variation and vocalization-related phenotypes.

Indeed, the two major N-terminal polyQ repeats, i.e., ‘Q_1_’ and ‘Q_2_’ (40 and 10 Qs in humans, respectively), display relatively limited variation in Primates. Conversely, Q_1_ and Q_2_ vary extensively across chiropteran species, in terms of both total (Q_1_+Q_2_ sum) and relative (Q_1_/Q_2_ ratio) lengths.

Notably, several species in both taxa exhibit a recurrence of interspersed proline residues and/or polyproline stretches in Q_1,_ which shorten this repeat compared to other related species.

The order Chiroptera comprises the Yinpterochiroptera and Yangochiroptera suborders (Figure 1B). ‘Microbats’ emitting laryngeal USVs, with constant frequency (CF) or frequency modulation (FM) and variable bandwidth/harmonics, are found in both suborders^55^ (Figure 1B).

For simplicity, we group FM USVs into ‘FM_1_’ (single harmonic, or with peak harmonic as the fundamental; Collen^55^ types 3, 7–8, 10), and ‘FM_2_’ (multi-harmonic; types 4–6, 9). Yangochiroptera also include ‘megabats’ that do not echolocate (‘NE’) using laryngeal USVs but navigate relying on sight and rudimentary echolocation, using sonic wing- or tongue-generated clicks.^57^

Notably, the use of these different USV types is not strictly determined by phylogeny in Chiroptera.^55^ Although specific USV types, such as CF in Rhinolophoidea, FM_1_ in Vespertilionoidea, and FM_2_ in Noctilionoidea, are prevalent in each chiropteran superfamily, several species demonstrate phenotypic convergence by employing USV types distinct from those of their closest relatives (as highlighted by arrowheads in Figure 1B). For example, *Megaderma spasma* (Rhinolophoidea) emits FM calls, *Pteronotus parnellii* (Noctilionoidea) CF calls, and *Mormoops blainvillei* (Noctilionoidea) uses FM_1_ calls. USV-emitting bats exhibit morpho-functional specializations, including an increased relative cochlear size^58^ (Figures 1C and S1A) and height (Figure S1B).

We measured Q_1_ and Q_2_ lengths in chiropteran FOXP2 orthologs, calculated their total (Q_1_+ Q_2_ sum) and relative (Q_1_/Q_2_ ratio) lengths, and studied their variation in relation to phylogeny and USV types (Figure 1B, *right columns*). This analysis corroborated the initial evidence of generalized polyQ variation in Chiroptera. A comparable analysis was conducted for two analogous polyQ repeats (‘Q_1_’ and ‘Q_2_’) in FOXP1, with length variation being restricted to only a few species (Figure S1C).

If the observed polyQ variation in FOXP2 is related to USV, we reasoned that polyQ length may correlate with vocalization-/hearing-related parameters. Thus, USV-emitting species were divided in groups based on USV frequency in 10 kHz bins. Then, we calculated the mean Q_1_+Q_2_ and Q_1_/Q_2_ for each group and correlated them with the respective mean USV frequencies. The source of USV frequencies was an extensive and standardized dataset provided by Collen^55^ which includes data for more than 300 bat species.

Notably, both the polyQ sum and ratio exhibit a strong correlation with the USV characteristic frequency (*r* = −0.97 for Q1/Q2, n = 8 groups, p < 0.0001; Figure 1D). Similar correlations are observed with minimum frequency and bandwidth (*r* = −0.90 and 0.81 for Q1/Q2, respectively, n = 8–9, p < 0.01 in both cases; Figure 1D). Additionally, a hearing-related morphological parameter, the relative cochlear size (calculated as the ratio between cochlear and basicranial widths, CW/BW, using data from Simmons et al.^58^) also shows significant correlations with polyQ repeat lengths. When species, including NE, are grouped based on cochlear size, the mean CW/BW ratio inversely correlates with the mean Q1+Q2 sum (*r* = −0.75, n = 8, p < 0.03; Figure 1D).

In summary, these findings indicate that the observed polyQ length variations in Chiroptera are closely related to vocalization- and hearing-related parameters, thus encoding USV frequency quantitatively.

### Convergent polyQ evolution in FOXP2 in relation to vocalization type in Chiroptera

The total and relative polyQ lengths exhibit differential variations among chiropteran species with different vocalization types (CF, FM, and NE; Figure 1B). This prompted us to investigate whether polyQ variation might be related not only to USV frequency but also to USV type. Therefore, we tested whether a combination of the two polyQ-related parameters could predict vocalization type by generating a scatterplot of polyQ sum vs*.* ratio (Figure 1E).

Remarkably, this graph demonstrates a clear clustering of the 53 species that were analyzed into four distinct groups based on their USV types (CF, FM_1_, FM_2_, NE) rather than their phylogenetic relationships (Figure 1F), with only two exceptions. The NE species are identified by a polyQ sum >60, while the USV-emitting species (sum <60) are further divided into three clusters based on their polyQ ratio (less than 1 for the CF group, 1–2.1 in the FM_2_ group, and from 2.1 to ∼3.5 in the FM_1_ group). The threshold values for these groups are highlighted in Figure S1D.

Indeed, it is worth noting how species belonging to the same Yinpterochiroptera suborder are differentially distributed among all four clusters based on their USV types. Specifically, Pteropodidae (NE) and Rhinolophoidea species utilizing CF USVs form two distinct clusters, well separated from those of FM species. Strikingly, the three Rhinolophoidea species emitting FM rather than CF USVs (*M. spasma*, *Megaderma lyra*, and *Craseonycteris thonglongyai*), cluster together with Yangochiroptera species that also emit FM calls. Similar cases of convergence can be observed across the FM_1_ and FM_2_ clusters. *Mormoops blainvillei* (Noctilionoidea) clusters with FM_1_ Vespertilionoidea species, while *Molossus molossus* (Vespertilionoidea) clusters with FM_2_ Noctilionoidea. *Taphozous melanopogon* (Emballonuroidea) groups with other FM_2_ species belonging to Noctilionoidea and Rhinolophoidea.

Two exceptions to the generalized clustering based on USV types are observed. First, *Pipistrellus pipistrellus*, an FM_1_ bat, shows convergence with FM_2_ bats. This convergence is of uncertain significance as it falls within the FM range and may be related to vocal plasticity in this species.^59^ The second exception is related to the CF species *Noctilio leporinus* and *Pteronotus parnellii* (Noctilionoidea) that do not converge with CF Rhinolophoidea. However, the lengths of FOXP1 polyQ repeats in both species vary considerably compared to most chiropteran species. Notably, this is also observed in other species that demonstrate USV type convergence with those from different orders (Figure S1C). Thus, it is possible that polyQ variation in FOXP1, rather than FOXP2, may be linked to changes in USV type in these particular species. Specifically, in these cases, the FOXP1 Q_1_/Q_2_ ratio is either below 0.5 (as in *P. parnellii*) or is ≥2 (as in *N. leporinus*) due to prevalent Q_2_ or Q_1_ elongation, respectively (Figure S1C). Moreover, *Pteronotus parnellii* also exhibits a unique proline substitution within the highly conserved FOXP2 hinge between Q_1_ and Q_2_ (Figure S1E), further distinguishing it from Noctilionoidea (see discussion).

Overall, these findings show how FOXP2 polyQ lengths can largely predict also USV type in Chiroptera. Moreover, they highlight the parallelism between phenotypic USV type convergence and molecular-level polyQ length convergence in FOXP2, with a few exceptions where enhanced polyQ variation is observed in FOXP1.

### Convergent polyQ evolution related to high-frequency vocalization/hearing in Euarchontoglires

**Figure 2:**
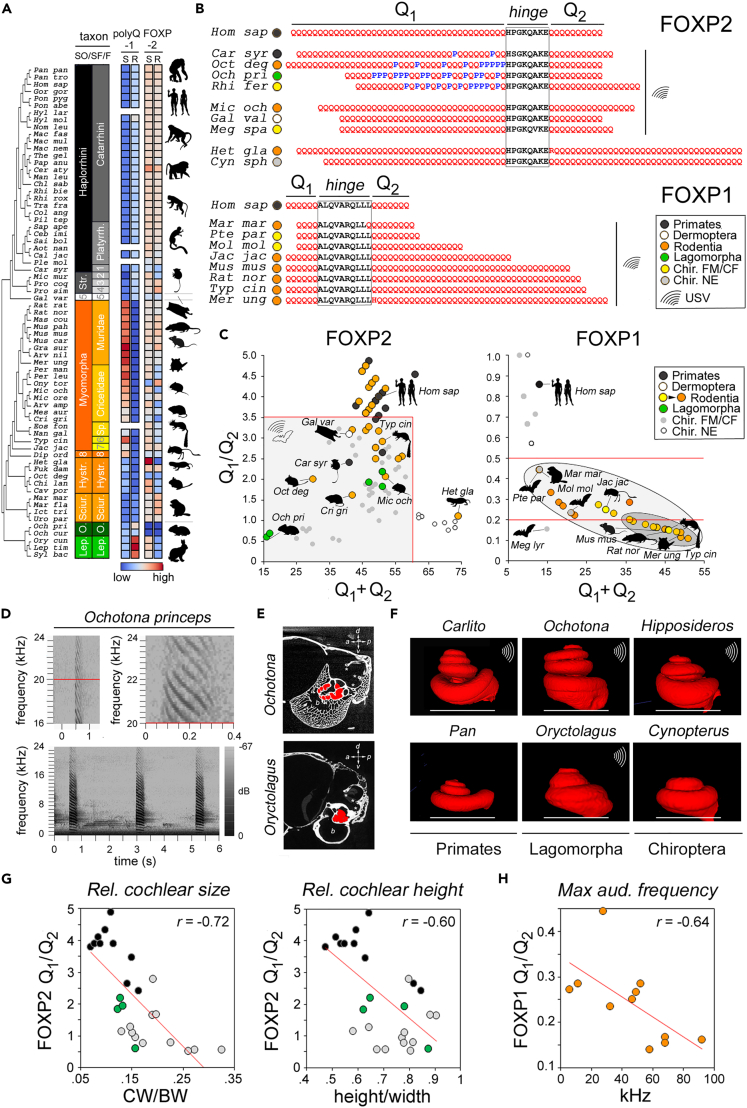
FOXP2 and FOXP1 polyQ lengths correlate with vocalization/hearing-related parameters in Euarchontoglires (A) As in Figure 1B, but for the superorder Euarchontoglires (species names as in Table S1). *SO* in the *taxon* bars indicates here ‘suborder’. Silhouettes of representative species are reported on the *right*. (B) As in Figure 1A, partial alignments of the polyQ repeat regions of FOXP2 (*upper*) and FOXP1 (*lower*) of the indicated species of Euarchontoglires and representative chiropteran species. Taxa and USV-emitting species are highlighted by symbols according to the legend in the inset. Note, for FOXP2, the polyQ convergence with USV-emitting bats (*Rhi fer*, *Meg spa*) of multiple Euarchontoglires species known to have ultrasonic vocalization/hearing, through Q_1_ contraction (also by proline insertions) and/or Q_2_ elongation in comparison with *Homo sapiens* (*Hom sap*). The fossorial rodent *Heterocephalus glaber* (*Het gla*), which has degenerate high-frequency hearing, converges instead with NE chiroptera (*Cyn sph*). Note, for FOXP1, the Q_2_ repeat expansion in USV-emitting Rodentia in comparison with other Euarchontoglires, exemplified here by *Homo sapiens* (*Hom sap*). The FOXP1 Q_1_/Q_2_ ratio decreases in rodents by either Q_1_ contraction and/or Q_2_ elongation. The USV-emitting bats *P. parnellii* (*Pte par*) and *M. molossus* (*Mol mol*) converge with rodents in having a low Q_1_/Q_2_ ratio. (C) PolyQ sum-*vs*-ratio plots, as in Figure 1E, for FOXP2 (*left*) and FOXP1 (*right*). Taxa are color-coded according to the legend. In the FOXP2 scatterplot, the *gray square* delimited by a *red line* indicates the region occupied by USV-emitting Chiroptera (sum <60, ratio <3.5). Note how several USV-emitting species from Primates (e.g., *Carlito syrichta*, *Car syr*), Dermoptera (*Galeopterus variegatus*, *Gal var*), Rodentia (e.g., the echolocating mouse, *Typhlomis cinereus, Typ cin*), and Lagomorpha (e.g., *Ochotona princeps*, *Och pri*) fall in the same region of the graph. In the FOXP1 scatterplot, all rodents (*larger oval*) display a lower ratio below 0.5 (*upper red line*), and many (*smaller oval*) below 0.2 (*lower red line*). Note how the ratio of *Homo sapiens* (0.85) and of most chiropteran species is higher, while some of them, i.e., *Pteronotus parnellii*, *Megaderma lyra*, *Molossus molossus*) converge with Rodentia in having a ratio <0.5. The same species also have polyQ and/or hinge mutations in FOXP2 associated with their phenotypic convergence with chiropteran species emitting similar USV types. (D) Representative spectrograms of three spontaneous *Ochotona princeps* calls recorded in the wild (*lower panel*). The calls are multiharmonic and, as shown for one of them in detail in the *upper panels*, they extend in the ultrasonic range, well above 20 kHz (*red line*). (E) μCT cross-sectional images of the basicranial region of the pika *O. princeps* (Morphosource 000045544) and *Oryctolagus cuniculus* (MorphoSource 000047005). The sagittal sections go through the cochlea (spiral turns filled in *red*), the tympanic bulla (*b*) and the semicircular canals (*asterisks*). The crossed arrows indicate skull orientation (*a*: anterior; *p*: posterior; *d*: dorsal; *v*: ventral). (F) Digital endocasts of the left cochlea of species belonging to Primates (*Pan troglodytes* and *Carlito syrichta*), Lagomorpha (*Oryctolagus cuniculus* and *Ochotona princeps*) and Chiroptera (*Rousettus aegyptiacus* and *Hipposideros armiger*) from μCT scans (Morphosource IDs: 000021952, 000158814, 000047005, 000045544, 000057664, 000025943). The images are scaled to have the same cochlear width, as in Ekdale, (2013),^62^ highlighted by a *white line* in each image. A wave symbol (in *white*) marks species with known ultrasonic vocalization/hearing. Note how USV-emitting species in both Chiroptera and Primates display relatively higher cochleae than their non-USV-emitting counterparts. In Lagomorpha, *O. princeps* displays a considerable relative height of the cochlea. (G) Scatterplots displaying the correlation between FOXP2 Q_1_/Q_2_ ratio and relative cochlear size (CW/BW; *left panel*) or relative cochlear height (height/width; *right panel*) of species belonging to *Primates* (*black*), *Lagomorpha* (*green*) and *Chiroptera* (*gray*). See also Figure S2E. (H) Scatterplot displaying the correlation between the FOXP1 Q_1_/Q_2_ ratio and the maximum audible frequency in 11 species of Rodentia. See also Figure S2 and Tables S1, S2, S3, and S4.

We further tested whether our findings in Chiroptera can also be generalized to other mammalian taxa, starting with Euarchontoglires (∼50% of mammalian species),^54^ the superorder comprising Primates, Rodentia, and Lagomorpha, in which numerous species are able to emit USVs^46^^,^^60^^,^^61^ (Figures 2A–2C and S2).

FOXP2 ortholog sequence alignments revealed a striking recurrence of the polyQ variation patterns and proline insertions, as found in USV-emitting bats, also in Euarchontoglires species with known ultrasonic vocalization/hearing (Figures 2A, S2A, and S2B). In these species, Q_1_/Q_2_ is < 3.5 (and Q_1_+ Q_2_ < 60), as typical of USV-emitting bats (Figures 1E and 1F), while it is > 3.5 in *Homo sapiens* (Q_1_/Q_2_ = 4) and other species devoid of ultrasonic vocalization/hearing. We observed convergence with NE bats only in a single species, *H. glaber,* a subterranean rodent which lost sensitivity to ultrasound.^63^

In FOXP1, the human-like polyQ pattern with two relatively short repeats (Q_1_ = 6, Q_2_ = 7) of similar length (Q_1_/Q_2_ = 0.85) can be found in most Euarchontoglires species (Figures S2C and S2D). However, species belonging to Rodentia, an order with widespread ultrasonic vocalization/hearing, display a generalized lowering of FOXP1 Q_1_/Q_2_ below 0.5, as found in *P. parnellii* and some other USV-emitting bat species (Figure 2B).

Sum-*vs*-ratio scatterplots (Figure 2C) highlight these recurring patterns of polyQ convergence. In the FOXP2 scatterplot, multiple species with known high-frequency vocalization/hearing fall into the region occupied by USV-emitting bats (sum <60, ratio <3.5; Figure 2C, *red boundaries*).

In Primates, three USV-emitting species, i.e., *Carlito syrichta*, *Microcebus murinus*, and *Callithrix jacchus*, displaye polyQ convergence with USV-emitting bats. Notably, *C. syrichta*, the only Primate known to emit purely ultrasonic calls,^46^ has the lowest polyQ ratio. In Dermotpera, *Galeopterus variegatus*, whose ultrasonic vocalizations have been recently demonstrated,^64^ also displays a Q_1_/Q_2_ ratio below 3.5.

In Rodentia, several species have FOXP2 polyQ lengths in the range of USV-emitting bats, such as *Microtus ochrogaster*,^65^ *Octodon degus*,^66^ and *Cricetulus griseus*.^67^ Strikingly, these species include *Typhlomys cinereus,* a blind rodent recently discovered to rely on echolocation for spatial navigation^60^ and showing molecular convergence with bats also for the hearing-related *prestin* protein.^68^

Nevertheless, numerous USV-emitting rodents, including the mouse and the rat, display no convergence with bats. Remarkably, however, Rodentia show overall considerable variation of the FOXP1 polyQ repeats, converging with USV-emitting bats like *P. parnellii* and *M. lyra* in having a low FOXP1 Q_1_/Q_2_ (<0.5). This ratio goes below 0.2 upon extreme Q_2_ expansion, as evident in some species with the highest vocalization/hearing frequencies, such as the mouse, the rat and the echolocating species *T. cinereus*. Species like *Jaculus jaculus* and *Marmota marmota,* adapted to desert and/or fossorial life, which are sensitive to both low-frequency and ultrasonic acoustic signals with a bimodal frequency sensitivity,^69^^,^^70^ have an intermediate Q_1_/Q_2_ ratio (0.2–0.5; also see below Afrotheria; Figures S4C and S4D).

All the analyzed Lagomorpha species have FOXP2 repeats in the range of USV-emitting bats. Two leporids (*Oryctolagus cuniculus* and *Lepus europaeus*) also converge with some USV-emitting bats in having a FOXP1 Q_1_/Q_2_ ≥ 2 (Figure S2D). While our knowledge on Lagomorpha vocalization/hearing is fragmentary, our findings are consistent with *Oryctolagus* audiograms showing ultrasonic sensitivity^71^ and recent reports of USVs in *Ochotona daurica*.^61^

The pika *Ochotona princeps* (*Och pri*) is an extreme case of low polyQ sum and ratio, clustering with USV-emitting bats. Strikingly, *O. princeps* displays accelerated evolution in the hearing-related protein *prestin* similar to echolocating bats,^72^ strongly suggesting its ability to vocalize/hear in the ultrasonic range.

To support this hypothesis as a case study of the predictive power of FOXP2 polyQ lengths for vocalization/hearing phenotypes, we analyzed vocalization recordings of *O. princeps* in the wild and studied its inner ear. We found evidence of spontaneous calls extending into the ultrasonic range, well above 20 kHz (>25 kHz; Figure 2D; see STAR methods). Moreover, available μ-CT scans revealed, in comparison with other Lagomorpha, an even larger, higher cochlea, within a highly pneumatized temporal bone (Figure 2E), consistent with high-frequency hearing sensitivity.^73^

Overall, these findings indicate that the observed convergence patterns of FOXP2 and FOXP1 polyQ repeats in relation to vocalization/hearing frequency are not unique to Chiroptera and can be generalized to other orders within Euarchontoglires.

### PolyQ lengths correlate with hearing frequency-related parameters in Euarchontoglires

As for Chiroptera, we also searched for quantitative relations between polyQ lengths and vocalization/hearing-related parameters in major Euarchontoglires orders. Given the previous findings, we performed this analysis in Primates and Lagomorpha for FOXP2 and in Rodentia for FOXP1.

As standardized measurements of vocalization-/hearing-related parameters for many Primates and Lagomorpha species are not available, we directly measured morphological cochlear parameters related to hearing frequency in species of interest (Figures 2F, 2G, and S2E–S2G).

Thus, we determined the relative cochlear size (CW/BW) and height (height/width)^62^ in representative species or genera of Primates (n = 10), Lagomorpha (n = 4) and, for comparison, Chiroptera (n = 16) (Figure S2E). This analysis revealed that cochlear parameters in these taxa correlate with polyQ lengths in FOXP2 (Figure 2F). Indeed, the Q_1_/Q_2_ ratio inversely correlates with relative cochlear size and height (*r* = −0.72 and −0.60, respectively, n = 26, p < 0.001 in both instances; Figures 2G and S2F). Thus, species with a lower ratio have significantly larger and higher cochleae (Figure S2G).

In Rodentia, we analyzed the correlation between FOXP1 polyQ lengths and a functional hearing-related parameter, the maximum audible frequency, in a sample of species with both parameters available. We found again an inverse correlation by which species with a low ratio, like *M. musculus*, tend to have auditory sensitivity to higher frequencies (*r* = −0.64, n = 11, p < 0.04; Figure 2H).

Taken together, these findings show that polyQ length variation in FOXP2 and FOXP1 encodes vocalization/hearing frequency-related parameters also in non-chiropteran mammalian taxa.

### Convergent polyQ evolution related to low-frequency/infrasonic vocalization in mammals

**Figure 3:**
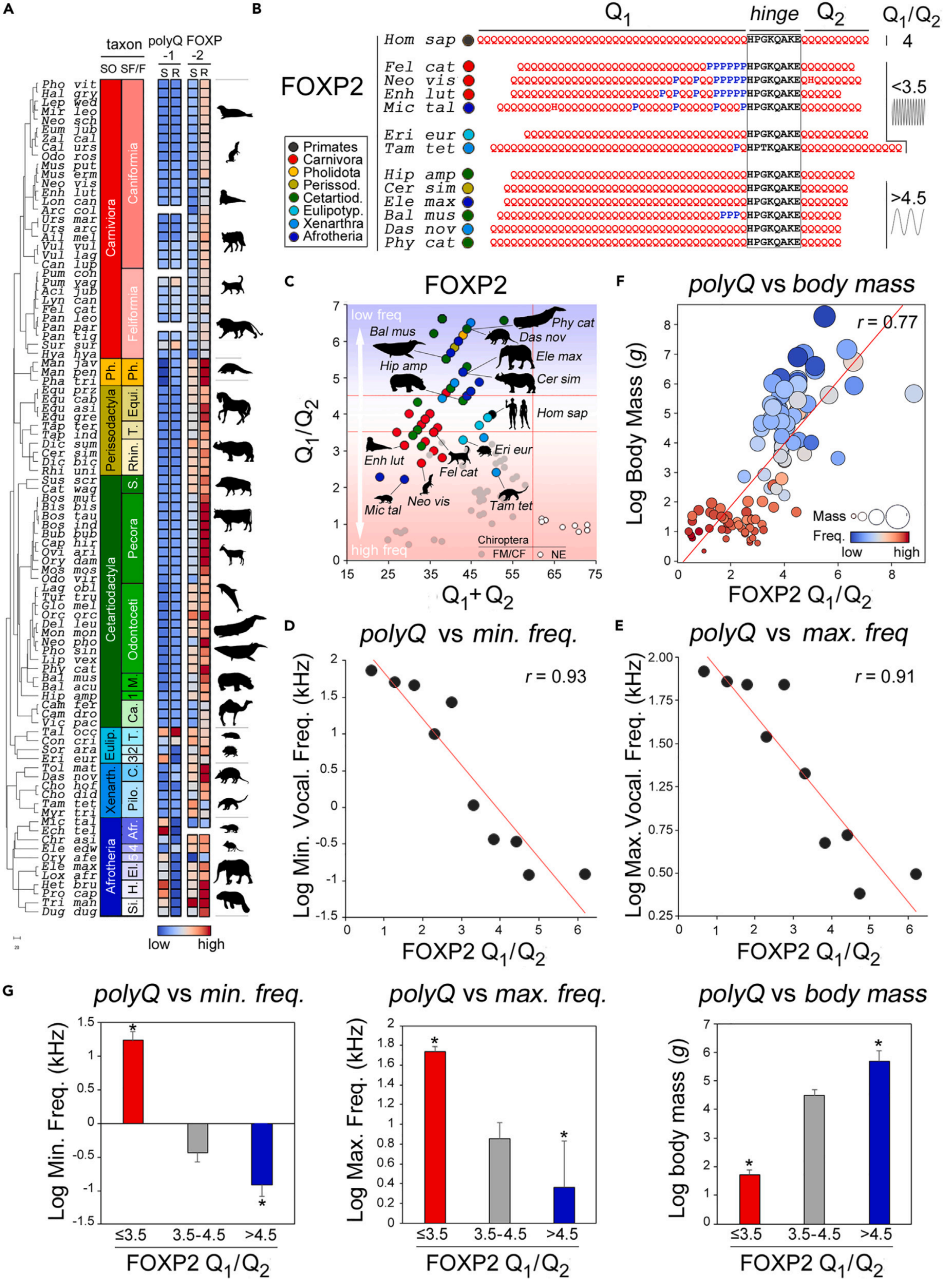
FOXP2 polyQ repeat lengths quantitatively encode vocalization frequency from the infrasonic to the ultrasonic range in Mammalia (A) As Figure 1B, for species belonging to non-chiropteran Laurasiatheria orders (i.e., *Carnivora* in *red*, *Pholidota* in *yellow*, *Perissodatyla* in *light green*, *Cetartiodactyla* in *dark green*, *Eulipotyphla* in *cerulean*), *Xenarthra* (*light blue*), and *Afrotheria* (*blue*). (B) As in Figure 1A, partial alignments of the FOXP2 polyQ regions for the indicated species belonging to phylogenetic tree shown in *panel A*. Taxa are highlighted by symbols according to the legend in the inset. The human sequence (Q_1_/Q_2_ = 4) is shown in comparison with species with known ultrasonic (Q_1_/Q_2_ < 3.5; *high-frequency sinusoid*) or infrasonic (Q_1_/Q_2_ < 4.5; *low-frequency sinusoid*) vocalization/hearing. The first group comprises some insectivorans (*Microgale talazaci*, *Mic tal*, Afrotheria; *Erinaceus europaeus*, *Eri eur*, Eulipotyphla)^45^ and laurasiatherian Carnivora (*Felis catus, Fel cat*^74^; *Neovison vison*, *Neo vis*^75^; *Enhydra lutris, Enh lut*).^76^ The second group comprises *Hippopotamus amphibius, Hip amp*, Cetartiodactyla; *Ceratotherium simum*, *Cer sim*, Perissodactyla; Elephas maximus, *Ele max*, Afrotheria; *Balaenoptera musculus*, *Bal mus*, Cetartiodactyla; *Physeter catodon*, *Phy cat*, Cetartiodactyla. See results for Xenartra (*Tamandua tetradactyla*, *Tam tet*; *Dasypus novemcinctus*, *Das nov*). The vertical red line marks the Q_1_+Q_2_ threshold of ∼60 beyond which only NE bats and *Heterocephalus glaber* are found. (C) Scatterplot as in Figure 2C, for the FOXP2 orthologs of species shown in *panel A*. The background *red* and *blue* gradients highlight the portions of the graph occupied by species with high- or low-frequency vocalization/hearing, respectively. Three species are omitted for clarity (*Manis javanica* and *M. pentadactyla*, both with Q1+Q2 = 44, Q1/Q2 = 10, and *Trichechus manatus, with* Q1+Q2 = 59 and Q1/Q2 = 8.83; see Figure S3). (D and E) Scatterplots with regression lines (in *red*) highlighting significant correlations between mean FOXP2 Q_1_/Q_2_ ratio and mean minimum (D) or maximum (E) vocalization frequency in groups of mammalian species binned by the same two parameters (see STAR methods). (F) Scatterplots with regression line (in *red*) display the correlation between FOXP2 Q_1_/Q_2_ ratio and log body mass (g) in 107 species of mammals with both parameters available. (G) Bar graphs plotting the mean log minimum (*left*) and maximum (*middle;* without Cetacea) vocalization frequencies, and the mean log body mass (*right*) in groups of species with a FOXP2 Q_1_/Q_2_ ≤ 3.5 (*red*), between 3.5 and 4.5 (*gray*), and >4.5 (*blue*). Data are represented as mean ± SEM, and the statistical tests that were performed are reported in the results section. *Asterisks* indicate statistically significant differences. See also Figures S3 and S4 and Tables S1, S2, and S3.

Thus, after Chiroptera and Euarchontoglires (∼70% of mammalian species),^54^ we completed our analyses with the remaining placental superorders, i.e., non-chiropteran Laurasiatheria (Carnivora, Pholidota, Perissodactyla, Cetartiodactyla, Eulipotyphla), Xenarthra, and Afrotheria (Figures 3A and S3; see Figure S3A for the few non-Eutherian species that could be analyzed). These taxa are of particular interest as they contain multiple species characterized by ultrasonic or infrasonic vocalization/hearing. For instance, insectivorans in Laurasiatheria (Eulipotyphla) and Afrotheria (Afrosoricida) independently developed ultrasonic signaling,^45^ while species with high body mass in multiple taxa, such as whales and hippos (Cetartiodactyla), elephants (Afrotheria), and rhinos (Perissodactyla) convergently evolved infrasonic vocalization/hearing.^47^^,^^77^

Strikingly, these two patterns of evolutionary phenotypic convergence toward either ultrasonic or infrasonic vocalization/hearing are paralleled, at the molecular level, by FOXP2 polyQ variations of opposite sign (Figures 3B and 3C).

Thus, species with known ultrasonic vocalization and/or hearing^45^^,^^74^^,^^75^^,^^76^ from Carnivora (e.g., *Felis catus*, *Neovison vison*, and *Enhydra lutris*), Eulipotyphla (e.g., *Erinaceus europaeus*) and Afrotheria (*Microgale talazaci*) have Q_1_/Q_2_ ≤ 3.5. (Figure 3B). Interestingly, also in these species Q_1_ shortening can arise from proline/polyP insertions. In sum-*vs*-ratio scatterplots these species fall in the region of USV-emitting Chiroptera and Euarchontoglires (Q_1_+Q_2_ ≤ 60; Q_1_/Q_2_ ≤ 3.5; Figure 3C).

Conversely, large species that evolved infrasonic vocalization/hearing^47^^,^^78^^,^^79^ such as *Balaenoptera musculus* (Mysticeti, Cetacea), *Elephas maximus* (Afrotheria), and *Ceratotherium simum* (Perissodactyla) all have Q_1_/Q_2_ > 4.5 (Figure 3B), occupying the upper part of the scatterplot above *Homo* (Q_1_/Q_2_ = 4) and other species with sonic vocalization/hearing ranges (Figure 3C). *Hippopotamus amphibius* (Cetartiodactyla) is a borderline case (Q_1_/Q_2_ = 4.38). Other large animals in Artiodactyla/Perissodactyla, also had Q_1_/Q_2_ > 4, except for some camelids, consistent with their ability to hear ultrasonic frequencies^80^ and emit the highest frequency vocalizations in Artiodactyla.^81^

Notably, Odontoceti cetaceans such as *Physeter catodon* and *Tursiops truncatus*, which use (ultra)sonic clicks for underwater echolocation, do not cluster with USV-emitting bats, but have FOXP2 polyQ repeats like infrasonic Mysticeti (ratio >4.5; Figure 3C; a few predicted sequences also bear a possibly spurious extension of the hinge peptide, see STAR methods). This finding parallels the lack of FOXP2 convergence between USV-emitting Chiroptera and Cetacea in terms of single residue substitutions,^28^ consistent with distinct vocalization systems. Indeed, while Chiroptera produce laryngeal vocalizations, Odontoceti emit non-laryngeal clicks through the forehead not requiring orofacial coordination, a process involving FOXP2^82^ (see discussion). The position of Odontoceti near Mysticeti in the scatterplot seems thus related to minimum, rather than maximum (USV-related) vocalization frequencies.^83^

Vocalization and hearing abilities are scarcely characterized in most Xenarthra species (Figure 3C). Interestingly, however, the fossorial *Dasypus novemcinctus*, thought to have low-frequency hearing,^84^^,^^85^ occupies the upper part of the scatterplot (ratio >4.5) while the insectivoran *Tamandua tetrapoda* is in the lower part, together with other non-xenarthran insectivorans.

In all these taxa, some species known to produce USVs also converge with some USV-emitting bats and most rodents in having a low FOXP1 Q_1_/Q_2_ ratio (<0.5; Figure S3C). These include only two insectivoran species from Eulipotyphla (i.e., *Erinaceus europaeus* and *Sorex Araneus*)^45^^,^^86^ and all of the species from Afrotheria. Thus, Afrotheria and Rodentia together constitute the vast majority of the species with a FOXP1 Q_1_/Q_2_ ratio <0.5.

Overall, these findings show that FOXP2 polyQ lengths vary and converge in opposite ways in mammalian species with high versus low frequency laryngeal vocalization/hearing, in the entire range from the infra- to the ultra-sonic. Interestingly, some USV-emitting species such as *M. lyra* (Chiroptera) and *T. cinereus* (Rodentia) show convergence for both FOXP2 and FOXP1.

### The FOXP2 Q_1_/Q_2_ ratio quantitatively encodes vocalization frequency ranges in Mammalia

The previous findings suggested that polyQ parameters may overall encode vocalization frequency ranges in mammals, and we tested this hypothesis for a sample of >100 species with available data.

We divided these species in ten groups based on increasing Q_1_/Q_2_ ratio (in 0.5 unit bins) and calculated for each group the mean minimum and maximum vocalization frequency as found in the literature (Figures 3D and 3E). We used in our correlation analyses the minimum/fundamental and maximum frequencies as overall indicators of the frequency range in each species, which can be very wide. These vocalization frequency parameters were derived from available datasets containing information for a large number of species (see Table S2 and STAR methods), rather than from a collection of studies in single species, to avoid data selection biases when discordant frequency values are reported by different sources. We found a strong significant correlation between mean Q_1_/Q_2_ and mean minimum (r = 0.93) or maximum (r = 0.91) vocalization frequency across groups (n = 10, p < 0.001 in both instances). Thus, a lower FOXP2 Q_1_/Q_2_ ratio is associated with a higher range of vocalization frequency and vice versa. Because acoustic allometry principles indicate that the vocalization frequency range is inversely related to body mass,^87^ we also tested whether the FOXP2 Q_1_/Q_2_ correlates with body mass in mammalian species (Figure 3F). We found again a strong correlation between Q_1_/Q_2_ and the logarithm of body mass (r = 0.77, n = 107, p < 0.001). Similar findings were obtained when Chiroptera were not included in the analysis (Figure S4A), thus highlighting how the overall relation between vocalization frequency and FOXP2 polyQ lengths is also present in non-chiropteran mammals.

When we compared quantitatively the mean vocalization frequencies (minimum and maximum) and body mass across species groups with low (≤3.5), intermediate (3.5–4.5), and high (>4.5) Q_1_/Q_2_ ratio, we found significant differences in all instances (ANOVA: min. freq.: F_(2,104)_ = 65.65; max. freq.: F_(2,80)_ = 26.25 and F_(2,70)_ = 30.05 without Cetacea; body mass: F_(2,104)_ = 85.22).

Strikingly, mammalian species with a lower FOXP2 Q_1_/Q_2_ ratio (≤3.5) have a significantly higher vocalization frequency (min. and max.) than species with intermediate, more human-like (3.5–4.5), or high (>4.5) ratios, as well as a lower body mass (Figure 3G; p < 0.001 in all comparisons, NK *post hoc* test). Conversely, species with a high ratio display significantly lower minimum frequency and higher body mass (p < 0.001–0.04 in all comparisons, NK *post hoc* test) in comparison with the other two groups. They also display lower maximum frequency than the low-ratio group (p < 0.001, NK test) and the intermediate group (Figures 3G and S4B). The latter difference reached statistical significance when Cetacea, which have high ratios and emit non-laryngeal USVs, are not included in the analysis (Figure 3G), consistent with the notion that minimum and maximum frequencies followed different evolutionary trajectories in this taxon.^88^

Notably, for Rodentia and Afrotheria, similar relationships exist between the FOXP1 Q_1_/Q_2_ ratio and vocalization frequencies as found for the FOXP2 ratio across mammals (Figures S4C and S4D).

Taken together, these findings indicate that the relative lengths of FOXP2 polyQ repeats predict overall frequency ranges in mammals, and strongly suggest the hypothesis that they may have some length-dependent structural and functional roles in the protein.

### The FOXP2 polyQ repeats form α-helical CCs whose extension and stability are regulated by their evolutionary length variation

The previous findings prompted us to define mechanistically the structural and functional impact of the observed evolutionary polyQ length variation in FOXP2. While the FOXP2 DNA-binding domain structure is solved,^89^ that of the polyQ-bearing N-terminus is unknown. Existing evidence strongly suggests that the FOXP2 polyQ repeats may form α-helical coiled coils (CCs) whose stability may be length-dependent and reduced by proline residues.^30^^,^^36^

**Figure 4:**
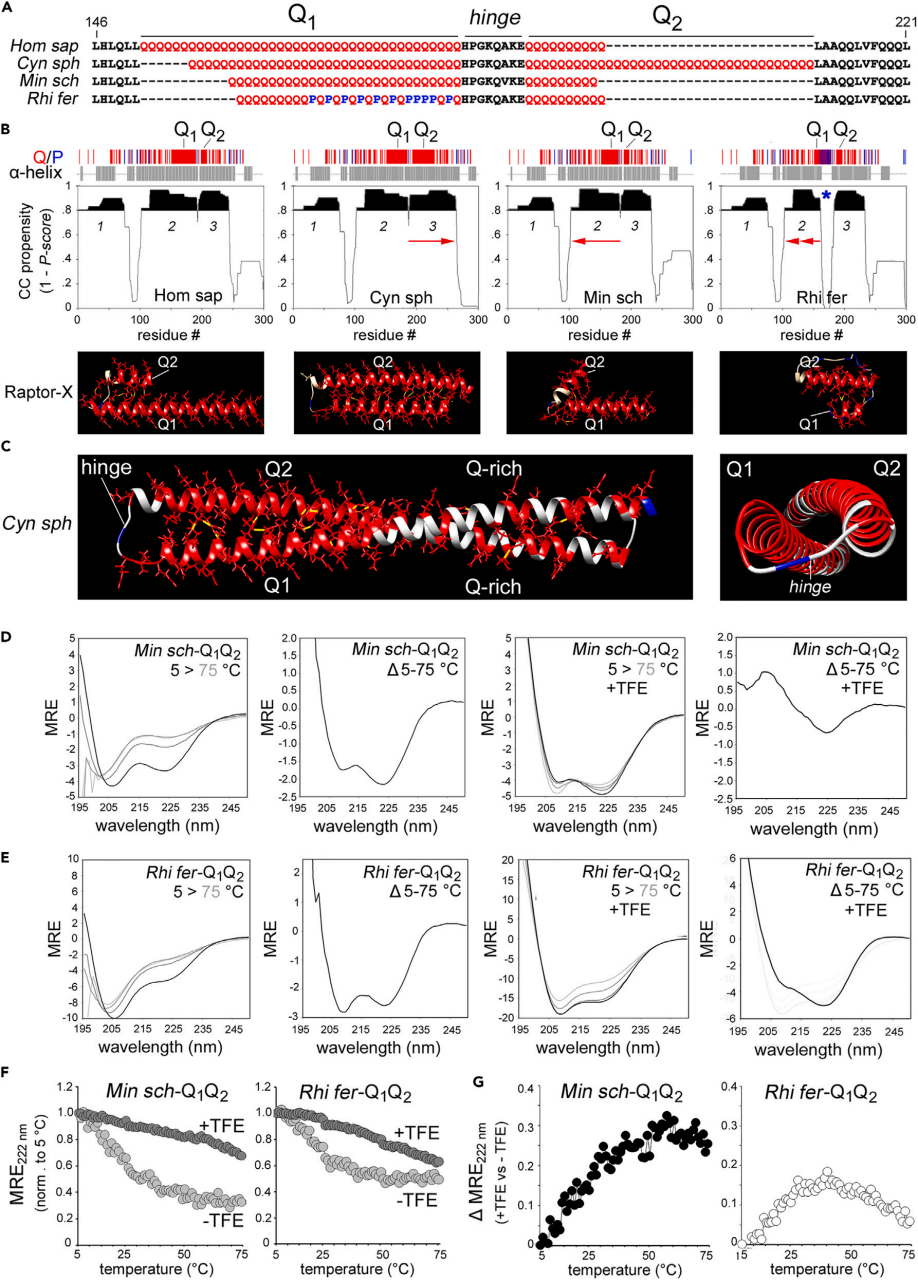
The FOXP2 polyQ region forms α-helical CCs with length-dependent stability (A) Primary sequence alignment of four peptides comprising the FOXP2 polyQ repeats, the hinge peptide, and a few flanking residues in the *Homo sapiens* (Hom sap), *Cynopterus sphinx* (Cyn sph), *Miniopterus schreibersii* (Min sch), and *Rhinolophus ferrumequinum* (Rhi fer) orthologs. Glutamine (Q) proline (P) residues are highlighted in *red* and *blue*, respectively, other residues are in *black*. (B) Secondary and supersecondary structure predictions for the Q-rich N-terminal domains (NTDs; first ∼300 residues) of the FOXP2 orthologs of the species listed above for *panel A*. On *top*, the thin vertical bars mark glutamine (*red*) and proline (*blue*) residues along the sequence. The *gray bars* highlight the regions predicted by PSI-PRED to form α-helical structures. The *middle* panels display the CC propensity calculated by Paircoil2^90^ and expressed as 1-*Pscore*, as in Fiumara et al.^30^ Regions with high CC-propensity (0.8–1) are highlighted in *black* (peaks *cc1*-*cc3*). *Red arrows* highlight, in comparison with *Hom sap*, *cc3* elongation related to Q_2_ expansion in *Cyn sph*, cc2 shortening related to Q_1_ contraction in *Min sch*, and further *cc2* shortening by Q_1_ interruption caused by numerous proline residues in *Rhi fer,* which greatly expand the gap between *cc2* and *cc3*. *Below*, details of Raptor-X atomic-level structural predictions highlighting the hairpin structure formed by Q_1_ and Q_2_ α-helices separated by a turn at the level of the hinge peptide. Glutamines are in *red*, prolines in *blue*, and Q-Q hydrogen bonds between the Q_1_ and Q_2_ are in *yellow*. Note how longer polyQ tracts correspond to longer α-helices and how the loop between the two helices is greatly extended by proline insertions in *Rhi fer*. (C) Lateral (*left panel*) and zenithal (*right panel*) views of Raptor-X structural model of the *Cyn sph* NTD region encompassing Q_1_, Q_2_ and the flanking Q-rich regions. Note how the supercoiled α-helical CC hairpin extends through the Q-rich regions. (D) CD spectra for *Min sch*-Q_1_Q_2_ peptide reporting the mean residue ellipticity (MRE) in the 190–250 nm interval. Overlay of spectra obtained at increasing temperatures (5, 25, 50, 75°C) and subtraction (Δ) of the spectra measured at 5°C and 75°C, either in saline buffer (two *left* panels) or in the same buffer with 80% v/v TFE (two *right* panels). (E) As in *panel D* for the *Rhi fer*-Q_1_Q_2_ peptide. (F) Plots of the 222 nm MRE over increasing temperatures (5°C–75°C) with (*dark gray*) or without TFE (*light gray*) for *Min sch*-Q_1_Q_2_ (*left panel*) and *Rhi fer*-Q_1_Q_2_ (*right panel*). MRE was normalized to its value at 5°C. (G) Graphs plotting the difference (Δ) in normalized MRE as a function of temperature, either in the presence or absence of TFE, as calculated from the graphs in *panel F*, for *Min sch*-Q_1_Q_2_ and *Rhi fer*-Q_1_Q_2_. See also Figure S5.

To determine the possible CC structure of the FOXP2 polyQ repeats and the impact of their variation, we first obtained structural predictions of the CC propensity of human FOXP2 and chiropteran orthologs with representative polyQ variation patterns (Figure 4A), i.e., expansion (*Cynopterus*), contraction (*Miniopterus*) and contraction by proline/polyP insertion (*Rhinolophus*).

Paircoil2 identified in human FOXP2 four high-probability CC regions, i.e., *cc1*-*4* (with (1 – P-score) ≥ 0.8)^30^ consistent with α-helical structure predictions (Figure 4B). *Cc2* and *cc3*, overlap with Q_1_ and Q_2_, respectively, whereas *cc4* is a known leucine zipper.^91^ While *cc1* and *cc4* are conserved across orthologs, the extension of *cc2* and *cc3* varies with polyQ length (Figures S5A–S5D), Proline residues in *Rhinolophus* widen the gap between *cc2* and *cc3*, normally caused by a single P within the hinge peptide. Atomic level structural predictions using Raptor-X^92^ (Figures 4C and S5E) are in agreement with Paircoil2 in showing Q_1_ and Q_2_, and flanking Q-rich regions, forming an antiparallel CC hairpin of two α-helices separated by a turn (hinge). The *Cynopterus* ortholog displayed the longest CC with Q-Q hydrogen bonds^93^ and the proline region in *Rhinolophus* forms a random coil loop between Q_1_ and Q_2_.

To experimentally test these predictions, we chemically synthesized Q_1_Q_2_ peptides (Figure 4A). Given the challenges in synthesizing/solubilizing long polyQ repeats,^94^ we started from two peptides with short (*Rhi fer*) and medium (*Min sch*) polyQ length. While the first displayed good solubility, *Min-sch-*Q_1_Q_2_ was already much less soluble, producing an opalescent solution indicating the prevalence of oligo-/poly-mers.^95^ Based on these observations, we did not further attempt to synthesize longer peptides, and we performed a comparative analysis of *Min-sch-*Q_1_Q_2_ *and Rhi-fer-*Q_1_Q_2_.

The CD spectra of *Min-sch-*Q_1_Q_2_ in saline buffer displayed α-helical structure with a random coil component (Figure 4D). The helical component was progressively disrupted by heating from 5°C to 75°C, with an isodichroic point at ∼203 nm, indicative of an equilibrium between α-helical and random coil conformations.^30^ The subtraction of 5°C and 75°C spectra shows that the structured component disrupted by heating has CC signatures, i.e., a ratio between the ellipticities at 222 and 208 nm > 1 (e.g., Fiumara et al.^30^; Figure 4D) indicating that the helical signal derives from a mixture of CC oligomers and random coil protomers rather than by partially helical protomers. Trifluoroethanol (TFE, 80% v/v) stabilized as expected the helical structure, even at 75°C, with a 222/208 nm ellipticity ratio >1, indicative of CC formation (Figure 4D), as also observed in the case of polyserine (polyS).^95^ A subtraction spectrum under these conditions (Figure 4D) had a single, red-shifted 222 nm peak and a blunted 208 nm peak indicative of CC polymers.^30^^,^^95^^,^^96^

Consistent with predictions, the *Rhi-fer-*Q_1_Q_2_ spectra revealed a lesser helical signal at 5°C, more easily destabilized by heating (Figure 4E). Subtraction spectra displayed a 222/208 nm ellipticity ratio <1, which may suggest the prevalence of α-helices over CC oligomers, although the random coil signal related to the predicted long, disordered proline-rich loop may also deepen the 208 nm peak, thus masking the CC signal (Figure 4E). This interpretation is corroborated by experiments in TFE in which subtraction spectra displayed a 222/208 nm ellipticity ratio >1 and a blunted 208 nm peak (Figure 4E), indicative of CCs in a polymeric state,^96^ as above.

The differential stability of the α-helical structures of the two peptides was also highlighted by the analysis of the absolute ellipticity at 222 nm upon heating (Figures 4F and 4G).

Altogether, these structural analyses show that the FOXP2 polyQ repeats and flanking peptides form α-helical structures with CC propensity, whose stability and oligomerization are modulated by polyQ length variation and proline insertion patterns as observed throughout phylogenesis.

### Atomic force microscopy (AFM) reveals condensates and fibrillary structures formed by FOXP2 polyQ peptides

**Figure 5:**
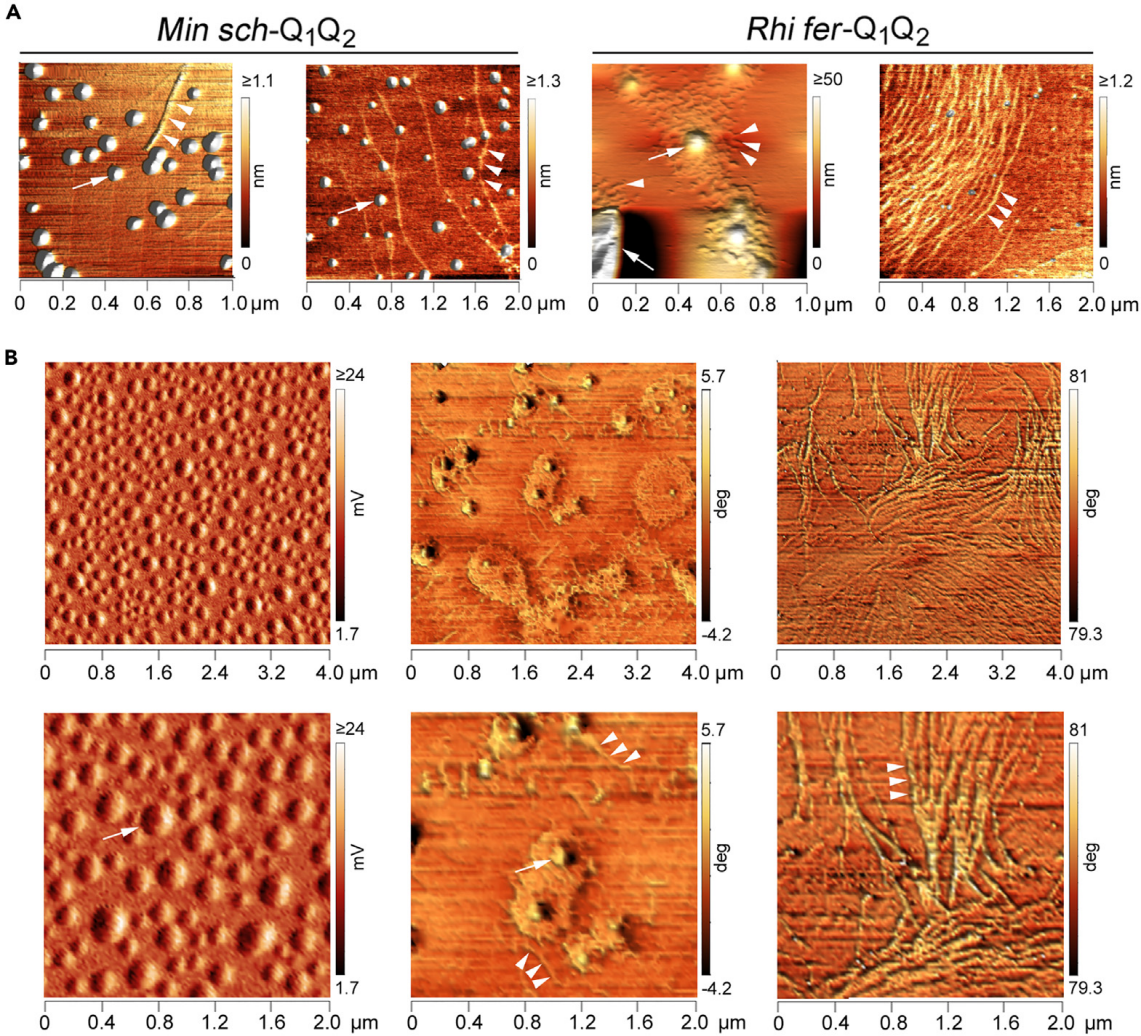
Morphological analysis of *Rhi fer*-Q_1_Q_2_ and *Min sch*-Q_1_Q_2_ by AFM (A) Zenithal 3-D renderings of AFM topography images of assemblies formed by *Min sch*-Q_1_Q_2_ and *Rhi fer*-Q_1_Q_2_ peptides in saline buffer (*first* and *third panel* from the left, respectively) or in the same buffer with 50% v/v TFE (*second* and *fourth panel*, respectively). *Arrows* indicate rounded condensates. *Arrowheads* mark fibrillary structures. Note, in the *third* panel, how *Rhi fer*-Q_1_Q_2_ forms transitional structures with a central condensate from which entangled fibrillary structures emerge radially. This happens also in the case of very large assemblies, such as the one indicated by the *arrow* in the lower left corner which is only partially visible in the scan. The signal related to condensates is deliberately saturated to allow the simultaneous visualization of thinner fibrillary structures. (B) Zenithal 3-D renderings of AFM amplitude or phase signals illustrating in detail the main three types of supramolecular assemblies formed by the *Min sch*-Q_1_Q_2_ and the *Rhi fer*-Q_1_Q_2_, as observed in *A,* after a more prolonged pre-incubation (see STAR methods). *Arrows* indicate rounded condensates, *arrowheads* mark fibrillary structures. The *lower panels* are details of the *upper images* (4 × 4 μm scan areas). The *left images* illustrate an area densely populated with rounded mesoscale condensates (tens-hundreds of nanometers) formed by *Min sch*-Q_1_Q_2_ in saline buffer. The two *middle images* illustrate transitional structures formed by *Rhi fer*-Q_1_Q_2_ in saline buffer. Note how a dense meshwork of fibrillary structures appears to emanate from condensate-like structures. The two *right images* illustrate an area occupied by multiple intertwining bundles of fibrils formed by *Rhi fer*-Q_1_Q_2_ in the presence of the helix-stabilizing agent TFE. Note how α-helix stabilization, as shown by the CD measurements, by TFE favors the elongation of fibrils in comparison with the *middle panels* (saline buffer). See also Figure S6.

To further define the organization of their oligo-/polymers at the morphological level, we studied the *Min sch*-Q_1_Q_2_ and *Rhi fer-*Q_1_Q_2_ peptides by means of AFM (Figure 5).

The AFM analysis of *Min sch*-Q_1_Q_2_, which forms an opalescent solution, was disturbed by numerous large peptide assemblies distributed unevenly on the mica surface, which made the scanning of smaller particles difficult, either in saline buffer or in the presence of TFE. Figure S6A exemplifies one of these, which may derive from tangling of fibrillary structures as previously found for polyA CC peptides.^36^ In areas free from large assemblies, we detected spherical droplets interspersed with thin fibrillar profiles (Figure 5A). The frequency of fibrillar profiles appeared to be increased by TFE (Figure 5A). The droplets ranged in diameter from a few nanometers to mesoscale condensates of tens-hundreds of nm (Figure 5B) which have been associated with LLPS.^97^

The more soluble *Rhi fer*-Q_1_Q_2_ peptide was easier to analyze. Interestingly, it formed complex structures with tangled fibrils emerging radially from a rounded central condensate, a transitional morphology between globular and fibrillary structures (Figure 5A). Notably, similar structures have been observed in AFM of proteins undergoing LLPS.^98^ The fibrillar component was enhanced by TFE, with the formation of large bundles of long fibrils (Figure 5A), consistent with the increase in the 222/208 nm ellipticity ratio in CD subtraction spectra measured in the presence of TFE (Figure 4E*, right panel*).

Together with the CD results, these findings indicate that CC α-helical peptides containing the FOXP2 polyQ repeats can assemble into LLPS-like mesoscale condensates and elongated fibrils, with transitional forms, as illustrated in Figure 5B. The relative proportion of these structures can be modulated by polyQ length, as indicated by the differential abundance of transitional structures formed by the two peptides. These results are consistent with evidence that CCs can drive the formation of both LLPS-driven condensates^34^^,^^35^ and fibrils,^30^^,^^96^^,^^99^ and that polyQ assemblies can transition from liquid-like condensates to more solid fibrillary structures.^100^

### Human FOXP2 forms nuclear LLPS-driven condensates

The previous results, and existing evidence that CCs and amino acid repeats can drive LLPS,^34^^,^^35^ prompted us to test whether FOXP2 can undergo LLPS in the cellular context.

**Figure 6:**
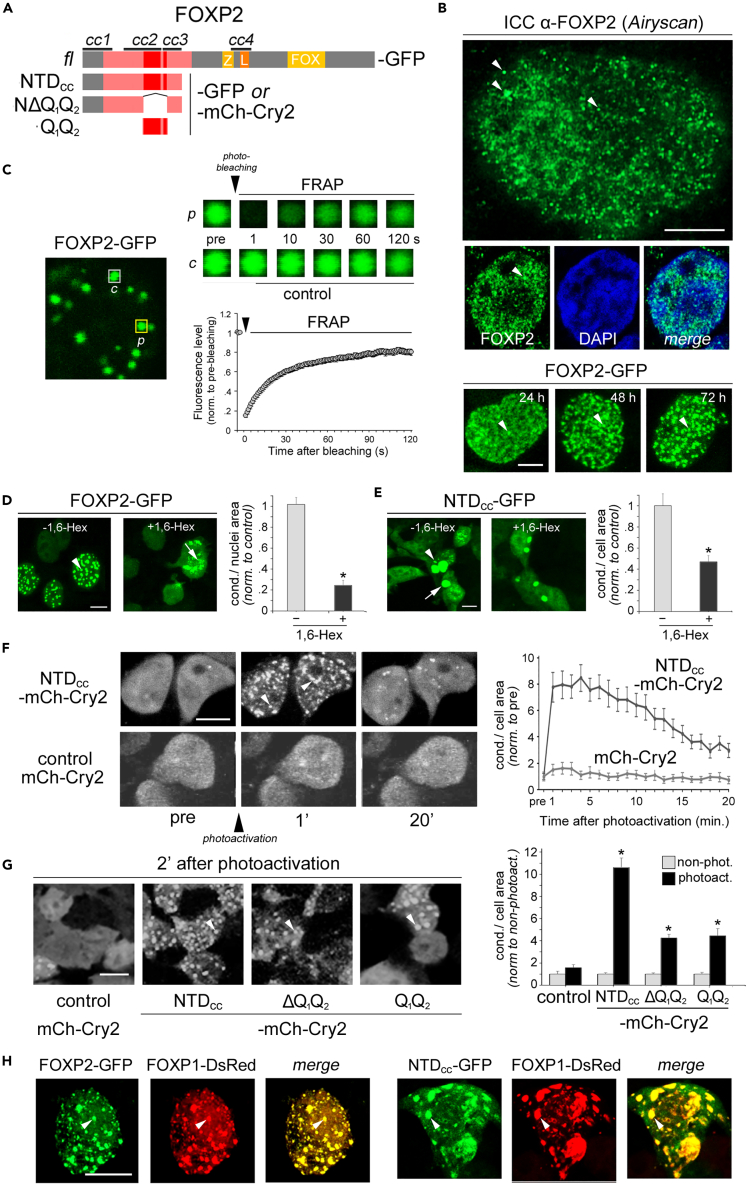
FOXP2 and its polyQ region undergo intracellular LLPS and recruit FOXP1 into condensates (A) Schematic representation of the constructs for the cellular expression of human FOXP2 protein or fragments of it, in fusion with either GFP or mCh-Cry2,^101^ that were expressed in the study of intracellular LLPS. Together with a construct for the expression of the full-length protein (*fl*, 715 a.a.) in fusion with GFP, we generated constructs for the expression of the NTD bearing three CC (*cc1*-*cc3*) regions (NTD_cc_, a. a. 1–244), the same NTD devoid of the polyQ repeats (ΔQ_1_Q_2_) and the polyQ fragment alone (Q_1_Q_2_), all in fusion with either GFP or mCh-Cry2. (B) *Upper panels*. *Airyscan* super-resolution confocal fluorescence images of nuclei of a Neuro-2a cell (*larger image*) and a HEK293 cell (*smaller images*, including DAPI staining in *blue*) after immunostaining of endogenous FOXP2. Note how the protein forms discrete foci (*arrowheads*) of variable size in the nucleus. A few foci in the cytoplasm may derive from a known, alternatively spliced isoform of FOXP2 (‘10+’) devoid of the C-terminus that localizes to the cytoplasm.^102^*Lower panels*. Confocal fluorescence images of cell nuclei of different HEK293 cells expressing FOXP2-GFP for 24 (*left*), 48 (*middle*), and 72 (*right*) hours. Note how the protein forms similar foci (*arrowheads*) as in the *upper panels* that become more defined with increasing time after transfection, as typical of LLPS-prone proteins.^103^ Calibration bars: 5 μm. (C) Molecular mobility within FOXP2-GFP foci as detected by FRAP. *Left panel*: confocal image of the nucleus of a FOXP2-GFP expressing cell in which target regions of interest (ROIs) are outlined by *yellow* lines. One of the ROIs was photobleached (*p*) while the other was used as a control (*c*). The *middle panel* shows confocal images of the two ROIs taken at the indicated timepoints before (*pre*) and after photobleaching. *Right panel*: FRAP curve of FOXP2-GFP. (D) *Left panel:* confocal fluorescence images of cells expressing FOXP2-GFP, either treated (+) or not (-; controls) with 1,6-Hex. The *arrowhead* indicates a condensate in control cells. The *arrow* indicates fragmented, residual condensates after 1,6-Hex application. Calibration bar: 10 μm. *Right panel*: bar graph representing the relative area occupied by condensates in nuclei of transfected cells in 1,6-Hex-treated (+) versus control (-) cultures. Data are represented as mean ± SEM, and the statistical tests that were performed are reported in the results section. *Asterisks* indicate statistically significant differences. The same applies to panels *E* and *G*. (E) As in *panel D*, but for the NTD_CC_-GFP construct. The *arrowhead* and *arrow* indicate, respectively, a small and a large condensate. Note how larger condensates are more resilient to 1,6-Hex. Calibration bar: 10 μm. (F) *Left panel*: time-lapse confocal images of HEK-293 cells expressing NTD_CC_-mCh-Cry2 (*upper panels*) or mCh-Cry2 (controls; *lower panel*) acquired before (*pre*) and after (1 and 20 min) photoactivation (*arrowhead*). *Right panel:* graph plotting the temporal kinetics of condensate formation and dissolution, quantified as the relative cell area occupied by condensates, up to 20 min after photoactivation in cells expressing either NTD-mCh-Cry2 (*dark gray*) or mCh-Cry2 (*light gray*). Calibration bar: 10 μm. (G) Confocal fluorescence microscopy images of cells expressing mCh-Cry2 alone (controls) or NTD_CC_, ΔQ_1_Q_2_, or Q_1_Q_2_, in fusion with mCh-Cry2, 2 min after photoactivation with 488 nm light (*left panel*). The bar graph (*right panel*) plots the relative cell area occupied by condensates in cell cultures expressing the four constructs that were either non-photoactivated (controls; *gray bars*) or photoactivated (*black bars*) with 488 nm light (values normalized to the non-photoactivated control for each group). Calibration bar: 10 μm. (H) *Left three panels*: confocal fluorescence microscopy images of the nucleus of a HEK293 cell co-expressing FOXP2-GFP and FOXP1-DsRed. Note in the overlay image (*merge*) the high degree of co-localization of the two proteins within the same condensates. (*see also*Figure S7D). *Right three panels*: as in the left panels but in cells co-expressing FOXP2 NTD_CC_-GFP and FOXP1-DsRed. Calibration bar: 10 μm. See also Figure S7.

Toward this aim, we first tested whether FOXP2 can form discrete foci in cells, and then whether these foci have recognized, distinctive properties of LLPS-driven condensates (Figure 6).

Using immunocytochemistry and super-resolution confocal microscopy (Airyscan) to reveal the subcellular distribution of FOXP2, we found that the endogenous protein forms numerous discrete foci of variable size in the nuclei of both HEK293 and Neuro-2a cells (Figure 6B*, upper panels*). These results were confirmed upon expression of GFP-tagged FOXP2, which formed similar foci that became larger and more defined in a time-dependent manner (Figure 6B*, lower panels*), as typical of proteins undergoing intracellular LLPS.^104^

Based on this morphological evidence, we further tested whether the observed FOXP2 nuclear foci exhibit typical features of LLPS-driven condensates.^35^^,^^103^ First, by performing fluorescence recovery after photo bleaching (FRAP) experiments (Figure 6C), we found that the FOXP2-GFP foci undergo rapid molecular exchange with substantial FRAP in 90–120 s (half-time 16.83 ± 1.92 s) and a mobile fraction of 78%, like other LLPS-prone proteins.^34^^,^^101^ Second, we found that the foci are sensitive to 1,6-hexanediol (1,6-HEX; Figure 6D), which disrupts the relatively weak molecular interactions underlying LLPS,^105^ as indicated by a ∽75% reduction of the relative condensate area in the nuclei of cells treated with 1,6-HEX for 15 min in comparison with the nuclei of control cells (0.24 ± 0.04, n = 24 fields, vs*.* 1 ± 0.06, n = 12, respectively, values normalized to control, p < 0.0001, *t*-test).

These findings indicate that FOXP2 can form LLPS-driven condensates in the cellular context.

### The polyQ-bearing N-terminal domain of FOXP2 drives LLPS and can recruit FOXP1

Our CD and AFM *in vitro* analyses strongly suggest that the polyQ repeats may drive FOXP2 LLPS. To test this hypothesis in the cellular context, we determined whether the isolated polyQ-bearing CC N-terminal domain (NTD_CC_) can recapitulate the LLPS behavior of FOXP2. When we expressed GFP-tagged NTD_CC_ in cells, we found that it forms rounded foci as full-length FOXP2. The foci were also present in the cytoplasm, consistent with the fact that the FOXP2 nuclear localization signal is in the C-terminal domain,^13^ and they were on average larger than those formed by FOXP2 (Figures 6E and S7A). These condensates displayed sensitivity to 1,6-HEX (normalized area: 0.47 ± 0.05, n = 24 microscopy fields, vs*.* 1 ± 0.11, n = 12, p < 0.001, *t*-test). Larger condensates were apparently less sensitive to 1,6-HEX, consistent with possible transitions to more solid-like configurations.^100^

To better define LLPS kinetics, we used the optoDroplet system, an optogenetic tool allowing to trigger the condensation of LLPS-prone protein domains in a spatiotemporally controlled manner.^101^ Thus, the NTD_CC_ was expressed in cells as a fusion protein with *mCherry-Cry2* (mCh-Cry2; Figures 6A and 6F), while mCh-Cry2 alone was expressed in control cultures. Live cell confocal imaging experiments in cells expressing these constructs revealed that NTD_CC_-mCh-Cry2 undergoes rapid LLPS upon 488 nm illumination, unlike the mCh-Cry2 control, which is reversible within 20–30 min (Figure 6F). Indeed, a two-way ANOVA revealed significant differences in the cell area occupied by condensates in relation to the expressed construct, time, and their interaction (F_(1,105)_ = 34.84, F_(20,2100)_ = 22.91, F_(20,2100)_ = 11.75, respectively, p < 0.001 in all instances). NTD_CC_-mCh-Cry2 condensation started within seconds after photoactivation and peaked after 2–4 min (7.94 ± 0.81 at 2 min vs*.* 1 ± 0.25 pre-activation, values normalized to the mean pre-activation relative condensate area, n = 58 cells, p < 0.001, *Newman-Keuls* (NK) *post hoc* test), whereas control cells expressing mCh-Cry2 did not display significant LLPS (p = 0.47 at 2 min vs*.* pre-activation, n = 49 cells). Moreover, LLPS was reversible, reinducible, and condensates displayed coalescence (Figures S7B and S7C), as typically found for LLPS-prone proteins in the cellular context.^34^

To determine the contribution of the polyQ repeats to the NTD_CC_-driven LLPS, we generated NTD_CC_ deletion constructs (Figure 6A) for the expression of the polyQ repeat region alone (Q_1_Q_2_-mCh-Cry2), or of the NTD_CC_ devoid of it (ΔQ_1_Q_2_-mCh-Cry2). We compared the relative capacity of these two fragments to induce LLPS with that of the whole NTD_CC_ by measuring condensate formation in cultures 2 min after photoactivation relative to untreated control cultures (Figure 6G). Overall, a two-way ANOVA revealed significant effects related to the expressed construct, photoactivation, and their interaction (F_(3,137)_ = 45.50, F_(1,137)_ = 180.66, F_(3,2137)_ = 45.50, respectively, p < 0.001 in all instances) in the induction of condensates upon photoactivation in comparison with basal levels.

Notably, LLPS induction was considerably lower for ΔQ_1_Q_2_-mCh-Cry2 in comparison with the NTD_CC_, showing that the polyQ repeats have an important role in driving FOXP2 LLPS (4.25 ± 0.33, vs*.* 10.61 ± 0.86 at 2 min, n = 20 fields, values normalized to the mean pre-activation relative condensate area in each group, p < 0.001, NK test). Indeed, the polyQ region alone (Q_1_Q_2_-mCh-Cry2) was still able to drive significant LLPS induction (4.46 ± 0.64 at 2′ post-activation vs*.* 1.00 ± 0.15 pre-activation, n = 10 in each group, values normalized to pre-activation levels, p < 0.001, NK test), although at lower levels in comparison with the whole NTD_CC_ (4.46 ± 0.64, n = 10, vs*.* 10.61 ± 0.86 at 2 min, n = 20, values normalized to pre-activation levels in each group, p < 0.001, NK test). Thus, also the Q-rich regions flanking the Q_1_Q_2_ repeats (Figure 1A) cooperate with them in driving LLPS.

As functionally related proteins can undergo LLPS and co-condensate within the same compartments,^35^ we tested whether this is the case for human FOXP2 and FOXP1. We found that the two proteins can co-condensate in cell nuclei and that the NTC_CC_ alone was able to mediate FOXP1 recruitment into condensates even in the cytoplasm (Figures 6H and S7D).

These findings identify the NTD_CC_ and its polyQ repeats as drivers of FOXP2 LLPS and highlight their role in the recruitment of interactors into condensates, consistent with the role of polyQ CCs in mediating homo-/hetero-typic protein interactions.^30^^,^^36^

### Evolutionary polyQ length variation regulates FOXP2 LLPS

**Figure 7:**
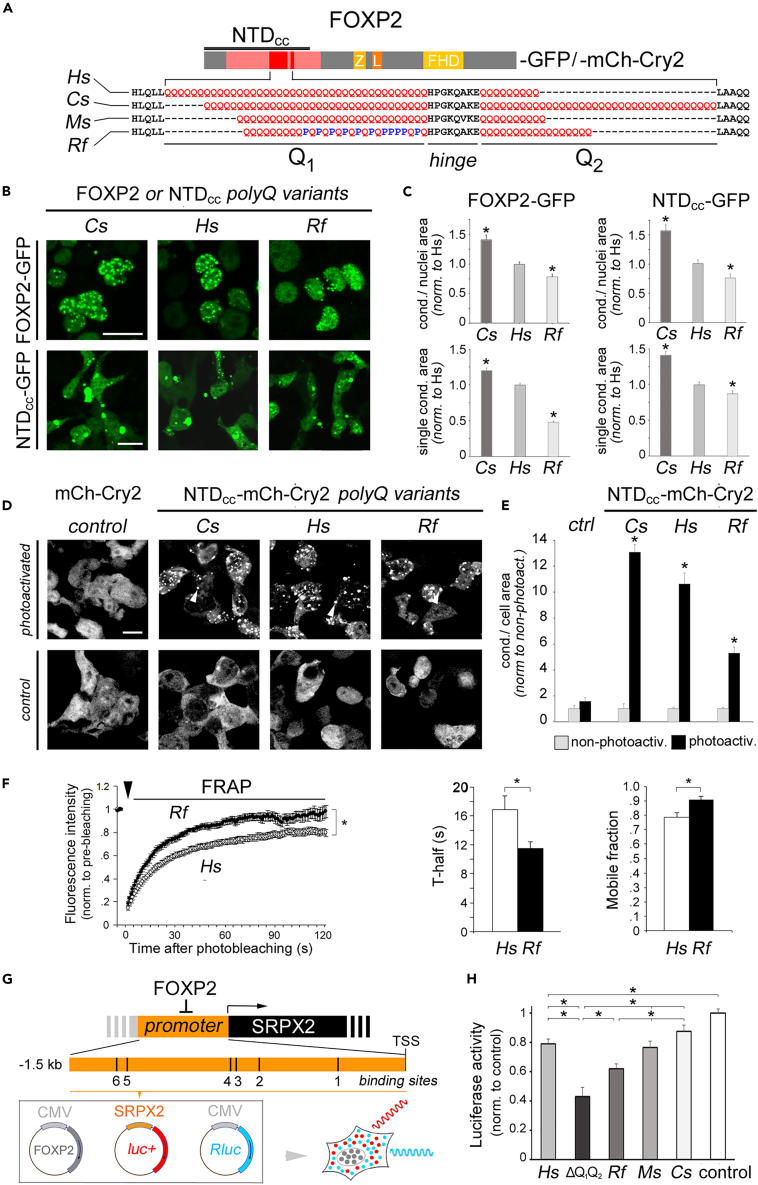
Evolutionary polyQ variation in FOXP2 regulates LLPS and transcriptional activity (A) Schematic representation of the human FOXP2 (*Hs*) polyQ length variants that were generated for functional analyses. To generate these variants, representative of the main evolutionary variation patterns, the endogenous polyQ repeats of the human ortholog were replaced with those of *Cynopterus sphinx* (*Cs*), *Miniopterus schreibersii* (*Ms*), and *Rhinolophus ferrumequinum* (*Rf*), whose aligned sequences are reported in *red*. These polyQ variants were cloned in constructs for their expression as GFP fusion proteins. Moreover, the NTD_CC_ domains of these isoforms were cloned into constructs for their expression as fusions with either GFP or mCh-Cry2. *Z*: zinc finger domain; *L*: leucine zipper; *FHD*: Forkhead domain. (B) Confocal fluorescence microscopy images of HEK293 cells expressing GFP-tagged human FOXP2 (*Hs*) or its polyQ variants (*Cs* and *Rf*), in either their full-length form (*upper panels*) or their NTD_CC_ fragments (*lower panels*). Calibration bars: 10 μm. (C) Bar graphs reporting the relative area occupied by condensates in nuclei (*upper graphs*) or the mean area of individual condensates (*lower graphs*) in cells expressing the polyQ variant constructs (*Hs*, *Cs*, and *Rf*), as in panel *B*, in either their full-length form (*left graphs*) or as NTD_CC_ fragments (*right graphs*). All values are normalized to mean values measured for the *Hs* variant. *Asterisks* indicate statistically significant differences in comparison with the *Hs* group. Data are represented as mean ± SEM, and the statistical tests that were performed are reported in the results section. *Asterisks* indicate statistically significant differences. The same applies to panels *E*, *F,* and *H*. (D) Confocal fluorescence microscopy images of HEK-293 cells expressing variants of the NTD_CC_ fragment of FOXP2 polyQ variants in fusion with mCh-Cry2 that either underwent photoactivation (*upper panels*) or were not-photoactivated (*controls*). Calibration bar: 10 μm. (E) Bar graphs reporting the relative area occupied by condensates in cells (*upper graphs*) as measured in photoactivated (*black bars*) or non-photoactivated (*gray bars*) cell cultures. Values are normalized to the mean value in the control, non-photoactivated cultures. *Asterisks* indicate, for each construct, a significant difference between the photoactivated and non-photoactivated cultures. (F) *Left panel:* FRAP curves of the GFP-tagged, full-length FOXP2 polyQ variants *Hs* and *Rf*. Values are reported, for each construct, as mean fluorescence intensity normalized to pre-bleaching values. *Middle and right panels:* bar graphs of the mean FRAP *t*-half (*middle*) and mean mobile fraction (*right*) for the polyQ variant constructs. (G) *Upper panel:* Scheme of the SRPX2 gene promoter (*orange bar*), which is repressed by FOXP2, highlighting multiple known binding sites (1–6).^106^*Lower panel*: Schematic representation of the plasmids co-transfected in luciferase assays for the expression of *i*) human FOXP2 or its polyQ variants controlled by the CMV promoter, *ii*) firefly luciferase (*luc+*) controlled by the SRPX2 promoter, and *iii*) *Renilla* luciferase (*Rluc*) controlled by the CMV promoter. (H) Bar graph displaying the relative *luc+* luminescence, normalized to a vector-only *control* experimental group (no FOXP2), measured in HEK293 cell cultures expressing human FOXP2 bearing either the human Q_1_ and Q_2_ repeats (*Hs*), or representative evolutionary variants (*Cs*, *Ms*, *Hs*), or no polyQ repeat (ΔQ_1_Q_2_). *Asterisks* indicate statistically significant differences between experimental groups connected by *horizontal lines*. See also Figure S7.

The previous evolutionary analyses strongly suggested that polyQ variation may regulate not only FOXP2 structure but also its function in terms of LLPS and transcriptional activity. To test this hypothesis, we generated constructs for the expression of chimeric variants of human FOXP2 (*Hs*) in which the polyQ repeats were either replaced with those of other species representative of the observed polyQ variation patterns, i.e., *Cynopterus sphinx* (*Cs*), *Miniopterus schreibersii* (*Ms*), or *Rhinolophus ferrumequinum* (*Rf*), or deleted (*ΔQ*_*1*_*Q2* variant). These variants were expressed as full-length proteins or as NTD_CC_ fragments, in fusion with either GFP or mCh-Cry2 (Figure 7A).

We first compared the ability to form condensates of the GFP-tagged human FOXP2 (*Hs*) with that of evolutionary variants with low (*Rf*) or high (*Cs*) polyQ length (Figure 7B). We found that the *Rf* and *Cs* condensates occupied, respectively, a lower and higher percentage of the transfected cell area in comparison with *Hs* condensates (F_(2,88)_ = 23.10, p < 0.001, one-way ANOVA; mean relative cell area occupied by condensates: 1 ± 0.04, n = 33 fields, for *Hs*; 1.41 ± 0.08 for *Cs*, n = 12; 0.78 ± 0.04 for *Rf*, n = 46; p < 0.02, for both the *Cs* vs*. Hs* and *Rf* vs*. Hs* comparisons; Figure 7C). The mean condensate size also varied similarly across constructs (Figure 7C; F_(2, 10321)_ = 222.34, p < 0.001). Similar results where obtained when only the NTD_CC_ domains of the three constructs were expressed (F_(2,86)_ = 25.19, p < 0.001, one-way ANOVA; mean relative cell area occupied by condensates: 1 ± 0.06, n = 32, for *Hs*, 1.55 ± 0.10 for *Cs*, n = 26, 0.75 ± 0.07 for *Rf*, n = 31, p < 0.03, for both the *Cs* vs*. Hs* and *Rf* vs*. Hs* comparisons, NK *post hoc* test; F_(2,4300)_ = 32.408, p <0 .0001, one-way ANOVA, for the mean condensate size). Consistent with these findings, the three NTD_CC_ variants displayed differential ability to undergo triggered LLPS (Figure 7D). Indeed, photoactivation induced a less extensive LLPS of *Rf*-NTD_CC_-mCh-Cry2 compared to *Hs-*NTD_CC_-mCh-Cry2, while the opposite was true for *Cs*-NTD_CC_-mCh-Cry2. Indeed, a two-way ANOVA (Figure 7E) revealed significant differences (p < 0.001) related to the construct (F_(3,158)_ = 75.62), photoactivation (F_(1,158)_ = 478.19), and their interaction (F_(3,158)_ = 75.62). Moreover, significant differences (p < 0.001; NK test) were found between all constructs and the control (mCh-Cry2), showing that all variants displayed significant LLPS induction upon photoactivation, and between *Hs* and either *Cs* or *Rf*.

Moreover, FRAP experiments further revealed that the mobility of molecules within condensates was higher for the *Rf* than for *Hs* variant (F_(1,45)_ = 23.932, p < 0.001, repeated measures ANOVA; Figure 7F), with a significant difference at 1′ post-bleaching between the two constructs (p < 0.03; NK test). Moreover, the *Rf* FRAP curve displayed, in comparison with the *Hs* one, a significantly lower time constant (11.55 ± 0.92, n = 17 condensates, vs*.* 16.83 ± 1.92, n = 26, p < 0.02, *t*-test) and a higher mobile fraction (90.64 ± 2.58%, n = 17 condensates, vs*.* 78.73 ± 3.19%, n = 26, respectively, p < 0.01, *t*-test).

Taken together, these findings indicate that the evolutionary polyQ variation in FOXP2 can modulate the mobility of the protein and its capacity to undergo LLPS.

### Evolutionary polyQ variation regulates FOXP2 transcriptional activity

Finally, we determined the possible regulatory effect of the evolutionary polyQ variation on the transcriptional activity of FOXP2. FOXP2 regulates several target genes, including SRPX2, another speech-/language-associated gene,^9^ whose expression is repressed by FOXP2. Indeed, FOXP2 has at least six binding sites in the SRPX2 promoter^106^ (Figure 7G).

Thus, we used luciferase reporter assays to measure the relative transcriptional activity of the FOXP2 polyQ variants (i.e., *Hs*, *Rf*, *Ms*, *Cs*, and *ΔQ*_*1*_*Q*_*2*_) as in Roll et al.^106^ HEK293 cells were co-transfected with a pcDNA4/HisMax expression vector encoding for any one of the FOXP2 polyQ variants, together with a second vector encoding for firefly luciferase (*luc+*) under the control of the SRPX2 promoter (SRPX2-pGL3-luc), and a third vector for the CMV promoter-driven expression of *Renilla* luciferase (*Rlu*c; pRL-CMV plasmid) as a transfection control. The empty pcDNA4/HisMax vector was transfected to measure basal luciferase levels in control cultures (Figure 7G).

Then, we compared the relative luciferase luminescence in cultures expressing FOXP2 polyQ variants. A one-way ANOVA indicated overall significant differences between the different polyQ variants (F_(5,338)_ = 29.10). *Post hoc* comparisons confirmed, as expected based on previous observations,^106^ that *Hs*-FOXP2 induces a significant reduction of *Luc*^*+*^ expression in comparison with control cultures, consistent with the repressive role of FOXP2 on SPRX2 (mean relative luminescence 0.79 ± 0.03, n = 69 culture wells, vs*.* 1 ± 0.02, n = 116, values normalized the mean control value, p < 0.001 NK *post hoc* test; Figure 7H).

Notably, *Rf* and *ΔQ*_*1*_*Q*_*2*_, with low or null polyQ length, displayed a significantly increased repressive activity in comparison with all other variants bearing longer polyQ tracts, including *Hs*-FOXP2 (p < 0.01 in all instances, NK test; Figure 7H). Overall, we observed a correlation between polyQ length and transcriptional repression (*r* = −0.97, n = 5, p < 0.01; Figure S7E). In species with predominant Q_1_ length variation (*Rf* and *Ms* vs*. Hs*), SRPX2 repression also correlated with the Q_1_/Q_2_ ratio (Figure S7F).

These findings indicate that the evolutionary polyQ variation may encode vocalization frequency at the molecular level by regulating quantitatively the transcriptional activity of FOXP2.

## Discussion

This study identifies a quantitative relationship between molecular and vocalization-related phenotypic parameters throughout mammalian evolution by which the FOXP2 Q_1_/Q_2_ length ratio encodes vocalization frequency, from the infrasonic to the ultrasonic ranges. Consistent with this finding, we observed generalized patterns of convergent polyQ length evolution related to vocalization/hearing frequency in FOXP2 and, for certain taxa, in FOXP1. At the molecular level, we found that the polyQ region of FOXP2 forms α-helical CCs driving LLPS. Finally, we observed that polyQ length variation impacts both CC structure and LLPS, ultimately regulating the transcriptional activity of FOXP2. These findings open new perspectives on the molecular underpinnings of vocalization and its evolution.

### Molecular encoding of vocalization frequency in the length of polyQ repeats in FOXP2 and FOXP1

Our analyses indicate that the frequency range of laryngeal vocalization in mammals is quantitatively encoded by FOXP2 polyQ lengths, so that lower Q_1_/Q_2_ length ratios indicate higher frequencies, and vice versa.

The correlation that we found between FOXP2 polyQ lengths and laryngeal vocalization frequency, or related morpho-functional parameters, was overall considerably robust and generalized in Chiroptera and, more broadly, in Mammalia, with some outliers at the species or taxon level. In this latter respect, two taxa that appeared to be outliers in this overall correlation either use non-laryngeal forms of USVs, i.e., Odontoceti, or display a parallel system of frequency encoding in the polyQ repeats of FOXP1, i.e., Muridae. The latter form of encoding based on the FOXP1 polyQ repeats is prevalent in Rodentia and Afrotheria, and is limited to a few species in other mammalian taxa.

Some previous studies have shown qualitatively how the species-specific occurrence of amino acid repeats can be related to interspecific divergence,^107^^,^^108^^,^^109^ but have not analyzed the effect of their length variation across species. Other studies have instead identified quantitative correlations of polyQ length variants with morphological or physiological parameters^9^^,^^110^ but only among individuals of the same species. Our findings extend these analyses by systematically studying the length variation of amino acid repeats in a large number of species, identifying an overall quantitative correlation between polyQ lengths and vocalization frequency, and extensive patterns of polyQ convergent evolution across mammalian taxa.

### Convergent evolution of polyQ repeats

Our evolutionary analyses identify extensive patterns of polyQ length convergence at the molecular level that correspond, at the organismal level, to convergence in vocalization/hearing frequency ranges across mammals.

In molecular evolution studies, convergence is generally defined in terms of single amino acid substitutions which can only be associated qualitatively, as a binary feature, to the presence of a certain phenotype, such as ultrasonic vocalization.^48^ Conversely, in our analyses, polyQ length variation could be quantitatively related overall to vocalization/hearing frequency ranges. At the same time, the polyQ sum-*vs*-ratio plots also captured qualitatively the nuanced vocalization features of Chiroptera (NE, FM_1_, FM_2_, CF). Therefore, this analytical approach offers powerful quantitative and qualitative tools in molecular evolution studies.

A growing body of evidence indicates that polyQ repeats are enriched in neural proteins, displaying complex evolutionary dynamics throughout phylogenesis.^36^^,^^40^^,^^41^^,^^111^ The evidence of convergent evolution adds a novel element to our appreciation of the complex evolutionary history of polyQ repeats.

The patterns of polyQ convergence between species in relation to vocalization/hearing frequency can be detected within and between mammalian orders. This is already evident in Chiroptera, in which we analyzed >50 species, eight of which display phenotypic convergence in USV type with species not belonging to their same superfamily. In six of them, this is paralleled by polyQ convergence in FOXP2, while in *Pteronotus parnellii* and *Noctilio leporinus* we observed polyQ convergence with USV-emitting rodents in FOXP1. *P. parnellii* (Noctilionoidea), which emits CF USVs, also has a unique Q-to-P substitution extending the predicted loop between Q_1_ and Q_2_, as in CF-emitting Rhinolophoidea. Strikingly, similar polyQ convergence patterns in FOXP2 and/or FOXP1 were found between species in most mammalian orders. Thus, these findings identify a first case of extensive molecular convergence associated with vocalization-related organismal phenotypes.

In mammals, multiple cases of molecular convergence of single residue substitutions are known in hearing-related proteins (e.g., *prestin*) in taxa with high-frequency vocalization/hearing, i.e., echolocating Chiroptera and Cetacea.^48^^,^^49^^,^^50^^,^^51^^,^^52^^,^^112^ However, none of the convergent mutations in these individual proteins identifies all the echolocating mammals in other taxa.^51^^,^^52^ Our findings highlight widespread polyQ convergence related to vocalization/hearing frequency across mammals, over the entire infrasonic to ultrasonic frequency range.

In contrast to hearing-related proteins, evidence of convergent molecular evolution in vocalization-related proteins is limited. Besides our findings, there is evidence of convergence between Chiroptera and Cetacea in muscle proteins involved in USV emission.^53^ For FOXP2, convergence between echolocating Chiroptera and Cetacea has not been observed,^28^ which may be related to the distinct USV emission modalities used by species belonging to these two taxa. Indeed, Chiroptera generate laryngeal USVs emitted through the mouth or nose, whereas more stereotyped (ultra)sonic clicks in Odontoceti are generated by non-laryngeal phonic lips and emitted through the frontal lipid-filled melon.^28^ The latter route does not require rapid orofacial and sensorimotor coordination, processes in which FOXP2 is implicated.^12^^,^^113^ Thus, USV emission in Cetacea may require molecular adaptations in genes other than FOXP2, which underwent accelerated evolution in Chiroptera.^28^ Our findings, in showing no polyQ convergence between Chiroptera and Cetacea (Odontoceti), are thus consistent with this view and with the notion that FOXP2 is more closely linked to the evolution of laryngeal vocalization.

Interestingly, polyQ/polyA variation in RUNX2 has been related to cranial morphological parameters in certain orders, like Carnivora and Primates,^39^^,^^114^^,^^115^ but not in others,^116^^,^^117^ indicating taxon-specific roles of the RUNX2 repeats. The patterns of FOXP2 polyQ convergence instead extend across mammals at large, although some degree of taxon specificity emerged, as for some USV-emitting Rodentia. Similarly, substantial variation in FOXP1 polyQ repeats is generalized in Rodentia and Afrotheria but restricted to fewer species in other taxa (Chiroptera, Lagomorpha, Eulipotyphla). Interestingly, some species display USV-related polyQ changes in both FOXP2 and FOXP1.

The case of the pika (*O. princeps*) highlighted the predictive power of FOXP2 polyQ lengths for vocalization/hearing frequency ranges. Indeed, its polyQ repeats converge with those of USV-emitting bats, in striking parallelism with the known pika-bat similarity in the evolution of the hearing-related protein *prestin*.^72^ We found that wild *O. princeps* emit calls extending into the ultrasonic range and has morphological cochlear parameters indicative of high-frequency hearing,^73^ These findings rationalize the pika-bat convergence in both FOXP2 and *prestin*.

### Coiled coils, LLPS, transcription, and their polyQ length-dependent regulation

To understand mechanistically how the evolutionary changes of the polyQ stretches may affect the FOXP2 protein, we first analyzed the structural and functional features of these repetitive sequences, and then defined the impact of their length variation at the molecular level.

We found that the two major polyQ repeats and the interposed hinge peptide form α-helical CCs with length-dependent stability. Indeed, CD analyses of two polyQ peptide variants showed that both exist in an equilibrium between random coil, α-helical, and CC conformations, that can be shifted by polyQ length variation, in agreement with existing evidence on polyQ sequences.^30^^,^^36^^,^^95^^,^^118^^,^^119^ These results, consistent with atomic-level structural predictions, support a model in which the two polyQ repeats form α-helices separated by a loop (hinge peptide) assembling into an antiparallel CC hairpin. The extension of the loop, and thus CC stability, can be varied by interspersed proline residues, which appeared recurrently throughout phylogenesis in Q_1_ or, in the unique case, within the hinge peptide of *P. parnellii* which contains two proline residues instead of one as in other species. Remarkably, besides the mere polyQ expansion/contraction, Q-to-P substitutions and polyP insertions, are virtually the only evolutionary changes that we observed in the FOXP2 polyQ repeats. This can be rationalized by considering that proline destabilizes α-helices and CC-driven oligomerization.^30^^,^^120^^,^^121^ Proline insertions in Q_1_ may thus represent a recurrent molecular adaptation regulating CC length, stability, and oligo-/polymerization.

Overall, variable combinations of Q_1_ and Q_2_ length changes lead to a graded variation of their total and relative lengths. Such polyQ CC length variation may represent a structural rearrangement ultimately regulating protein function,^32^^,^^33^^,^^36^ a notion that is supported by our findings.

The AFM morphological analysis of the assemblies formed by FOXP2 polyQ peptides revealed both rounded mesoscale condensates and long fibrillary structures with transitional figures, as found in AFM studies of LLPS-prone proteins.^98^ Remarkably, the relative proportion of condensates and fibrils is modulated by polyQ length and by a helix-stabilizing agent (TFE). In CD spectra, TFE enhanced the α-helical/CC signal, which displayed signatures associated with fibrillary CC polymers. These findings together indicate that α-helix/CC stability is closely related to the oligo-/polymerization modalities of FOXP2 polyQ peptides. Thus, while relatively unstable α-helices/CCs may drive LLPS through less regular interactions between protomers (e.g., CC assembly by helix swapping),^30^ more stable α-helices/CCs may promote fibrillization through more regular structural arrangements (e.g., CC oligomer stacking).^30^ This model is consistent with the observation of transitional profiles between condensates and fibrils and with the notion that LLPS may represent a nucleation step triggering fibrillization.^100^

The AFM findings of condensate-like structures were confirmed in the cellular environment. Indeed, both endogenous and exogenously expressed FOXP2 form discrete, 1,6-HEX-sensitive intranuclear foci which undergo rapid FRAP, as typical of LLPS-driven condensates.^103^ The polyQ-bearing NTD (and its Q_1_-Q_2_ fragment) recapitulated the phase separation behavior of the entire protein. Moreover, the NTD alone was also able to recruit FOXP1 into condensates. These findings concurrently identified the NTD, and its polyQ repeats as the drivers of FOXP2 LLPS. Remarkably, human FOXP2 mutants in which the polyQ repeat lenghts were changed to mimic their evolutionary variation displayed differential ability to undergo LLPS as a function of total and relative polyQ lengths.

These findings are consistent with the emerging ability of polyQ and other amino acid repeats in transcription factors to drive LLPS, as shown for the polyQ/polyA repeats of RUNX2.^35^ Notably, we had found that also these repeats in RUNX2 form α-helical CCs, with length-dependent stability, that regulate protein oligo-/polymerization and transcriptional activity.^36^ Thus, the evolutionary length variation modulating CC stability may be a general mechanism to regulate transcription factor activity.^32^^,^^33^^,^^36^

This interpretation is supported by our observation that, besides LLPS, the FOXP2 transcriptional activity is also related to the total and relative polyQ lengths. Indeed, lower Q_1_+Q_2_ and Q_1_/Q_2_ were associated with higher repressive activity on the target SRPX2 promoter and vice versa. Only two studies have previously analyzed the possible regulatory roles on transcription of the FOXP2 polyQ repeats with conflicting results.^122^^,^^123^ These studies focused only on repeats deletions, one of which is very modest (i.e., a single Q deletion), and did not test a series of polyQ length variants. Estruch et al.^123^ reported that polyQ repeats do not significantly regulate transcription, despite some variable effects that were observed. However, in this study the luciferase experiments were performed using FOXP2 variants with N-terminal YFP tags, which may interfere by steric hindrance with the NTD structure and function. This may explain the small, variable transcriptional effects observed upon polyQ deletion. Conversely, Zhao et al.^122^ proposed that a single Q deletion may abolish the repressive effect of FOXP2 on the CNTNAP2 promoter. However, the same authors reported very different expression levels of the FOXP2 variants in their cell lines, which correlated with CNTNAP2 levels, putting into question their conclusion. Thus, a single Q deletion would have no effect when normalizing for expression levels (see also Estruch et al.^123^). Possible cell type- and promoter-specific transcriptional effects of FOXP2^124^ should also be taken into account in comparing the results of the different studies.

Remarkably, our results are instead similar to those obtained in experiments studying a FOXP1 variant devoid of its polyQ repeats, which displayed enhanced repressive activity.^125^ Moreover, a recent comparison of the transcriptional activity of multiple polyQ variants in the androgen receptor (AR) highlighted length-dependent transcriptional effects.^126^ These and our findings suggest the possibility that the more LLPS-prone polyQ variants of FOXP2 may be more easily retained into condensates and less able to bind target promoters, thus limiting their regulatory effect on transcription, although alternative mechanisms cannot be ruled out.

### Toward a quantitative understanding of the molecular underpinnings of mammalian vocalization

A growing body of evidence indicates that polyQ and other amino acid repeats can modulate in a length-dependent manner, the structure, interactions, and function of proteins.^30^^,^^31^^,^^33^^,^^36^^,^^40^^,^^111^ The presence and length variation of these ‘tuning knobs’ in proteins can have important evolutionary implications.^36^^,^^37^^,^^38^^,^^40^^,^^127^ Consistent with these views, the results of our analyses provide evidence that the observed evolutionary polyQ length variation regulates the structure, LLPS, and transcriptional activity of FOXP2.

Taken together, our findings uncover a quantitative molecular encoding of vocalization frequency in the relative lengths of polyQ repeats in FOXP2, highlight widespread patterns of vocalization-related polyQ convergent evolution, and rationalize mechanistically how polyQ length variation can have phenotypic impact by regulating the molecular function of FOXP2.

These results and analytical approaches contribute to a quantitative, mechanistic understanding of vocalization and its evolution.

### Limitations of the study

Our analyses of the molecular evolution of FOXP2 and FOXP1 polyQ repeats in relation to vocalization frequency-related parameters were deliberately focused on Mammalia, given their considerable degree of polyQ length variability and their highly diversified vocalization modalities and frequency ranges. Thus, the study did not include some non-mammalian taxa, such as Aves, in which a considerable number of species are known to emit highly sophisticated and diversified forms of vocalization. We are currently exploring whether the results of our analyses can also apply to these non-mammalian taxa.

## STAR★Methods

### Key resources table

**Table:** 

REAGENT or RESOURCE	SOURCE	IDENTIFIER
**Antibodies**		

Anti-FOXP2 antibody	Thermo Fisher Scientific	Cat#720031; RRID: AB_2610345
Alexa 488-conjugated anti-rabbit secondary antibody	Thermo Fisher Scientific	Cat#A-11008; RRID: AB_143165

**Bacterial and virus strains**		

Stellar chemically competent cells	Clontech-Takara	Cat#636763
TOP10 chemically competent cells	Thermo Fisher Scientific	Cat#C404010

**Chemicals, peptides, and recombinant proteins**		

Fetal bovine serum (FBS)	Sigma-Aldrich	Cat#F7524-500ML
L-glutamine	Sigma-Aldrich	Cat#59202C
Penicillin-streptomycin	Sigma-Aldrich	Cat#P4333-100ML
MEM non-Essential amino acids	Gibco	Cat#11140035
Sodium chloride	Euroclone	Cat#EMR211500
Sodium dihydrogen phosphate	Sigma-Aldrich	Cat#8210-500GM
Sodium phosphate dibasic	Sigma-Aldrich	Cat#S7907-100MG
2,2,2-trifluoroethanol	Sigma-Aldrich	Cat#91683-25ML
*Min-sch*-Q1Q2 custom peptide	Thermo Fisher Scientific	N/A
*Rhi-fer*-Q1Q2 custom peptide	Thermo Fisher Scientific	N/A
Mica (V1 grade muscovite)	Ted Pella	Cat#PEL56-100
PrimeSTAR-GXL-SP DNA Polymerase	Clontech-Takara	Cat#RF220Q
BamHI restriction enzyme	New England Biolabs	Cat#R0136L
NotI restriction enzyme	New England Biolabs	Cat#R0189L
XhoI restriction enzyme	New England Biolabs	Cat#R0146L
T4 ligase	New England Biolabs	Cat#M0202L
QIAquick kit	Qiagen	Cat#28106
QIAprep Spin Mini-prep kit	Qiagen	Cat#27104
QIAprep Spin Maxi-prep kit	Qiagen	Cat#12663
NucleoSpin plasmid Mini kit	Macherey-Nagel	Cat#FC140588N
NucleoBond Xtra MIDI EF kit	Macherey-Nagel	Cat# FC140420L
Poly-L-lysine	Sigma-Aldrich	Cat#P2636
Falcon 50 mm cell culture dishes	Corning	Cat#351006
Sylgard 184	Dow Corning	Cat#2646340
96-well white bottom plates	Thermo Fisher Scientific	Cat#136101
Fugene-6	Promega	Cat#E2691
Lipofectamine 2000	Thermo Fisher Scientific	Cat#11668019
Phosphate-buffered saline	Sigma-Aldrich	Cat#F7524-500ML
Paraformaldehyde	Pamreac	Cat#1414511211
Dako fluorescent mounting medium	Agilent	Cat#S302380-2
Triton X-100	Sigma-Aldrich	Cat#11332481001
Bovine serum albumin	Sigma-Aldrich	Cat#9418-50G
DAPI	Thermo Fisher Scientific	Cat#D1306
1,6-hexanediol	Sigma-Aldrich	Cat#240117-50G
Tween 20	Euroclone	Cat#EMR390025
HBSS	Sigma-Aldrich	Cat#H6648-500ML

**Critical commercial assays**		

In-Fusion Snap Assembly Master Mix	Clontech-Takara	Cat#638948
Dual-Glo luciferase assay system	Promega	Cat#e2929
GeneArt gene synthesis service	Thermo Fisher Scientific	N/A

**Deposited data**		

FOXP2 and FOXP1 protein sequences	Uniprot database; www.uniprot.org	See Table S1
FOXP2 and FOXP1 protein sequences	NCBI database; www.ncbi.nlm.nih.gov	See Table S1
FOXP2 and FOXP1 genomic sequences	NCBI database; www.ncbi.nlm.nih.gov	See Table S1
Mammal Diversity Database https://www.mammaldiversity.org	https://doi.org/10.5281/zenodo.7830771	N/A
Archived pika vocalization recordings, Columbia River Gorge (OR, USA). Cascades Pika Watch; https://scistarter.org/cascades-pika-watch	https://databasin.org/datasets/69254dafe2dd44aba25ec834f4a8a359/	Dataset #: 69254dafe2dd44aba25ec834f4a8a359
Vocalization frequency and body mass data (Chiroptera)	Collen (2012)^55^	N/A
Cochlear and basicranium widths (Chiroptera)	Simmons et al. (2008)^58^	N/A
Auditory sensitivity (60 dB SPL) (Rodentia)	Heffner lab., Univ. of Toledo (OH, USA); https://www.utoledo.edu/al/psychology/research/psychobio/comphearinglab.html	N/A
Vocalization freq. data (Mammalia)	Charlton and Reby (2016)^128^	See Table S2
Vocalization freq. data (Mammalia)	Bowling et al. (2017)^129^	See Table S2
Vocalization freq. data (Mammalia)	Martin et al. (2017)^83^	See Table S2
Vocalization freq. data (Mammalia)	He et al. (2021)^68^	See Table S2
Body mass data (Mammalia)	Milton and May (1976)^130^	See Table S3
Body mass data (Mammalia)	Riek and Geiser (2013)^131^	See Table S3
Body mass data (Mammalia)	Smith et al. (2003)^132^	See Table S3
Body mass data (Mammalia)	White and Seymour (2003)^133^	See Table S3
Body mass data (Mammalia)	Khaliq et al. (2014)^134^	See Table S3
Body mass data (Mammalia)	Hirt et al. (2017)^135^	See Table S3
Body mass data (Mammalia)	Martin et al. (2017)^83^	See Table S3
Body mass data (Mammalia)	Encylopedia of Life; https://opendata.eol.org/dataset/all-body-size-data	See Table S3
Available micro computed tomography (*μ*CT) scans	MorphoSource database; https://www.morphosource.org	See Table S4
Available silhouettes of mammalian species	PhyloPic; http://phylopic.org	See Table S5
*Images of M. unguiculatus and O. degus* to generate silhouettes	Wikimedia; https://commons.wikimedia.org	Meriones_unguiculatus-front.jpg Octodon_degus_2.jpg

**Experimental models: Cell lines**		

HEK293 cells (293-F strain)	Thermo Fisher Scientific	Cat#293-F
Neuro-2a cells	G. Merlo, University of Turin	N/A

**Recombinant DNA**		

pcDNA4/HisMax-FOXP2-*Hs* plasmid	Dr. P. Szepetowski (INSERM, FR); Roll et al. (2010)^106^	N/A
pcDNA4/HisMax-FOXP2-*Cs* plasmid	This study	N/A
pcDNA4/HisMax-FOXP2-*Ms* plasmid	This study	N/A
pcDNA4/HisMax-FOXP2-*Rf* plasmid	This study	N/A
pcDNA4/HisMax-FOXP2-ΔQ_1_Q plasmid	This study	N/A
pcDNA4/HisMax plasmid	Thermo Fisher Scientific	Cat#V86420
pEGFP-C1 plasmid	Clontech-Takara	Cat#6084-1
pGL3-SRPX2 plasmid	Dr. P. Szepetowski (INSERM, FR); Roll et al. (2010)^106^	N/A
pRL-CMV plasmid	Promega	Cat#e2261
pcDNA4/HisMax-FOXP1 plasmid	Dr. A. Banham (Univ. of Oxford, UK)	N/A
pDsRed-monomer-N1 In-Fusion Ready plasmid	Clontech-Takara	Cat#632498
pDsRed-monomer-N1-FOXP1 plasmid	This study	N/A
pAcGFP1-N In-Fusion Ready plasmid	Clontech-Takara	Cat#632501
pAcGFP1-N- FOXP2-*Hs* plasmid	This study	N/A
pAcGFP1-N- FOXP2-*Cs* plasmid	This study	N/A
pAcGFP1-N- FOXP2-*Ms* plasmid	This study	N/A
pAcGFP1-N- FOXP2-*Rf* plasmid	This study	N/A
pAcGFP1-N- FOXP2-NTDcc*-Hs* plasmid	This study	N/A
pAcGFP1-N- FOXP2-NTDcc-*Cs* plasmid	This study	N/A
pAcGFP1-N- FOXP2-NTDcc-*Ms* plasmid	This study	N/A
pAcGFP1-N- FOXP2-NTDcc-*Rf* plasmid	This study	N/A
pDsRed-Monomer-N1	Clontech-Takara	Cat#632465
pHR-mCh-Cry2WT plasmid	Addgene (Shin et al., 2017)^101^	Cat#101221
pX-mCh-Cry2 plasmid	This study	N/A
pX-mCh-Cry2-NTDcc-*Hs* plasmid	This study	N/A
pX-mCh-Cry2-NTDcc-*Hs-* Q_1_Q_2_ plasmid	This study	N/A
pX-mCh-Cry2-NTDcc-*Hs-* ΔQ_1_Q_2_ plasmid	This study	N/A
pX-mCh-Cry2-NTDcc-*Cs* plasmid	This study	N/A
pX-mCh-Cry2-NTDcc-*Ms* plasmid	This study	N/A
pX-mCh-Cry2-NTDcc-*Rf* plasmid	This study	N/A

**Software and algorithms**		

PolyQ analyzer Perl script	Pelassa et al. (2014,2015)^36^^,^^136^	N/A
MultAlin	Corpet (1988)^137^; http://multalin.toulouse.inra.fr/multalin	N/A
MyDomains	Hulo et al. (2013)^138^; https://prosite.expasy.org/mydomains	N/A
TimeTree	Kumar et al. (2017)^139^; http://www.timetree.org	N/A
Mega X	Kumar et al. (2018)^140^; https://www.megasoftware.net	N/A
ImageJ	Schneider et al. (2012)^141^; https://imagej.nih.gov/ij/download.html	N/A
Photoshop Elements 11	Adobe	N/A
Aleph 3D-viewer	Embedded in the MorphoSource website; www.morphosource.org	N/A
ITK-SNAP 3.8.0	Yushkevich et al. (2006)^142^	N/A
Kaleidoscope Lite 5.4.7	Wildlife Acoustics	N/A
DeepCNF	Wang et al. (2016)^143^; embedded in the Quick2D platform; https://toolkit.tuebingen.mpg.de	N/A
Paircoil2	McDonnell et al. (2006)^90^; https://cb.csail.mit.edu/cb/paircoil2	N/A
Raptor-X	Wang et al. (2016)^143^; http://raptorx6.uchicago.edu	N/A
UCSF Chimera	Pettersen et al. (2004)^144^; https://www.cgl.ucsf.edu/chimera	N/A
Spectra Manager (version 2.06.0)	JASCO Corporation	N/A
Gwyddion	Necas and Klapetek (2012)^145^; http://gwyddion.net	N/A
Fluoview	Olympus	N/A
LAS-X	Leica	N/A
ZEN lite	Zeiss	N/A
*Easy-FRAP*	Koulouras et al. (2018)^146^; https://easyfrap.vmnet.upatras.gr	N/A
CellProfiler	Stirling et al. (2021)^147^; https://cellprofiler.org	N/A
Excel	Microsoft	N/A
Statistica	Tibco	N/A
Python 3	Available at https://www.python.org	N/A

**Other**		

Vocalization recorder	Wildlife Acoustics	SM3BAT
Spectropolarimeter	Jasco	J-815
Al-coated silicon cantilever	Budget Sensors	Cat#Tap190Al-G
Atomic force microscopy	Nanosurf	Easyscan2
Fluorescence microscope	Nikon	Eclipse TE200
Confocal microscope	Olympus	FV300
Confocal microscope	Leica	TCS SP5
Confocal microscope	ZEISS	LSM 800 Airyscan
Microplate reader	Promega	GloMax

### Resource availability

#### Lead contact

Further requests for information, resources, and reagents should be directed to and will be fulfilled by the lead contact, Ferdinando Fiumara (ferdinando.fiumara@unito.it).

#### Materials availability

Reagents generated as part of this study, as listed in the key resources table, are available from the lead contact upon request.

### Experimental model and subject details

#### Cells

HEK293 cells (293-F strain; Thermo Fisher) and Neuro-2a cells (a kind gift of G. Merlo, University of Turin) were maintained following standard procedures at 37°C with 5% CO_2_ in DMEM (Thermo Fisher) supplemented with 10% fetal bovine serum, 2 mM L-glutamine, 100 units/mL penicillin, 100 μg/mL streptomycin, and, for Neuro-2a cells, MEM non-Essential amino acids solution (1X; Gibco).

### Method details

#### Bioinformatics

PolyQ lengths were quantified using a polyQ analyzer Perl script^36^^,^^136^ in protein sequences of FOXP1/2 orthologs from Uniprot (www.uniprot.org/) or NCBI (www.ncbi.nlm.nih.gov/protein/). For certain species, polyQ lengths were manually determined by translating genomic sequences identified through BLAST searches on the NCBI genome platform (https://www.ncbi.nlm.nih.gov/genome/) using human FOXP2 or FOXP1 sequences as the query. The list of species for which FOXP1/2 polyQ repeats were analyzed, their name abbreviations, and the corresponding protein or genomic sequence IDs are listed in Table S1. For each FOXP1/2 ortholog, we calculated the length of the two polyQ repeats (‘Q_1_’ and ‘Q_2_’) corresponding in sequence alignments to the two major repeats flanking the hinge peptide ‘ALQVARQLLL’ in human FOXP1 or ‘HPGKQAKE’ in human FOXP2. In a few predicted FOXP2 sequences of Cetacea species, the hinge peptide is extended by a likely spurious 22-residue peptide (‘VGSGRLTHAEEGEAGRGPRRPG’ in *T. truncatus*, with minor substitutions in other species), which is not present in other cetacean or mammalian orthologs. It is encoded by a genomic sequence immediately downstream of a conserved splice site located at the end of the sequence encoding the canonical hinge octapeptide. This splice site may have been missed in the computational identification of FOXP2 exon-intron junctions in certain Cetacea genomes. Sequencing of FOXP2 cDNAs from Cetacea will be required to address this uncertainty in a conclusive manner. Anyhow, the presence or absence of this peptide does not change Q_1_ and Q_2_ lengths in these predicted ortholog sequences, which were therefore included in our analyses.

For each ortholog, we calculated the Q_1_+Q_2_ sum and the Q_1_/Q_2_ ratio. In the case of multiple available sequences/isoforms for each species, we analyzed the sequence with the longest polyQ tracts. The Q_1_ repeats of FOXP2 are interrupted by one or more proline residues in several species. In these species, given the structural effect of proline residues,^30^^,^^36^ we calculated the Q_1_ length as the length of the uninterrupted polyQ repeat upstream of the first proline residue, while fragmented repeats between this proline residue and the hinge peptide were not taken into account. In a few cases, a single histidine residue was occasionally found within Q_1_. Given the polar nature of the residue, it was considered as being part of the polar polyQ stretch rather than an interruption of it.

Sequence alignments were generated using MultAlin.^137^ Protein domain schemes were obtained from MyDomains^138^ and modified using Photoshop Elements 11 (Adobe). Phylogenetic trees were derived from TimeTree^139^ and elaborated using Mega X.^140^ Silhouette drawings in the public domain of species of interest were downloaded and modified from PhyloPic (http://phylopic.org). *M. unguiculatus* and *O. degus* silhouettes were derived from Wikimedia (https://commons.wikimedia.org; pictures by Pacos and Algesirdas).

#### Phenotypic datasets

Vocalization modalities, vocalization frequency/bandwidth, and body mass for Chiroptera were derived from Collen.^55^ The relative cochlear size (CW/BW in Figure 1D) for chiropteran species was derived from Simmons et al.^58^ The maximum audible frequency (60 dB SPL) in audiograms of rodent species of interest was obtained from the Heffner laboratory at the University of Toledo (OH, USA; https://www.utoledo.edu/al/psychology/research/psychobio/comphearinglab.html). For 4 species, i.e., *Dipodomys merriami*, *Marmota monax, Onychomys leucogaster*, and *Spalax ehrenbergi,* we used audiograms of closely related species in the same genus, i.e., *Dipodomys ordii*, *Marmota marmota*, *Onychomys torridus* and *Spalax galili*, respectively. The vocalization frequencies for non-chiropteran species of interest were derived from Charlton and Reby,^128^ Bowling et al.,^129^ Martin et al.,^83^ He et al.,^68^ and body mass data from Milton and May,^130^ Riek and Geiser,^131^ Smith et al.,^132^ White and Seymour,^133^ Khaliq et al.,^134^ Hirt et al.,^135^ Martin et al.,^83^ and the Encyclopedia of life (EOL; https://opendata.eol.org/dataset/all-body-size-data; Tables S2 and S3). As the Collen^55^ dataset on USVs in Chiroptera may potentially overestimate the minimum vocalization frequency, the analyses correlating this parameter with polyQ lengths in mammals were performed either including (see Figure 3D) or excluding (see Figure S4A) Chiroptera. Both analyses gave similar significant results. When a parameter was reported for a given species in multiple datasets, the maximum (for maximum vocalization frequency and body mass) or minimum value (for minimum/fundamental vocalization frequency) was selected for the correlation analyses.

To calculate the correlation between the mean values of polyQ-related parameters and vocalization-/hearing-related parameters (Figures 1D, 3D, and 3E), the species were divided in groups based on increasing values of either one of the parameters. In Figure 1D, species (n = 43) were divided in 8–9 groups based on characteristic frequency (bin 1: ≤30 kHz, bins 2–7; between 30 and 90 kHz in bins of 10 kHz; bin 8: >90 kHz), minimum frequency (bin 1: ≤20 kHz; bins 2–7; between 20 and 80 kHz in bins of 10 kHz; bin 8: >80 kHz), bandwidth (bin 1: ≤5 kHz, bins 2–8; between 5 and 40 kHz in bins of 5 kHz; bin 10: >40 kHz), CW/BW (species were ranked based on increasing CW/BW (range 0.13–0.37) and binned in 8 groups of 5 species each). In Figures 3D and 3E, species (n = 107 and n = 83, respectively) were binned in 10 groups based on their Q_1_/Q_2_ ratio (bin 1: ≤1, bins 2–9: between 1 and 5 in bins of 0.5, bin 10: >5). In Figure S4A, species (n = 66 for the body mass and minimum frequency data; n = 42 for the maximum frequency data) were binned in 6 groups based on their Q_1_/Q_2_ ratio (bin 1: ≤3, bins 2–5: between 3 and 5 in bins of 0.5, bin 6: >5).

#### Morphological analyses on μCT scan analyses

Micro computed tomography (*μ*CT) scans encompassing the cranial region of species of interest were obtained from the MorphoSource database (https://www.morphosource.org; Media IDs: 86463,96992,2150,170220,170399,170595,165459,02723,158814,13131,11011,10956,168375,15460,15477,15612,15720,410414,394893,140436,8620,114319,56554,22387,16145,16139,16131,13626,22292,22235,21964,21967,21967,21952,45544,167374,55626,55624,47005,167378,55698,79025,367903,47052,55618,55349,167384,365356,167937,36587,57664,167934,36439,36171,25920,25943,36828,167937,36257,36434,36511,36128,57668,S9128; Table S4; The *P. alecto* scan S9128 was used only to generate the *left panel* in Figure 1C). For each scan, the image stack was cropped to the region of interest, converted into 8-bit format, and saved in Nifti format using ImageJ.^141^ The cropped scans were used to generate digital endocasts of the cochlea and lateral semicircular canals using ITK-SNAP after segmentation based on manual thresholding. Cursors were positioned on landmarks of interest and 3D coordinates were used to calculate distances between them, and their ratios, using Excel. For each scan, we measured the cochlear height-to-width ratio^62^ and the coclear width/basicranium width (CW/BW) ratio.^58^ For most Primates/Lagomorpha species, mean values of these parameters were obtained from 2 or more specimen for each species. In the case of Leporidae, the mean values were derived from one specimen each of *Lepus europaeus*, *L. articus*, *L. americanus*, and correlated with available polyQ parameters derived from *L. europaeus*. For Ochotonidae, the mean values were derived from three specimens of *Ochotona princeps* and one of *O. pallasi* and correlated with the available polyQ parameters from *O. princeps*. For Chiroptera, the morphological parameters were obtained from a single specimen of each species of interest (see Table S4).

#### Vocalization detection and analysis

To analyze spontaneous vocalizations of wild American pikas (*Ochotona princeps*), we analyzed archived recordings originally taken as part of a monitoring project (Cascades Pika Watch; https://scistarter.org/cascades-pika-watch) of a resident population at talus slopes in the Columbia River Gorge (OR, USA). Pika vocalizations were recorded using an acoustic microphone with some sensitivity to ultrasonic signals (up to 25 kHz) connected to a SM3BAT recorder (Wildlife Acoustics). The recordings that were analyzed, showing evidence of pika vocalizations extending in the ultrasonic range with harmonics >20 kHz (see Figure 2D), were recorded between May 29 and June 5, 2017 (databasin.org, “2017 Western Columbia River Gorge” dataset), using a microphone located near Herman Creek (microphone coordinates: 45° 40' 24. 0384", 121° 50' 15.288"). The vocalization spectrograms were analyzed using the Kaleidoscope software (Wildlife Acoustics).

#### Structural predictions

Predictions of FOXP2 secondary structure were obtained using DeepCNF^143^ on the Quick2D platform (https://toolkit.tuebingen.mpg.de/). Predictions of coiled coil structures were obtained using Paircoil2.^90^ Atomic-level structural models for a.a. 1–244 of the human ortholog and for the corresponding regions, based on sequence alignments, of the *R. ferrumequinum* (1–240), *M. schreibersii* (1–232), and *C. sphinx* (1–264) FOXP2 orthologs, were obtained using RaptorX^92^ and visualized using UCSF Chimera.^144^

#### Peptide synthesis and circular dichroism (CD)

Synthetic peptides encompassing the FOXP2 polyQ region (primary sequence in Figure 4A) of the *R. ferrumequinum* and *M. schreibersii* orthologs were chemically synthesized (Thermo Fisher Scientific) with N-terminal acetylation, C-terminal amidation and purity >95%. Peptides were dissolved in a saline buffer (100 mM NaCl, 10 mM phosphate buffer, pH 7.4)^30^ to generate stock solutions (1 mg/mL) which, after the circular dichroism measurements, were aliquoted, flash-frozen in liquid N_2_, and stored at −80°C. The *Miniopterus*-derived peptide formed an opalescent solution whose concentration was difficult to measure spectrophotometrically. Thus, peptide concentrations in circular dichroism experiments were estimated based on peptide weight. This may overestimate the concentration of soluble, optically active chiral material and thus underestimate the ellipticity signal.^95^ Before each measurement, peptides were further diluted in saline buffer to a working concentration of 0.2 mg/mL, transferred to quartz cuvettes (1-mm optical length), and CD spectra were collected in the 190–260 nm frequency range (every 0.5–1 nm) using a J-815 spectropolarimeter (Jasco). In some measurements peptides were diluted in saline buffer containing 50–80% v/v 2,2,2-trifluoroethanol (TFE, Sigma-Aldrich). Blank spectra of saline buffer, or of saline buffer with TFE, were subtracted from the peptide spectra in the same buffers. The mean residue molar ellipticity [*θ*] was calculated as [*θ*] = *θ* × *mw*/(10 × (*n*–1) × *c* × *pl*), where *θ* is the measured ellipticity, *mw* is the molecular weight of the peptide, *n* is the number of amino acids in the peptide, *c* is the concentration of the peptide (mg/mL), and *pl* is the cuvette pathlength (cm).^148^ To test structural stability, the samples were heated (from 5°C to 75°C; 10 °C/min) while recording ellipticity at 222 nm. Data and graphs were elaborated using Spectra Analysis (Jasco) and Excel (Microsoft).

#### Atomic force microscopy (AFM)

AFM scans were performed under ambient conditions in tapping mode by using a sharp Al-coated silicon cantilever (Tap190Al-G, Budget Sensors; length: 225 μm; width: 38 μm; tip radius: ca. 10 nm, 10° at the apex), near the resonance frequency (190 kHz), mounted on an Easyscan2 AFM (Nanosurf) equipped with a high resolution 10-μm scan head, within a shielded and insulated enclosure placed on an antivibration platform, as in Pelassa et al.^36^ Peptide stocks in saline buffer, stored at −80°C, were thawed and diluted to 0.05–0.25 mg/mL in saline buffer either with or without 50–80% TFE. The diluted solutions were incubated at 4°C for 10–15 min and a few microliters of them were dropped onto freshly cleaved mica (V1 grade muscovite, Ted Pella) and let air dry for ∽5 min. The mica surface was gently rinsed with ultrapure water and dried under a mild stream of nitrogen. Samples were analyzed as soon as the surface became dry, within 1 h. In some measurements (Figure 5B), to facilitate oligo-/poly-merization thus enhancing condensate/fibril formation, the peptides were pre-incubated at 4°C for at least 72 h. Gwyddion^145^ was used to elaborate (leveling, row alignment), and generate zenithal views of 3-D renderings in orthographic projection (overlay mode), adjusting lighting to maximize the visibility of condensate/fibril profiles).

#### Plasmids and molecular cloning

The pcDNA4/HisMax-FOXP2 plasmid encoding human FOXP2 and the pGL3-SRPX2 containing the SPRX2 promoter were kindly provided by Dr. P. Szepetowski (INSERM, France).^106^ The pcDNA4/HisMax-FOXP1 plasmid encoding human FOXP1 was kindly provided by Dr. A. Banham (University of Oxford, UK). The pRL-CMV vector was obtained from Promega (#e2261). The pHR-mCh-Cry2WT plasmid^101^ was obtained from Addgene (#101221).

Plasmids encoding chimeric polyQ variants of human FOXP2, in which the sequence encoding Q_1_, Q_2_, and the hinge peptide were replaced by the corresponding sequence of chiropteran FOXP2 orthologs of interest, were obtained using the In-Fusion cloning system (Clontech-Takara). Briefly, two PCR products with overlapping ends (15 bp overlaps) were obtained using the PrimeSTAR-GXL-SP DNA Polymerase (Clontech). The first product consisted of the pcDNA4/HisMax-FOXP2 plasmid sequence devoid of the region encoding the two major polyQ repeats (residues 152–209 in the human protein). The second product contained a fragment of FOXP2 orthologs of species of interest (i.e., *C. sphinx* (*Cs*), *M. schreibersii* (*Ms*), or *R. ferrumequinum* (*Rf*)) overlapping with the human FOXP2 polyQ region fragment (a.a. 152–209) in FOXP2 ortholog sequence alignments.

For the *Cs* and *Ms* orthologs, these products were PCR-amplified using long, partially overlapping forward and reverse primers, encoding overall for the entire region of interest (as derived from NCBI). For the *Rf* ortholog, the second product was PCR-amplified from a plasmid encoding the region of interest previously obtained by gene synthesis (GeneArt, Thermo Fisher). All the three primer pairs used for the *Cs*, *Ms*, and *Rs* polyQ region contained 15 bp extensions, corresponding to the ends of the pcDNA4/HisMax-FOXP2-derived PCR product, suitable for cloning using the In-fusion system. Finally, the latter PCR product was alternatively combined with one of the *Cs*, *Ms* or *Rf* polyQ fragments in cloning procedures using the In-Fusion system to generate the pcDNA4/HisMax-FOXP2-*Cs*, -*Ms*, and -*Rf* plasmids: These plasmids were used, together with the original plasmid encoding the *Homo sapiens* (*Hs*) ortholog, that we called pcDNA4/HisMax-FOXP2-*Hs.* In a fifth construct (ΔQ_1_Q_2_), the polyQ region (a.a. 152–216, i.e., from the start of Q_1_ to a few residues after the end of Q_2_) was deleted by re-circularizing a pcDNA4/HisMax-FOXP2-derived PCR product upon ligation using a T4 ligase (NEB). These five plasmids (i.e., pcDNA4/HisMax-FOXP2-*Hs*, -*Cs*, -*Ms*, -*Rf*, -ΔQ_1_Q_2_) were then used for luciferase assay experiments. Moreover, they served as templates for the PCR amplification of sequences encoding full-length human FOXP2 polyQ variants (FOXP2-*Hs*, -*Cs*, -*Ms*, -*Rf*) with suitable 15 bp extensions for their In-Fusion cloning into the pAcGFP1-N In-Fusion Ready vector (Clontech-Takara; #632501) for the expression of the GFP-tagged polyQ variant proteins. These constructs were used for studying the subcellular distribution of FOXP2 polyQ variants (confocal fluorescence microscopy) and their transcriptional activity (luciferase assays). A set of plasmid constructs was similarly generated, using pAcGFP-N1-In-Fusion-Ready as the recipient plasmid, for the expression of GFP-tagged N-terminal domains (NTDs) of human FOXP2 and its polyQ length variants (*Cs*, *Ms*, *Rf*, and ΔQ_1_Q_2_), corresponding to the first 244 residues of human FOXP2 in ortholog protein alignments, which encompass the first three CC regions in the Paircoil2 prediction (*NTDcc* constructs; Figures 4A and 7A).

We generated a plasmid for the expression of FOXP1-DsRed by PCR-amplifying the FOXP1 coding region from the pcDNA4/HisMax-FOXP1 plasmid with suitable extensions for In-Fusion-cloning into the pDsRed-monomer-N1 In-Fusion Ready (Clontech-Takara, #632498).

To use the *optoDroplet* system for a spatiotemporally controlled induction of protein LLPS in living cells, we generated a vector for the cellular expression of proteins of interest fused to mCherry (mCh; for visualization) and to an *A. thaliana* Cry2 fragment (Cry2; for photoactivation).^101^ The DNA sequence coding for the mCh-Cry2 fusion was PCR-amplified from the pHR-mCh-Cry2WT plasmid (Addgene #101221), with 15 bp extensions suitable for In-Fusion cloning into a pDsRed-monomer-N1 vector (Clontech-Takara), from which we had previously enzymatically excised the dsRed coding sequence using BamHI and NotI (NEB). The resulting pX-mCh-Cry2 vector was linearized by digestion with XhoI (NEB) and used as a recipient vector for PCR fragments of interest bearing suitable 15 bp extensions for In-Fusion cloning and encoding protein fragments of interest to be expressed as fusions with mCh-Cry2. Using this approach, we generated constructs for the expression of the human FOXP2 NTDcc fragment, its sub-fragments (i.e., Q_1_Q_2_ (a.a. 146–221) and ΔQ_1_Q_2_), and its polyQ variants (*Cs*, *Ms*, *Rf*) as described above.

All PCR products in the cloning procedures were separated by agarose gel electrophoresis and bands of interest were purified using QIAquick (Qiagen) or the NucleoSpin (Macherey-Nagel) kits. Plasmids were amplified by transformation of Stellar (Clontech) chemically competent cells and purified using the QIAprep Spin Mini-/Maxi-prep kits (Qiagen), or the NucleoSpin plasmid Mini kit (Macherey-Nagel), or NucleoBond Xtra MIDI EF kit (Macherey-Nagel). For all plasmids that were generated, the coding sequences of FOXP2 and tags were verified by Sanger sequencing.

#### Cell culture transfection and fixation

For immunocytochemistry and imaging of fluorescently tagged proteins, cells were plated on poly-L-lysine-coated (0.1 mg/mL) glass coverslips within 24-well culture plates. For *optoDroplet* and FRAP experiments, cells were plated into poly-L-lysine-coated dishes obtained starting from Falcon 50 mm plastic cell culture dishes (Corning) that were perforated to produce a central hole (10 mm) then closed by a glass coverslip glued using Sylgard 184 (Dow Corning).^149^ For luciferase reporter assay, HEK293 cells were cultured in 96-well white plates (VWR).

Cell (co-)transfections with plasmid DNA were performed in 24 h after cell plating using either Fugene-6 (Promega) or Lipofectamine 2000 (ThermoFisher) with a 3:1 (μl:μg) ratio between the transfection reagent and plasmid DNA (∽0-5-1 μg per well in 24-well plates). Alternatively, transfection was performed in non-adherent cells in suspension immediately before plating^150^ using the same reagents. Two-three days after transfection, the cell cultures were either used for live-cell imaging (FRAP, optoDroplet induction) or fixed. Before fixation, cell cultures on coverslips were rinsed with phosphate-buffered saline (PBS, pH 7.4), fixed with 4% paraformaldehyde (ThermoFisher) in PBS for 15 min at room temperature (RT), rinsed with PBS (3X) and mounted on slides using the Dako fluorescent mounting medium (Agilent).

#### Immunocytochemistry

HEK293 and Neuro-2a cells, fixed 24–48 h after plating, were permeabilized (0.1% Triton X- in PBS, 15 min), rinsed in PBS (3X), and incubated in blocking solution (2% bovine serum albumin (BSA; Sigma) in PBS (1 h at RT). After rinsing in PBS (3X), the cells were incubated with a primary anti-FOXP2 antibody (ThermoFisher, #720031; 1:100 in 0.1% BSA in PBS) for 1 h at RT, rinsed in PBS (3X), and then incubated with fluorescently labeled (Alexa 488) anti-rabbit secondary antibodies for 1 h at RT. After rinsing in PBS (3X), coverslips were mounted on slides. In some experiments, coverslips were also incubated with DAPI (Sigma) for 15 min at room temperature and then rinsed with PBS (3X) before mounting.

#### Confocal fluorescence imaging and fluorescence recovery after photo bleaching (FRAP)

Confocal fluorescence imaging was performed using Olympus FV300 or Leica TCS SP5 confocal microscopes. Super-resolution imaging analyses were performed using a ZEISS LSM 800 Airyscan microscope system using default Airyscan settings. In general, each experiment was replicated at least three times and images were collected from multiple coverslips per experiment.

FRAP experiments^151^ were performed using the TCS SP5 microscope, and the FRAP Wizard module of the LAS-X software (Leica). Fluorescent foci formed by GFP-tagged FOXP2 polyQ variants in HEK293 cell nuclei (48–72 h after transfection) were brought into the focal plane, laser power and acquisition parameters were adjusted to avoid signal saturation, and one of the foci was included in a region of interest (ROI) to be photobleached. After acquiring three pre-bleaching images of the cell nucleus, one per second, the ROI was photobleached using the 488 nm laser (100% power) for 3 s, and fluorescence recovery was monitored by acquiring images of the nucleus every second for 120 s. After image acquisition, fluorescence levels in both photobleached and 2–3 control areas of each nucleus were quantified using the LAS-X software and normalized to their respective pre-bleaching fluorescence levels (i.e., the mean fluorescence level in the 3 pre-bleaching scans). To correct for slow photobleaching upon repeated laser scanning during the experiment, the values of the photobleached area were further normalized, at each time point, to the mean value of the non-photobleached control areas. In some experiments, the mobility of foci and nuclei on the *z* axis caused major distortions of the typical fluorescence recovery profiles and/or sudden disappearance of the foci from the ROI. These measurements, for both the *Hs* and *Rf* constructs, were not included in the analysis. Data were analyzed using *Easy-FRAP*^146^ for curve fitting, and *t*-half and immobile fraction calculations.

#### Liquid-liquid phase separation (LLPS) analysis

The spontaneous formation of condensates by fluorescently tagged human FOXP2, its fragments, and polyQ variants, was quantified by confocal fluorescence imaging in HEK293 cells 48–72 h after transfection. Maximum intensity projections of z stack fluorescence confocal images of microscopy fields (233x233, or 350 × 350 μm) were converted into 8-bit images using ImageJ (NIH). Image brightness and contrast were adjusted to highlight either the foci or the entire nuclei/cell profiles of the fluorescent cells. These images were used to automatically quantify, for each field, the surface area occupied by condensates and that occupied by nuclei/cells using *ad hoc* CellProfiler^147^ pipelines. The ratio between the two areas defined the proportion of the cell area occupied by condensates.

To define the sensitivity to 1,6-hexanediol (1,6-HEX) of the FOXP2-GFP and FOXP2-NTD-GFP foci, which is typical of LLPS-driven condensates,^103^ we used the protocol by Ulianov et al.^105^ Briefly, HEK293 cell cultures (48 h after transfection) were transiently permeabilized with 1% Tween 20 and exposed to 5% 1,6-HEX for 10 min. Control cultures were treated in the same manner, except that 1,6-HEX was omitted. The cells were then rinsed with PBS and fixed in 4% PFA in PBS (15 min at RT). The coverslips were mounted onto microscope slides and, after confocal imaging, the area occupied by foci was measured using CellProfiler, as described above.

The triggered induction of LLPS of protein fragments of interest was obtained using the optoDroplet system.^101^ Briefly, cells were transfected with plasmids suitable for the expression of FOXP2 fragments of interest fused to mCh-Cry2, as described above, and LLPS was induced 48–72 h after transfection by 488 nm light illumination using two distinct protocols.

To quantify the overall LLPS induction in cell populations, the cell cultures were photoactivated 48–72 h after transfection using blue light (488 nm), illuminating the entire coverslip, emitted by the 100 W fluorescent lamp of an Eclipse TE200 microscope (Nikon) with suitable dichroic filters for 5–7 min. Two minutes after photoactivation, the cells were rinsed in PBS and fixed for subsequent fluorescence confocal imaging to detect the mCherry red fluorescence. Control cells were treated in the same way, except that photoactivation was omitted. LLPS-driven condensate formation was quantified, as described above, by calculating for each construct the relative area of nuclei (for full-length FOXP2 or its polyQ variants), or cells (for FOXP2 NTD_CC_ fragments), occupied by condensates in photoactivated cultures comparison with non-photoactivated control cultures.

To quantify the kinetics of LLPS induction, reversibility, and re-induction, we performed live-cell imaging experiments using an Olympus FV300 confocal microscope. In these experiments, the culture medium was replaced with HBSS, pH 7.4 (Sigma) at RT and the red mCh fluorescence of 2–10 cells within a magnified confocal microscopy field (at an intermediate *z* level of the cell layer) was monitored using 561 nm laser light, every minute before (5 scans) and after (20 scans) a 60 s photoactivating pulse delivered by fast-scanning the sample with blue light (488 nm; 20% laser power). In some experiments, to test LLPS reinduction, a similar pulse was delivered 20 min after the first one and red fluorescence was monitored again for 20 min. LLPS-driven condensate formation was quantified, as described above, by calculating the relative area of nuclei (for full-length FOXP2 or its polyQ variants), or cells (for FOXP2 N-terminal fragments), occupied by condensates in cultures before and after photoactivation. For each nucleus/cell, all values were normalized to the mean value in the scans before the first photoactivating pulse.

#### Luciferase transcription reporter assay

For luciferase transcription reporter assays, HEK293 cells were plated into 96-well white culture plates (VWR). Twenty-four hours after plating, the cells were co-transfected with three plasmid vectors, i.e., (*i*) the pcDNA4/HisMax plasmid, either empty (control condition) or encoding human FOXP2 (*Hs*), or one of its polyQ variants (*Cs*, *Ms*, *Rf*, and *ΔQ*_*1*_*Q*_*2*_); (*ii*) the pGL3-SPRX2 plasmid^106^; (*iii*) the pRL-CMV vector (Promega) as a transfection control. For transfecting 8 culture wells, we mixed 1125 μg of the empty pcDNA4/HisMax plasmid (or equimolar amounts of the same vector encoding human FOXP2 or its polyQ variants), 255 μg of pGL3-SPRX2, and 120 μg of pRL-CMV. The DNA mix was transfected in a 3:1 μg:μl ratio with Fugene-6 or Lipofectamine 2000, following the manufacturers’ protocols. Forty-eight hours after transfection, firefly and *Renilla* luciferase activity were sequentially measured using the Dual-Glo luciferase assay system (e2929; Promega) and a GloMax microplate reader (Promega). For each well, the firefly luciferase luminescence was first normalized against the *Renilla* luciferase luminescence to correct for transfection efficiency. Then, all luminescence values were further normalized to the average value measured in the experimental control group expressing the empty pcDNA4/HisMax plasmid. In some luciferase assays, GFP-tagged FOXP2 variants were expressed and the pEGFP-C1 vector, instead of pcDNA4/HisMax, was transfected in control cultures. At least 18 wells from three independent experiments were analyzed for each FOXP2 variant construct. Each experiment included control wells for normalization (pcDNA4/HisMax control), for a total of 116 control wells across all the luciferase assay experiments.

#### Software

Basicranium μ-CT stacks shown in Figure 1C were obtained using the Aleph 3D-viewer embedded in the MorphoSource website (www.morphosource.org). *Ochotona princeps* vocalization recordings were analyzed using Kaleidoscope Lite 5.4.7 software (Wildlife Acoustics). Digital endocasts of the left cochlea and lateral semicircular canals, as shown in Figures 2E and 2F, and landmark coordinates were obtained using ITK-SNAP 3.8.0.^142^ Gwyddion 2.57^145^ was used to analyze AFM experiments. UCSF Chimera^144^ was used to analyze, and generate images of protein structure models generated by Raptor-X.^143^ Circular dichroism data were analyzed using Spectra Manager (version 2.06.01; JASCO Corporation) and Excel (Microsoft). Confocal fluorescence microscopy images were visualized and processed using ImageJ (NIH), Fluoview (Olympus), LAS-X (Leica) and ZEN lite (Zeiss). ImageJ and Photoshop Elements 11 (Adobe) were used for image processing and to generate figures.

### Quantification and statistical analysis

Data are expressed as mean ± standard error of mean (SEM). The details concerning the specific parameters that were quantified in each analysis (e.g., polyQ lengths, vocalization-related and morphological parameters, condensate area, luminescence), the number of experimental groups, their composition (e.g., species, cells, condensates, culture wells) and numerosity (*n*), are reported above, where appropriate, in the results and method details sections. Student’s *t* test, one- or two-way ANOVA, ANOVA for repeated measures, Newman-Keuls *post hoc* test. and other tests, were performed where appropriate, as detailed in the results section. In all instances, a p value ≤ 0.05 was considered statistically significant. Data analysis and statistics were performed using Excel (Microsoft), Statistica (Tibco) and Python 3 (*pandas*, *matplotlib*, and *numpy* packages).

#### Accession numbers

The accession numbers of protein or nucleotide (genomic) sequences of the FOXP2 and FOXP1 orthologs that were analyzed are reported in Table S1. Micro computed tomography (*μ*CT) scans were obtained from the MorphoSource database (https://www.morphosource.org) The scan Media IDs (also listed in Table S4) are: 86463,96992,2150,170220,170399,170595,165459,02723, 158814,13131,11011,10956,168375,15460,15477,15612,15720,410414,394893,140436,8620,114319,56554,22387,16145,16139,16131,13626,22292,22235,21964,21967,21967,21952,45544,167374,55626,55624,47005,167378,55698,79025,367903,47052,55618,55349,167384,365356,167937,36587,57664,167934,36439,36171,25920,25943,36828,167937,36257,36434,36511,36128,57668,S9128; The *P. alecto* scan S9128 was used only to generate Figure 1C, *left panel*).

## Data Availability

• The analyses presented in Figures 1, 2, 3, and S1–S4 are based on *i*) publicly available protein and nucleotide sequences, obtained from the NCBI (www.ncbi.nlm.nih.gov) and Uniprot (www.uniprot.org) databases, *ii*) animal vocalization frequency and body mass datasets as derived from the cited literature, and *iii*) μCT cranial scans, obtained from the Morphosource database (www.morphosource.org), either as freely accessible data or upon authorization from dataset administrators. The accession numbers of FOXP2 and FOXP1 protein or nucleotide sequences are listed in Table S1*.* The vocalization frequency and body mass datasets were derived from the literature, as listed (with PMID identifiers) in Tables S2 and S3*.* The MorphoSource specimen list is reported in Table S4*.* The data related to structural analyses, microscopy and other experiments reported in this paper will be shared by the lead contact upon request. • This paper does not report original code. • Any additional information required to re-analyze the data reported in this paper is available from the lead contact upon request.

## Supplemental information

Document S1. Figures S1–S7

Table S1. FOXP2 and FOXP1 protein and genomic sequence IDs, related to Figures 1, 2, and 3

Table S2. Sources of vocalization frequency parameters, related to Figures 1, 2, and 3

Table S3. Sources of body parameters, related to Figures 1, 2, and 3

Table S4. MorphoSource specimen IDs, related to Figures 1 and 2

Table S5. PhyloPic silhouette IDs, related to Figures 1, 2, and 3
