# Supplementary material for: PolyQ length-based molecular encoding of vocalization frequency in FOXP2
Source: iScience. 2023 Sep 27;26(10):108036. doi: 10.1016/j.isci.2023.108036 (PMC10582585; doi:10.1016/j.isci.2023.108036)
Supplement: Document S1. Figures S1–S7 [file mmc1.pdf]

iScience, Volume *26*

Supplemental information

PolyQ length-based molecular encoding

# of vocalization frequency in FOXP2

Serena Vaglietti, Veronica Villeri, Marco Dell'Oca, Chiara Marchetti, Federico Cesano, Francesca Rizzo, Dave Miller, Louis LaPierre, Ilaria Pelassa, Francisco J. Monje, Luca Colnaghi, Mirella Ghirardi, and Ferdinando Fiumara

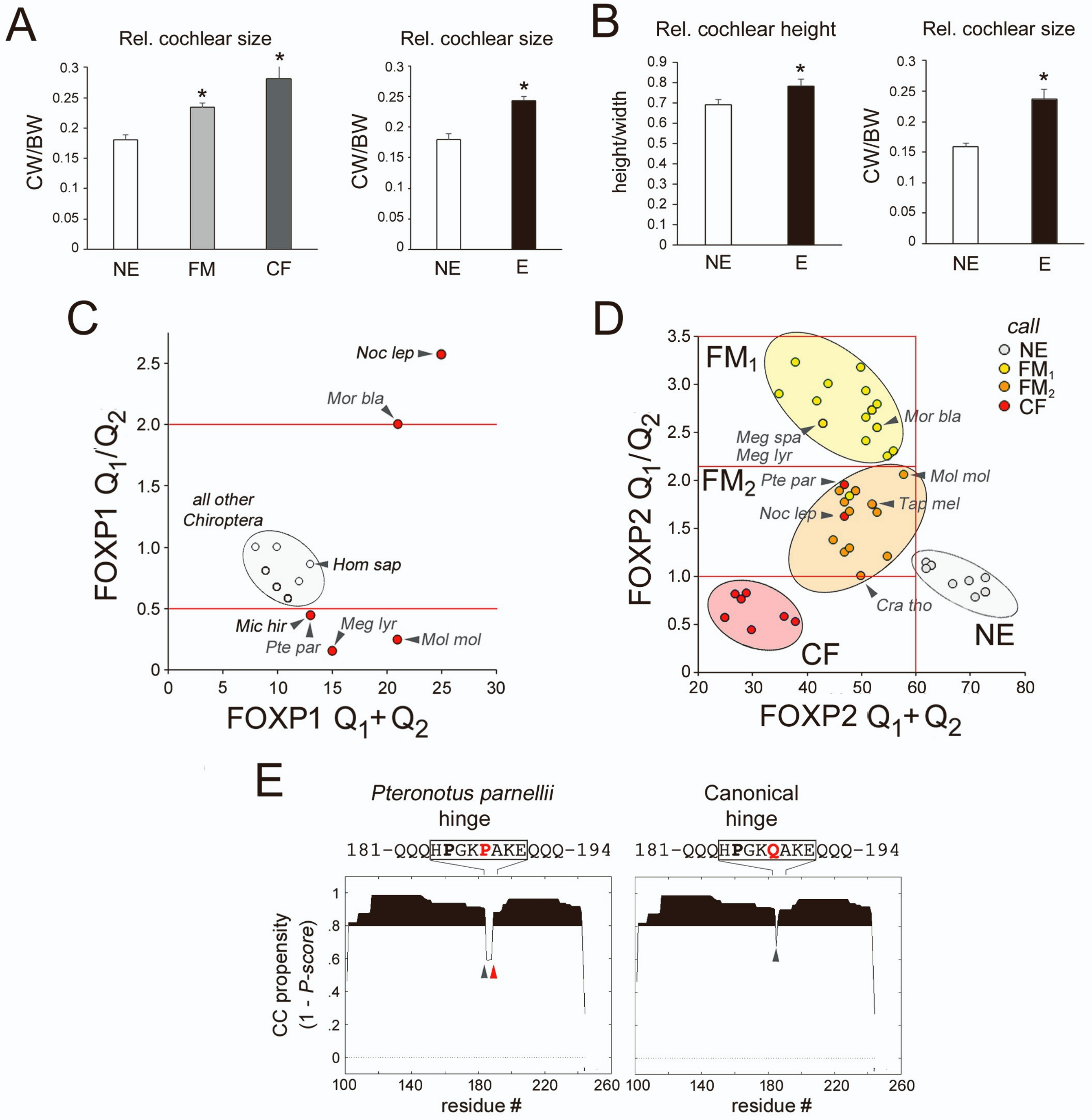
A
Rel. cochlear size
CW/BW
NE
FM
CF
Rel. cochlear size
CW/BW
NE
E
B
Rel. cochlear height
height/width
NE
E
Rel. cochlear size
CW/BW
NE
E
C
FOXP1 Q1/Q2
FOXP1 Q1+Q2
Noc lep
Mor bla
all other Chiroptera
Hom sap
Mic hir
Pte par
Meg lyr
Mol mol
D
FOXP2 Q1/Q2
FOXP2 Q1+Q2
call
NE
FM1
FM2
CF
Mor bla
Meg spa
Meg lyr
Pte par
Mol mol
Tap mel
Noc lep
Cra tho
E
Pteronotus parnellii hinge
181-QQQHPGKPAKEQQQ-194
Canonical hinge
181-QQQHPGKQAKEQQQ-194
CC propensity (1 - P-score)
residue #

Figure S1

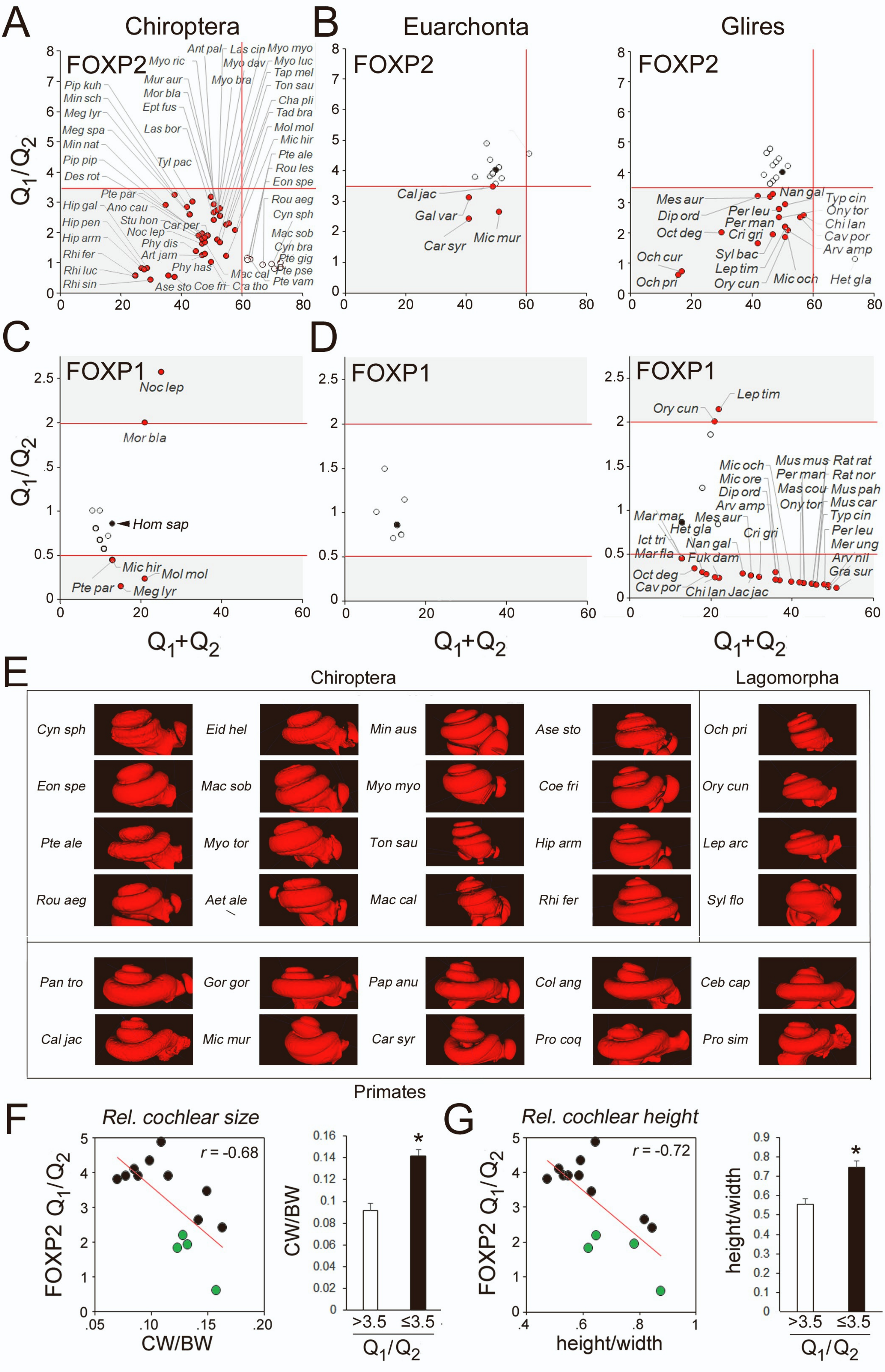
A
Chiroptera
FOXP2
Myo ric
Ant pal
Las cin
Myo myo
Myo dav
Myo luc
Pip kuh
Mur aur
Myo bra
Tap mel
Min sch
Mor bla
Ton sau
Meg lyr
Ept fus
Cha pli
Tad bra
Meg spa
Las bor
Mol mol
Min nat
Mic hir
Pip pip
Tyl pac
Pte ale
Rou les
Des rot
Eon spe
Pte par
Rou aeg
Hip gal
Ano cau
Cyn sph
Hip pen
Stu hon
Car per
Noc lep
Mac sob
Hip arm
Phy dis
Cyn bra
Rhi fer
Art jam
Pte gig
Phy has
Mac cal
Pte pse
Rhi luc
Pte vam
Rhi sin
Ase sto
Coe fri
Cra tho
B
Euarchonta
FOXP2
Cal jac
Gal var
Car syr
Mic mur
Glires
FOXP2
Mes aur
Nan gal
Typ cin
Dip ord
Per leu
Ony tor
Per man
Chi lan
Oct deg
Cri gri
Cav por
Arv amp
Och cur
Syl bac
Lep tim
Och pri
Ory cun
Mic och
Het gla
Q1/Q2
C
FOXP1
Noc lep
Mor bla
Hom sap
Mic hir
Mol mol
Pte par
Meg lyr
D
FOXP1
FOXP1
Lep tim
Ory cun
Mic och
Mus mus
Rat rat
Mic ore
Per man
Rat nor
Dip ord
Mas cou
Mus pah
Arv amp
Ony tor
Mus car
Mar mar
Mes aur
Typ cin
Het gla
Per leu
Ict tri
Cri gri
Mer ung
Nan gal
Mar fla
Fuk dam
Arv nil
Oct deg
Gra sur
Cav por
Chi lan
Jac jac
Q1+Q2
E
Chiroptera
Lagomorpha
Cyn sph
Eid hel
Min aus
Ase sto
Och pri
Eon spe
Mac sob
Myo myo
Coe fri
Ory cun
Pte ale
Myo tor
Ton sau
Hip arm
Lep arc
Rou aeg
Aet ale
Mac cal
Rhi fer
Syl flo
Pan tro
Gor gor
Pap anu
Col ang
Ceb cap
Cal jac
Mic mur
Car syr
Pro coq
Pro sim
Primates
F
Rel. cochlear size
r = -0.68
FOXP2 Q1/Q2
CW/BW
>3.5
≤3.5
Q1/Q2
G
Rel. cochlear height
r = -0.72
FOXP2 Q1/Q2
height/width
>3.5
≤3.5
Q1/Q2

Figure S2

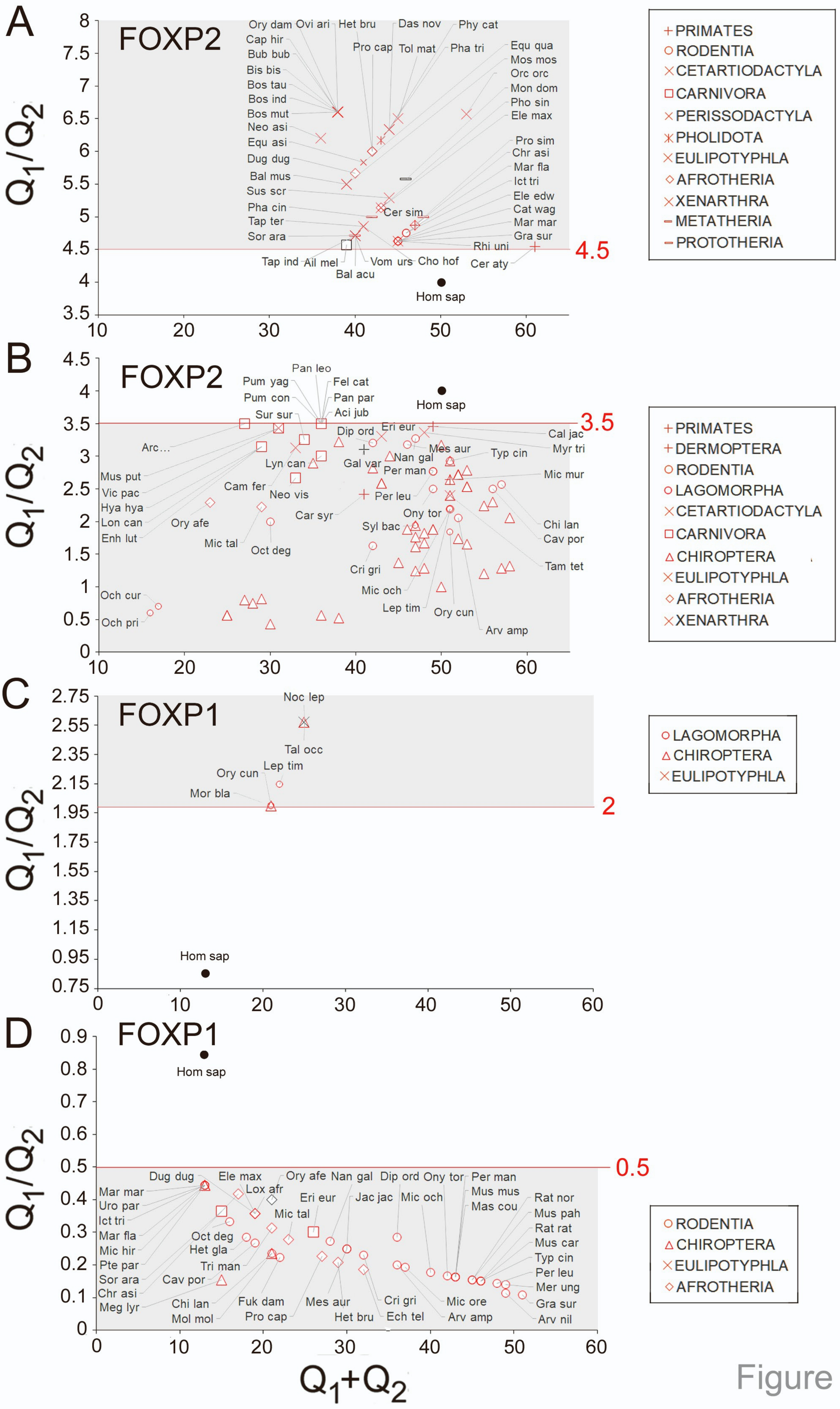
A
FOXP2
B
FOXP2
C
FOXP1
D
FOXP1
Q_1/Q_2
Q_1+Q_2
4.5
3.5
2
0.5
Hom sap
Ory dam
Ovi ari
Het bru
Das nov
Phy cat
Cap hir
Bub bub
Bis bis
Bos tau
Bos ind
Bos mut
Neo asi
Equ asi
Dug dug
Bal mus
Sus scr
Pha cin
Tap ter
Sor ara
Tap ind
Ail mel
Bal acu
Vom urs
Cho hof
Cer aty
Rhi uni
Pro cap
Tol mat
Pha tri
Equ qua
Mos mos
Orc orc
Mon dom
Pho sin
Ele max
Pro sim
Chr asi
Mar fla
Ict tri
Ele edw
Cat wag
Mar mar
Gra sur
Cer sim
+PRIMATES
○RODENTIA
×CETARTIODACTYLA
□CARNIVORA
×PERISSODACTYLA
⁎PHOLIDOTA
×EULIPOTYPHLA
◇AFROTHERIA
×XENARTHRA
−METATHERIA
−PROTOTHERIA
Pan leo
Pum yag
Fel cat
Pum con
Pan par
Aci jub
Sur sur
Dip ord
Eri eur
Cal jac
Myr tri
Arc…
Mes aur
Typ cin
Lyn can
Gal var
Nan gal
Per man
Mic mur
Mus put
Vic pac
Hya hya
Lon can
Enh lut
Cam fer
Neo vis
Per leu
Ory afe
Car syr
Ony tor
Syl bac
Chi lan
Cav por
Mic tal
Oct deg
Cri gri
Tam tet
Mic och
Och cur
Och pri
Lep tim
Ory cun
Arv amp
+PRIMATES
+DERMOPTERA
○RODENTIA
○LAGOMORPHA
×CETARTIODACTYLA
□CARNIVORA
△CHIROPTERA
×EULIPOTYPHLA
◇AFROTHERIA
×XENARTHRA
Noc lep
Tal occ
Lep tim
Ory cun
Mor bla
○LAGOMORPHA
△CHIROPTERA
×EULIPOTYPHLA
Dug dug
Ele max
Ory afe
Nan gal
Dip ord
Ony tor
Per man
Mar mar
Lox afr
Mus mus
Uro par
Eri eur
Jac jac
Mic och
Mas cou
Rat nor
Ict tri
Mic tal
Mus pah
Rat rat
Mar fla
Oct deg
Mus car
Mic hir
Het gla
Typ cin
Pte par
Tri man
Per leu
Sor ara
Cav por
Mer ung
Chr asi
Gra sur
Meg lyr
Chi lan
Fuk dam
Mes aur
Cri gri
Mic ore
Mol mol
Pro cap
Het bru
Ech tel
Arv amp
Arv nil
○RODENTIA
△CHIROPTERA
×EULIPOTYPHLA
◇AFROTHERIA

Figure S3

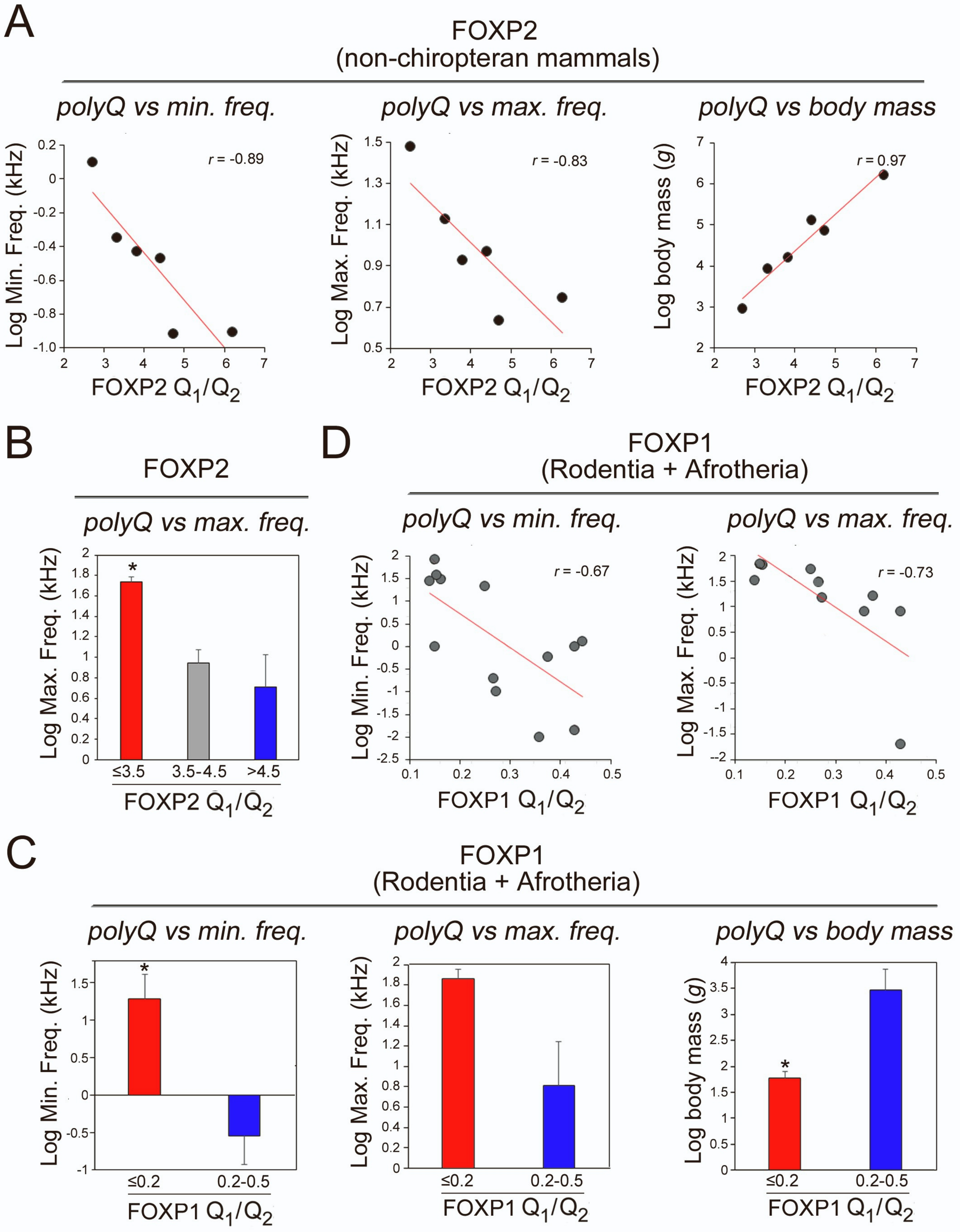
A
FOXP2
(non-chiropteran mammals)
polyQ vs min. freq.
r = -0.89
Log Min. Freq. (kHz)
FOXP2 Q1/Q2
polyQ vs max. freq.
r = -0.83
Log Max. Freq. (kHz)
FOXP2 Q1/Q2
polyQ vs body mass
r = 0.97
Log body mass (g)
FOXP2 Q1/Q2
B
FOXP2
polyQ vs max. freq.
Log Max. Freq. (kHz)
*
≤3.5
3.5-4.5
>4.5
FOXP2 Q1/Q2
D
FOXP1
(Rodentia + Afrotheria)
polyQ vs min. freq.
r = -0.67
Log Min. Freq. (kHz)
FOXP1 Q1/Q2
polyQ vs max. freq.
r = -0.73
Log Max. Freq. (kHz)
FOXP1 Q1/Q2
C
FOXP1
(Rodentia + Afrotheria)
polyQ vs min. freq.
Log Min. Freq. (kHz)
*
≤0.2
0.2-0.5
FOXP1 Q1/Q2
polyQ vs max. freq.
Log Max. Freq. (kHz)
≤0.2
0.2-0.5
FOXP1 Q1/Q2
polyQ vs body mass
Log body mass (g)
*
≤0.2
0.2-0.5
FOXP1 Q1/Q2

Figure S4

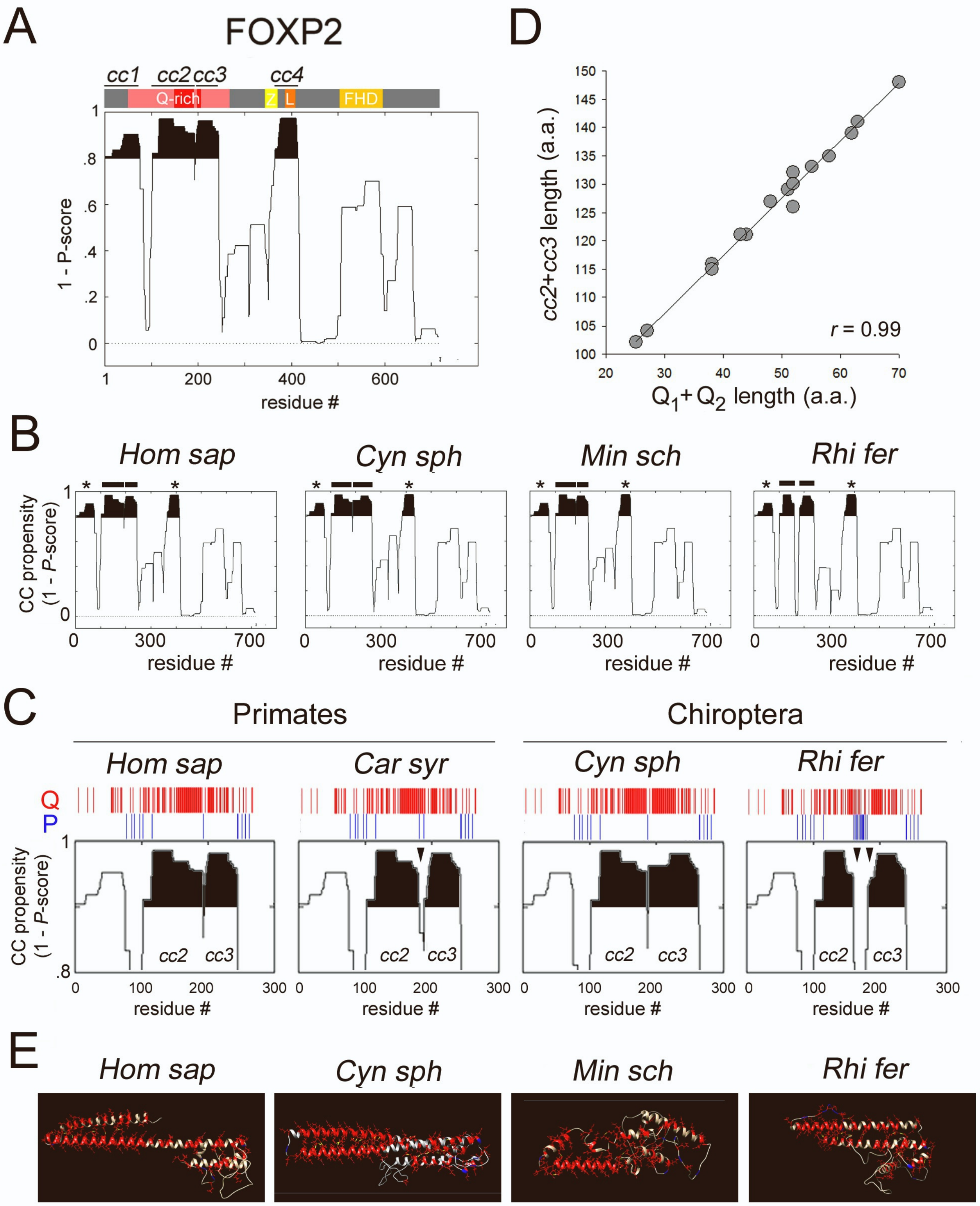
A
FOXP2
cc1
cc2 cc3
cc4
Q-rich
Z
L
FHD
1 - P-score
residue #
D
cc2+cc3 length (a.a.)
Q_1+Q_2 length (a.a.)
r = 0.99
B
Hom sap
Cyn sph
Min sch
Rhi fer
CC propensity (1 - P-score)
residue #
C
Primates
Chiroptera
Hom sap
Car syr
Cyn sph
Rhi fer
Q
P
CC propensity (1 - P-score)
cc2
cc3
residue #
E
Hom sap
Cyn sph
Min sch
Rhi fer

Figure S5

A

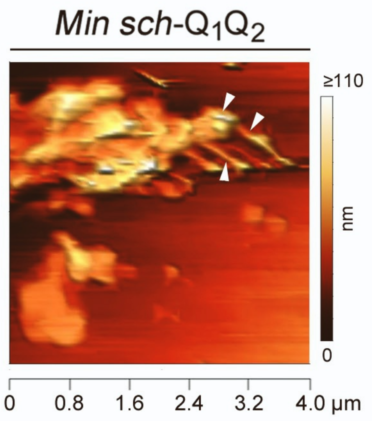

Figure S6

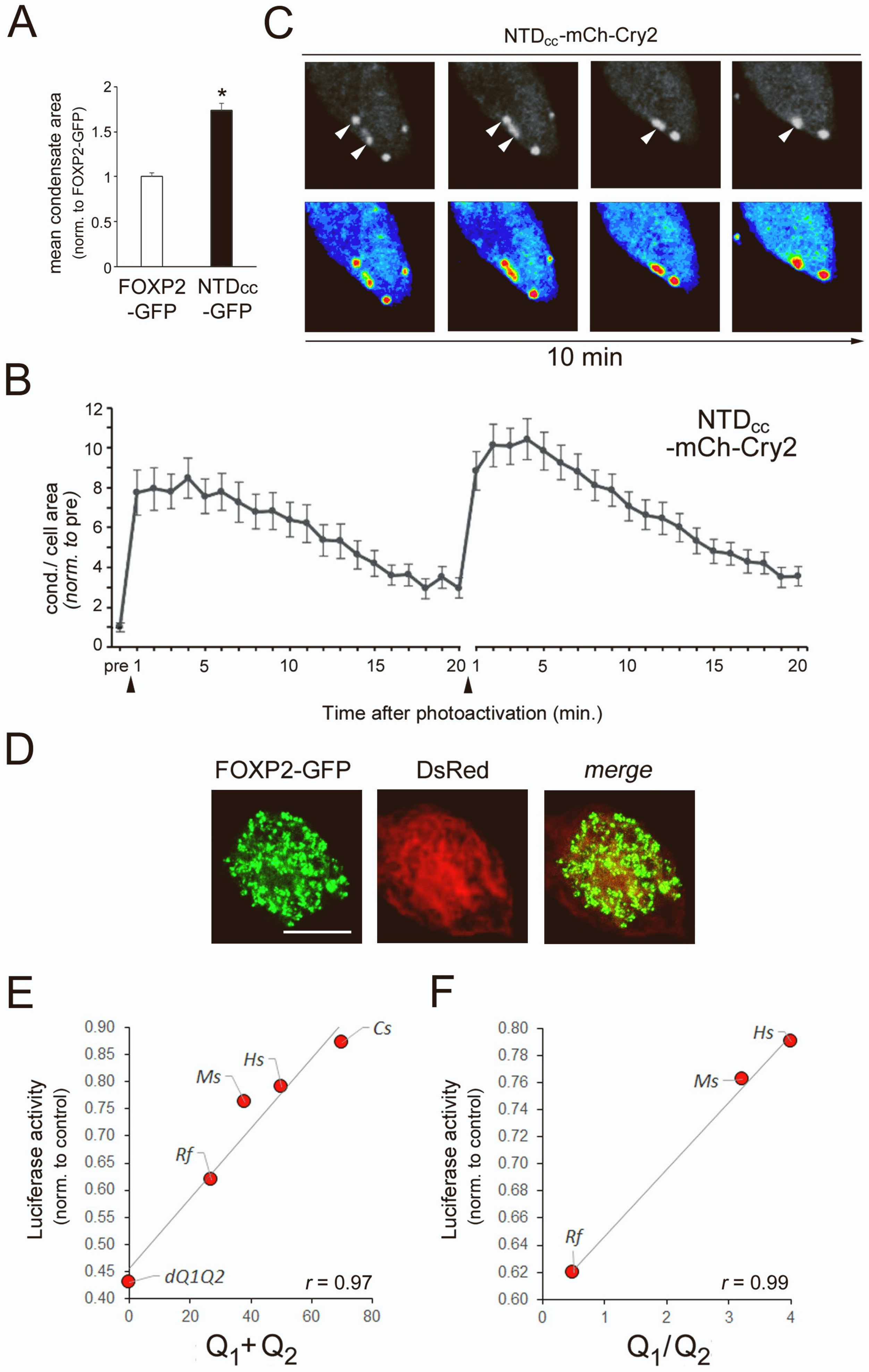
A
mean condensate area
(norm. to FOXP2-GFP)
*
FOXP2-GFP
NTDcc-GFP
C
NTDcc-mCh-Cry2
10 min
B
cond./ cell area
(norm. to pre)
NTDcc
-mCh-Cry2
pre
Time after photoactivation (min.)
D
FOXP2-GFP
DsRed
merge
E
Luciferase activity
(norm. to control)
Cs
Hs
Ms
Rf
dQ1Q2
r = 0.97
Q1+Q2
F
Hs
Ms
Rf
r = 0.99
Q1/Q2

Figure S7

SUPPLEMENTAL FIGURE LEGENDS

Figure S1 – Cochlear morphological parameters and aspects of FOXP2/1 polyQ variation in Chiroptera (*related to Fig. 1*).

A. *Left panel.* Bar graph displaying the mean relative cochlear size (CW/BW) as derived from [1] and [2], in NE (n = 7), FM (n = 27), CF (n = 6) bat species. A one-way ANOVA indicates an overall significant difference in CW/BW related to vocalization type ($F_{(2, 37)}$= 14.326, p<0.001). Both the FM and CF groups significantly differ from the NE group (p<0.01 in both instances, Newman-Keuls (NK) *post hoc* test). *Right panel.* A Student's *t*-test also indicates a significant difference (p<0.01) in CW/BW between bats that do not use laryngeal echolocation (NE) and those that use it (E; i.e. FM+CF) bats. Data are represented as mean +/- SEM. **B.** *Left panel.* Bar graphs displaying the mean relative cochlear height (height/width ratio; as in [3]; as derived from the analysis of cochlear endocasts that we generated from available µCT scans (Morphosource.org) in a sample set of E (n = 8) and NE (n = 8) bats (see **Suppl. Table 4** for the species and scan ID lists). The relative cochlear height is significantly higher in the E group (0.68 ± 0.02 *vs* 0.78 ± 0.03, n = 8 in both groups, p < 0.05, *t*-test). The set of species that were analyzed in measuring relative cochlear height is representative of the larger dataset studies in *panel A*, as shown by the significant difference between the two groups in terms of CW/BW (0.15 ± 0.005 (NE) *vs* 0.23 ± 0.01, n = 8 in both groups, p < 0.001, *t*-test; *right panel*), similar to what shown in *panel A*. Data are represented as mean +/- SEM. **C.** FOXP1 polyQ sum-*versus*-ratio plot for the available chiropteran sequences. Species are represented by *circles*. Note how, at variance with what found for FOXP2 (*Fig. 1E-F*), most chiropteran species (*white circles*) display sum and ratio values similar to those of *Homo sapiens* (*Hom sap*) and group into a single cluster (*oval*) irrespective of their call type (NE, FM, CF). Strikingly, however, 5 of the 8 species known to converge phenotypically in terms of call type with species of other superfamilies (as indicated in *Fig. 1B,E-F*), and for which the FOXP1 sequence is available, have Q_1 and Q_2 lengths that deviate from the human-like ones. In these species, the Q_1/Q_2 ratio becomes either ≤0.5 (*lower red line*; mostly by Q_2 elongation, as in *Pternotus parnellii*, *Megaderma lyra*, *Molossus molossus*) or ≥2 (*upper red line*; mostly by Q_1 elongation, as in *Noctilio leporinus*, *Mormoops blainvillei*). Note how the FOXP1 sequence is not available, and could not be identified using genome BLAST searches, for the other three of those eight species. Overall, these observations indicate that, in Chiroptera, USV type-related polyQ length variation is largely prevalent in FOXP2 (as shown in *Fig. 1E-F*), but can occur for a few species also, or exclusively (*P. parnellii*, *N. leporinus*), in FOXP1. Thus, such changes in FOXP1 polyQ repeats appear to be an alternative route of molecular change in a few species which diverged from their closest phylogenetic relatives in terms of USV type. Given the limited number of these species it is difficult in Chiroptera (but see below for other taxa) to define quantitatively the relation between FOXP1 polyQ lengths and vocalization parameters. **D.** As in *Fig. 1F* with threshold values of Q_1+Q_2 and Q_1/Q_2 used to define the NE, FM_1, FM_2, and CF clusters, as reported in the *Results* section, are highlighted by *red lines*. **E.** *Left panel*. Coiled-coil propensity prediction for the Q_1-Q_2 region (residues 100-244) of the FOXP2 N-terminal domain of *Pteronotus parnellii*, as obtained using Paircoil2 ([4]; propensity expressed as 1 – *Pscore* as in [5]). The predicted protein sequence of *P. parnellii* FOXP2 was derived from a BLAST search of the available genome using the human FOXP2 sequence (GenBank locus: JAIWKQ010000127.1). Peaks of high CC propensity (0.8-1) are highlighted in *black*. The primary sequence of the 8-residue hinge peptide ('HPGKPAKE') separating Q_1 and Q_2 is shown above the prediction. The first proline residue, common to all other chiropteran FOXP2 sequences is highlighted in *black*. The second proline residue, unique to *P. parnellii*, is highlighted in *red*. Note how the hinge peptide bearing the two helix-breaking proline residues corresponds to a drop of CC propensity between two major peaks (*double arrowhead*). *Right panel.* If the second proline residue is replaced by a glutamine one (in *red*), as found in all the other chiropteran hinge peptides, the gap between the two peaks is minimal. Thus, the presence of a longer gap between the two peaks, as a result of the presence of the two proline residues, can create a longer loop between the CC-prone upstream and downstream helices. Interestingly (see *Fig. 4* and *Fig. S4C* below), a similar proline-rich loop between the Q_1 and Q_2 α-helices is found in FOXP2 orthologs of bats emitting CF USVs, and *P. parnellii* functionally converges with these species in emitting the same type of USVs. Thus, this modification of FOXP2, together with that of FOXP1 (see *panel C*), may together represent molecular convergence correlates of phenotypic convergence in vocalization type (CF) between *P. parnellii* (Yangochiroptera) and Rhinolophoidea (Yinpterochiroptera).

Figure S2 –FOXP2/1 polyQ variation and cochlear morphology in Chiroptera and Euarchontoglires (*related to Fig. 2*).

A-B. PolyQ sum-*vs*-ratio plots for FOXP2 orthologs of species belonging Euarchontoglires (*panel B*) in comparison with Chiroptera (*panel A*). The position of the human ortholog (*Hom sap*) in *panel B* graphs is marked by a *black* circle. In *panel A, upper graph,* USV-emitting chiropteran species (in *red)* occupy an area of the graph

(in *gray*, delimited by *red* lines) with FOXP2 polyQ sum <60 and ratio <3.5. The same area is highlighted in the FOXP2 plots in *panel B*. The indicated species of Euarchontoglires falling into this area, and thus converging with USV-emitting bats in terms of FOXP2 polyQ length, are highlighted in *red*. Notably, for most of these species, there is also evidence in the literature of phenotypic convergence with bats in terms of ultrasonic vocalization/hearing (see *Supplemental References*). Four species of Euarchonta, among those that were analyzed, fall into this area and there is evidence for all of them of ultrasonic vocalization/hearing (see [6] for *Carlito syrichta* (*Car syr*), [7] for *Callithrix jacchus* (*Cal jac*), [8] for *Microcebus murinus* (*Mic mur*), and [9] for *Galeopterus variegatus* (*Gal var*). Eighteen of the Glires species that were analyzed, i.e. 13 (over 30) species belonging to Rodentia and all five species belonging to Lagomorpha, fall into the same area (Q1/Q2 ratio ≤3.5) and there is evidence for 17 of them, or other closely related species belonging to the same genus, of ultrasonic vocalization/hearing (see [10] for *Peromyscus maniculatus* (*Per man*) and the *Peromyscus* genus (which includeas *Peromyscus leucopus* (*Per leu*) in our dataset), [11] for *Onychomys torridus* (*Ony tor*), [12] *Microtus ochrogaster* (*Mic och*), [13] for Arvicolinae (which include *Arvicola amphibius* (*Arv amp*) in our dataset), [14], [15] and [16] for *Mesocricetus auratus* (*Mes aur*), [14] for *Cricetulus griseus* (*Cri gri*), [16] for *Typhlomys cinereus* (*Typ cin*), [17] and [18] for the *Dipodomys* genus (which includes *Dipodomys ordii* (*Dip ord*) in our dataset), [19] for *Octodon degus* (*Oct deg*), [17] for *Chinchilla lanigera* (*Chi lan*), [20],[17], and [21] for *Cavia porcellus* (*Cav por*), the *Results* section for *Ochotona princeps* (*Och pri*), [22] for the *Ochotona* genus (which includes *Ochotona curzoniae* (*Och cur*), in our dataset), [23] for *Oryctolagus cuniculus* (*Ory cun*), [24] for the *Sylvilagus* and *Lepus* genera (which include, respectively, *Sylvilagus bachmani* (*Syl bac*) and *Lepus timidus* (*Lep tim*), in our dataset). The only exception in these 18 species was *Nannospalax galili*, a fossorial species for which there is evidence of low- rather than high-frequency hearing sensitivity [25], which has a ratio ≤3.5, although the highest (0.27) found among the 13 species of rodents. Notably, another subterranean rodent with degenerate high-frequency hearing, i.e. *Heterocephalus glaber,* also has a Q_1/Q_2 ratio below 3.5 but, unique case among non-chiropteran mammals, also has a Q_1+Q_2 sum >60, thus converging with bats devoid of laryngeal echolocation (NE bats; see *Results*), rather than USV-emitting bats. Thus, the two subterranean rodents lie near the edges or outside the graph area occupied by USV-emitting bats. **C-D.** As in *panels A-B*, for FOXP1. *Panel C* reproduces *Fig. S1C* here for comparison here with Euarchontoglires. *Panel D* shows, for Euarchonta, how all species have human-like polyQ repeats in FOXP1, as most chiropteran species. No species converges with the five USV-emitting bat species, known to have varied their USV type with respect to their closest phylogenetic relatives, in having a Q_1/Q_2 ≤0.5 or ≥2. Notably, however, all the Rodentia species converge with three of these USV-emitting bats in having a Q_1/Q_2 ≤0.5. Overall, this correlates well with the generalized occurrence of USV-vocalization/hearing in Rodentia (e.g. [26]), except for a few species of subterranean rodents. Indeed, there is evidence of ultrasonic vocalization/hearing for 25 of the 30 species that were analyzed, (see [21] for *Rattus rattus* (*Rat rat*), [17] for *Rattus norvegicus* (*Rat nor*), [12] for *Mastomys coucha* (*Mas cou*), [17] and [21] for *Mus musculus* (*Mus mus*), and for the *Mus* genus (which includes *Mus pahari* (*Mus pah*) and *Mus caroli* (*Mus car*) in our dataset), [17] for *Arvicanthis niloticus* (*Arv nil*), [17], [15], [27], and [18] for *Meriones unguiculatus* (*Mer ung*), [28] for *Microtus oregoni* (*Mic ore*), [17] for the *Marmota* genus (which includes *Marmota marmota* (*Mar mar*) and *Marmota flaviventris* (*Mar fla*) in our dataset), [29] for the *Spermophilus* genus (which includes *Ictidomys tridecemlineatus* (*Ict tri*) in our dataset), [30] for the *Urocitellus* genus (which includes *Urocitellus parryii* (*Uro par*) in our dataset); for the other 13 of these species, references are listed above for *panel B*). For two species, i.e. *Grammomys surdaster* (*Gra sur*) and *Jaculus jaculus* (*Jac jac*) we did not find specific information on ultrasonic vocalization/hearing in the literature. In the other three rodent species that were analyzed, i.e. *Heterocephalus glaber*, *Fukomys damarensis*, and *Nannospalax galili*, which adapted to the subterranean environment with loss of high-frequency hearing ([27], [19], [25]), the FOXP1 Q_1/Q_2 ratio is in the range of other USV-emitting species. However, as indicated above (*panel B*), the FOXP2 polyQ repeats fall outside, or at the edges, of the sum-*vs*-ratio plot area occupied by USV-emitting bats (see *panel B*), consistent with their loss of high-frequency sensitivity. Thus, for these species, changes in the polyQ repeats of FOXP2, rather than FOXP1, appear to be associated with their phenotypic divergence in frequency range from other rodents. Notably, besides a quite generalized ability to emit and hear USVs, Rodentia can also have, at the same time, considerable auditory sensitivity to low frequencies with bimodal audiograms [17], in relation to their adaptation to semi-fossorial/fossorial or desertic environments and the ability to perceive seismic signals. Indeed, Heffner and Heffner [17] distinguished "high-" and "low-frequency" rodents. Species in both groups can emit/hear USVs, but the latter group has in addition enhanced low-frequency sensitivity. Strikingly, species belonging to the high-frequency group tend to have lower FOXP1 Q_1/Q_2 ratio (<0.2) than those in the low-frequency group. Indeed, species with a ratio <0.2 have significantly higher frequency hearing thresholds than those with a ratio between 0.2 and 0.5 (see *Fig. S4B-C*). **E.** Representative cochlear endocasts (images not to scale) of species/genera of interest belonging to Primates (n=10), Lagomorpha (n=4), and Chiroptera (n=16) generated from µCT scans available in the Morphosource database (see *Suppl. Table 4* for the scan IDs). The species are: for Primates, *Pan troglodytes* (*Pan tro*), *Gorilla gorilla* (*Gor gor*), *Papio anubis* (*Pap anu*), *Colobus angolensis* (*Col ang*), *Cebus capucinus* (*Ceb cap*), *Callithrix jacchus* (*Cal jac*), *Microcebus murinus* (*Mic mus*), *Carlito syrichta* (*Car syr*), *Propithecus coquereli* (*Pro coq*), *Prolemur*

simus (*Pro sim*), for Lagomorpha, *Ochotona princeps* (*Och pri*), *Oryctolagus cuniculus* (*Ory cun*), *Lepus arcticus* (*Lep arc*), *Sylvilagus floridanus* (*Syl flo*), and, for Chiroptera, *Cynopterus sphinx* (*Cyn sph*), *Eonycteris spelaea* (*Eon spe*), *Pteropus alecto* (*Pte ale*), *Rousettus aegyptiacus,* (*Rou aeg*), *Eidolon helvum* (*Eid hel*), *Macroglossus sobrinus* (*Mac sob*), *Myonycteris torquata* (*Myo tor*), *Aethalops alecto* (*Aet ale*), *Miniopterus australis* (*Min sch*), *Myotis myotis* (*Myo myo*), *Tonatia saurophila* (*Ton sau*), *Macrotus californicus* (*Mac cal*), *Aselliscus stoliczkanus* (*Ase sto*), *Coelops frithii* (*Coe fri*), *Hipposideros armiger* (*Hip arm*), *Rhinolophus ferrumequinum* (*Rhi fer*). **F.** *Left panel*. Scatterplot displaying the correlation between FOXP2 Q_1/Q_2 ratio and relative cochlear size (CW/BW; r = -0.68, n = 14, p<0.01) of species belonging to Primates and Lagomorpha. *Right panel*. Bar graphs displaying the significant difference (p<0.001, *t*-test) in mean CW/BW between species with FOXP2 Q_1/Q_2 ratio ≤3.5 (0.14 ± 0.005; n = 7) or >3.5 (0.09 ± 0.006; n = 7). Data are represented as mean +/- SEM. **G.** As in *panel D,* but for the cochlear height/width ratio. Scatterplot displaying the correlation between FOXP2 Q_1/Q_2 ratio and relative cochlear height (height/width; *right panel;* r = -0.72, n = 14, p<0.01) of species belonging to Primates and Lagomorpha. *Right panel*. Bar graphs displaying the significant difference (p<0.002, *t*-test) in mean height/width ratio between species with FOXP2 Q_1/Q_2 ratio ≤3.5 (0.74 ± 0.04; n = 7) or >3.5 (0.55 ± 0.02; n = 7).

Figure S3 – Regions of interest of the FOXP2/1 polyQ sum-*vs*-ratio scatterplots in mammals (*related to Fig. 3*).

A-B. Details of regions of interest of the FOXP2 polyQ sum-*vs*-ratio plots for all the mammalian species that were analyzed. The (super)order to which each species belongs is specified by symbols as indicated in the insets.

Panel A displays polyQ data for 48 species with Q_1/Q_2 ratio >4.5.

For the majority (n = 33, i.e. ~69 %) of these species, or related species belonging to the same genus, there is evidence in the literature of low-frequency/infrasonic vocalization/hearing, or their likelihood (see also the *Results* section; see [31], and [32], [33] for Cetartiodactyla and Perissodactyla, and see the *Results* and *Discussion* sections for Odontoceti; on Afrotheria species, see [34] for *Elephas maximus* (*Ele max*), [35] and [36] for *Chrysochloris asiatica* (*Chr asi*), [37] and [38] for *Elephantulus edwardii* (*Ele edw*), [39], [40], and [41] for the sirenians *Trichechus manatus* (*Tri Man*) and *Dugong dugon* (*Dug dug*), [42] for the Hyracoidea species *Procavia capensis* (*Pro cap*) and *Heterohyrax brucei* (*Het bru*); on Rodentia, see [17] and [43] for the taxon Sciuridae (which includes *Marmota marmota* (*Mar mar*) and *Marmota flaviventris* (*Mar fla*), and *Ictidomys tridecemlineatus* (*Ict tri*) in our dataset); on Primates, see [44] for the genus *Cercocebus* (which includes *Cercocebus atys* (*Cer aty*) in our dataset), and [45] for *Prolemus simus* (*Pro sim*); on Carnivora, see [46] for *Ailuropoda melanoleuca* (*Ail mel*); on Metatheria, see [47] for *Phascolarctos cinereus* (*Pha cin*). For other 7 species (i.e. ~15%), we could not find data in the literature on low frequency/infrasonic vocalization/hearing. For instance, little is known about vocalization/hearing frequency ranges for Pholidota and Xenarthra (e.g. [48]; 6 species in our dataset with Q_1/Q_2 ratio >4.5, including *Manis javanica* and *Manis pentadactyla*, which are not represented in the graph (Q_1+Q_2 = 44, Q_1/Q_2 = 10). At least some of them are fossorial [49], which may suggest low-frequency hearing sensitivity. The same applies to *Vombatus ursinus* (*Vom urs*), a large fossorial marsupial [50]. The remaining species with a Q_1/Q_2 ratio >4.5 (n = 8, i.e. ~16%) are apparent outliers, and include Odontoceti cetaceans (see on this the *Results* and *Discussion* sections), one single species from Eulipotyphla, one from Rodentia (which does not belong to Sciuroidea; see above), and two non-eutherian mammals. The two latter species, *Sorex araneus* (*Sor ara*; Eulipotyphla) and *Grammomys surdaster* (Rodentia) are known (*Sorex;* [51]) or presumed (*Grammomys*, based on what is known on other Muridae, see *Fig. S2B*) to emit USVs, thus representing outliers in terms of FOXP2 Q_1/Q_2 ratio. However, these species display at the same time convergence with USV-emitting bats and rodents in having a low (<0.5) FOXP1 Q_1/Q_2 ratio (see below), which is consistent with their ultrasonic vocalization frequency range. The other two non-eutherian mammals, i.e. *Monodelphis domestica* (*Mon dom*; Metatheria) and *Tachyglossus aculeatus* (*Tac acu*), do not have good low-frequency hearing ([52], [53]).

Panel B displays polyQ data for 88 species with Q_1/Q_2 ratio ≤3.5 (see *Results* and *Fig. 3C*).

For the majority (n = 83; i.e. ~94 %) of these species, or related species belonging to the same genus, there is evidence in the literature of ultrasonic vocalization/hearing, or its likelihood (on Chiroptera, see [54] and *Fig, S2A* for species name labels (omitted here for clarity); on Euarchontoglires (Primates, Dermoptera, Rodentia, and Lagomorpha), see *Fig. S2B,D*; on Carnivora, i.e. *Arctonyx collaris* (*Arc col*), *Panthera pardus* (*Pan par*), *Puma concolor* (*Pum con*), *Puma yagouaroundi* (*Pum yag*), *Acinonyx jubatus* (*Aci jub*), *Felis catus* (*Fel cat*), *Mustela putorius* (*Mus put*), *Suricata suricatta* (*Sur sur*), *Hyaena hyaena* (*Hya hya*), *Enhydra lutris* (*Enh lut*), *Lontra canadensis* (*Lon can*), *Lynx canadensis* (*Lyn can*), *Panthera leo* (*Pan leo*), *Neovison vison* (*Neo vis*), see [55], [56], and, specifically, [57] for *P. yagouaroundi*, [58] for *F. catus*, [59] on *M. putorius*, [60] for *E. lutris*, [61] and [62] for *N. vison*; on Cetartiodactyla, see [63] for *Vicugna pacos* (*Vic pac*); on Eulipotyphla, see [64] for *Erinaceus europaeus* (*Eri eur*); on Afrotheria, see [64] for *Microgale talazaci* (*Mic tal*). We could not find data in the literature on ultrasonic vocalization/hearing in 3 other species (i.e. ~4%), two from Xenarthra (i.e. *Myrmecophaga tridactyla* (*Myr tri*) and *Tamandua tetradactyla* (*Tam tet*)) and one from Afrotheria (i.e. *Orycteropus afer* (*Ory afe*)). *Camelus ferus* (Cetartiodactyla) and *Nannospalax galili* (Rodentia) two remaining

species with a Q_1/Q_2 ratio ≤3.5 (i.e. ~3%) are apparent outliers. However, species in the genus Camelus emit the highest frequency vocalizations in Artiodactyla [65], and the relative reduction of the Q_1/Q_2 ratio in *C. ferus* in comparison with other artiodactyl species may be related to this fact.

C-D. Details of regions of interest of the FOXP1 polyQ sum-*vs*-ratio plots for all the mammalian species that were analyzed. The (super)order to which each species belongs is specified by symbols as indicated in the insets.

Panel C displays the 5 species with FOXP1 Q_1/Q_2 ratio ≥2.

For the majority (n = 4; i.e. 80%) of these species, or closely related species, there is evidence in the literature of ultrasonic vocalization/hearing, or its likelihood (on Chiroptera, see [54] and *Fig, S2A* for *Noctilio leporinus* (*Noc lep*) and *Mormoops blainvillei* (*Mor bla*); on Lagomorpha), i.e. *Oryctolagus cuniculus* (*Ory cun*) and *Lepus europaeus* (*Lep eur*) see *Fig. S2B,D.* The only other species of this group, *Talpa occidentalis* (*Tal occ*; Eulipotyphla), a fossorial species, is an apparent outlier based on electrophysiological auditory responses limited to 16 kHz, as reported in [27].

Panel D displays the 46 species with a ratio ≤0.5 (see the *Results* section and *Fig. 2C*).

For the majority (n = 41; i.e. ~89 %) of these species, or related species belonging to the same genus, there is evidence in the literature of ultrasonic vocalization/hearing, or its likelihood (on Chiroptera, see [54] and *Fig, S2A, for Pteronotus parnellii* (*Pte par*), *Micronycteris hirsuta* (*Mic hir*), *Megaderma lyra* (*Meg lyr*), *Molossus molossus* (*Mol mol*); on Rodentia, see *Fig. S2B,D*, and [66] for calls extending in the ultrasonic range for a species in the genus *Fukomys* (represented by *Fukomys damarensis* (*Fuk dam*) in our dataset); on Eulipotyphla, see [67] for *Sorex araneus* (*Sor ara*), [64] for *Erinaceus europaeus* (*Eri eur*); on Afrotheria, see [68] and [64] for *Echinops telfairi* (*Ech tel*), [64] for *Microgale talazaci* (*Mic tal*); [69] and [42] for the Hyracoidea species *Procavia capensis* (*Pro cap*) and *Heterohyrax brucei* (*Het bru*); [70] for *Chrysochloris asiatica* (*Chr asi*); [71] and [72] for *Trichechus manatus* (*Tri man*); [41] for *Dugong dugon* (*Dug dug*).

For one species of Afrotheria, i.e. *Orycteropus afer* (*Ory afe*), we could not find data in the literature on high frequency/infrasonic vocalization/hearing (see [73]).

The remaining species with a Q_1/Q_2 ratio ≤0.5 (n = 4, i.e. ~9%) are apparent outliers. Two of these are *Elephas maximus* (*Ele max*) and *Loxodonta africana* (*Lox afr*), which emit and perceive infrasonic sound. However, both elephant species have a high FOXP2 Q_1/Q_2 ratio (≥ 4.5) converging with other species with low-frequency/infrasonic vocalization/hearing. This indicates that in these species, FOXP2 polyQ repeat lengths may be the main correlates of their vocalization/hearing frequency range. The same applies to one of the two other species, i.e. *Heterocephalus glaber* (*Het gla*), a subterranean rodent with degenerate high frequency hearing [74], which converges with non-USV emitting Chiroptera in terms of FOXP2 repeat lengths (Q1+Q2 >60, Q1/Q2 <3.5; see *Fig. 2C*). The last species of this group *Nannospalax galili* (*Nan gal*), another subterranean rodent with poor high-frequency hearing, is the only outlier in both FOXP2 (ratio ≤3.5) and FOXP1 (ratio ≤0.5). Notably, these latter four species belonging to Afrotheria and Rodentia are anyhow in a region of the FOXP1 polyQ graph occupied by other species from the same two taxa characterized by both both low- and high-frequency vocalization/hearing (see *Results* and *Fig. 4B-C* below).

Figure S4 – FOXP2/1 polyQ length variation and vocalization-related parameters in mammalian taxa (*related to Fig. 3*).

A. Scatterplots with regression lines (in *red*) highlighting significant correlations between mean FOXP2 Q_1/Q_2 ratio and mean minimum vocalization frequency (*left*; r = -0.89; n = 6 groups; p<0.02), or maximum vocalization frequency (*middle*; r = -0.83; n = 6 groups; p<0.05), or body mass (*right*; r = 0.97; n = 6 groups; p<0.001) in groups of mammalian species, excluding Chiroptera, binned by the same two parameters (see *Methods*). **B.** Bar graph plotting the mean log maximum vocalization frequency in groups of species, including Cetacea, with a FOXP2 Q1/Q2 ≤3.5 (*red*), between 3.5 and 4.5 (*gray*) and > 4.5 (*blue*). See *Fig. 3G, middle panel*, for the same graph not including Cetacea. Data are represented as mean +/- SEM. **C.** Bar graphs plotting the mean log minimum (*left*) and maximum (*middle*) vocalization frequencies, and the mean log body mass (*right*) in groups of species with a FOXP1 Q_1/Q_2 ≤0.2 (*red*) or 0.2-0.5 (*blue*). The two groups significantly differed for minimum vocalization frequency (p<0.01, t-test, n = 5-8 species per group) and body mass (p<0.01, t-test, n = 14-18 species per group). They also displayed a trend towards a significant difference in maximum vocalization frequency (p=0.07, *t*-test, n = 5-8 per group; but see *panel D*). Data are represented as mean +/- SEM. The species with vocalization data available that were included in the analysis are *Cavia porcellus, Dugong dugon, Elephas maximus, Loxodonta africana, Marmota flaviventris* (only minimum frequency), *Meriones unguiculatus, Mesocricetus auratus, Mus musculus, Procavia capensis, Rattus norvegicus, Rattus rattus, Trichechus manatus, Typhlomys cinereus.* The species with body mass data available that were included in the analysis are, besides those listed above for the frequency analyses, *Arvicanthis niloticus*, *Arvicola amphibius*, *Cavia porcellus*, *Chinchilla lanigera*, *Chrysochloris asiatica*, *Dipodomys ordii*, *Dugong dugon*, *Echinops telfairi*, *Elephas maximus*, *Heterocephalus glaber, Heterohyrax brucei*, *Jaculus jaculus*, *Loxodonta africana*, *Marmota flaviventris*, *Marmota marmota*, *Mastomys coucha*, *Meriones unguiculatus*, *Mesocricetus auratus*, *Microgale talazaci*, *Microtus ochrogaster*,

Microtus oregoni, *Mus caroli*, *Mus musculus*, *Octodon degus*, *Onychomys torridus*, *Orycteropus afer*, *Peromyscus leucopus*, *Peromyscus maniculatus*, *Procavia capensis*, *Rattus norvegicus*, *Rattus rattus*, *Trichechus manatus*. **D.** Both minimum and maximum vocalization frequencies significantly correlated with the Q_1/Q_2 ratio (r = -0.67, n = 13, p<0.02; r = -0.73, n = 12, p<0.001, respectively).

Figure S5 – FOXP2 polyQ length variation and propensity to form α-helical coiled coil (CC) structures (*related to Fig. 4*).

A. Prediction of the CC propensity profile for the full-length primary sequence of human FOXP2 obtained using Paircoil2 [4] and expressed as 1-*Pscore* [5]. Regions with high CC-propensity (0.8-1) are highlighted in black (peaks *cc1*-*cc4*). The gray bar on top represents the FOXP2 primary sequence with known structural and functional domains highlighted. The Q-rich region is in *pink* and its polyQ repeats (Q_1 and Q_2) are in *red*. The zinc finger (*Z*) is in *yellow*, the leucine zipper (*L*, which overlaps the cc4 peak) in *orange*, and the DNA-binding forkhead domain (*FHD*) in *light orange*. Note how the *cc2* and *cc3* peaks overlap the polyQ repeats Q_1 and Q_2, respectively. **B.** As in *panel A*, for FOXP2 orthologs of chiropteran species of interest (*Cyn sph*, *Min sch*, *Rhi fer*) in comparison with the human ortholog (*Hom sap*). Note how, while the extension of the *cc1* and *cc4* peaks (*asterisks*) is conserved, that of the polyQ bearing *cc2* and *cc3* peaks (highlighted by *black segments* on top) varies considerably across orthologs. **C.** Details of CC predictions as in *panel A*, highlighting the *cc2* and *cc3* peaks of FOXP2 orthologs in species of interest, i.e. the primates *Homo sapiens* (*Hom sap*) and *Carlito syrichta* (*Car syr*) and the bats *Cynopterus sphinx* (*Cyn sph*) and *Rhinolophus ferrumequinum* (*Rhi fer*). The position of glutamine (Q) and proline (P) residues is highlighted, respectively, by *red* or *blue* thin vertical bars above the Paircoil2 plot. Note how in both the USV-emitting species (i.e. *Car syr* and *Rhi fer*) multiple α-helix-breaking proline residues shorten the overall length of the two peaks and increase their degree of separation in comparison with their non-USV-emitting counterparts (i.e. *Hom sap* and *Cyn sph*) in the same taxa. Also see below *panel E*. **D.** Scatterplot illustrating the significant correlation between polyQ length (Q_1+Q_2) and predicted CC length (*cc2*+*cc3*) in a set of 16 FOXP2 orthologs from species belonging to the NE, FM and CF groups. CC length was determined by counting, in the *cc2* and *cc3* peaks, the number of residues with a propensity ≥0.8 (1-*Pscore*). **E.** Atomic-level structural predictions obtained with Raptor-X highlighting of the N-terminal region of the human FOXP2 (*Hom sap*; a.a. 1-244) and of the corresponding region, based on sequence alignment, of the *Cyn sph* (a.a. 1-264), *Min sch* (a.a. 1-232), and *Rhi fer* (a.a. 1-240) orthologs. Details of these predictions containing the Q_1-hinge-Q_2 section are shown in *Fig. 4C*. Glutamine residues are in *red*, proline residues in *blue* and Q-Q hydrogen bonds between Q_1 and Q_2 are in *yellow*.

Figure S6 – Atomic force microscopy (AFM) of FOXP2 polyQ peptides (*related to Fig. 5*).

A. Zenithal 3-D rendering of an AFM topography image of large assemblies formed the *Min sch*-Q_1Q_2 peptide. Note how fibrillary profiles are present on the surface of the larger assembly and appear to emerge from its external borders (*arrowheads*). These and other findings (see *Fig. 5*) support the notion that such large assemblies of this polyQ peptide may derive from tangling of fibrillary structures, as previously found for polyA CC peptides [75].

Figure S7 – PolyQ-related LLPS and transcriptional activity of FOXP2 (*related to Figs. 6-7*).

A. Bar graph illustrating the relative mean size of intracellular condensates formed by GFP-tagged full length FOXP2 or by its N-terminal CC domain (NTD_{CC}). Values are normalized to the mean size of FOXP2-GFP condensates. The condensates formed by the NTD_{CC} fragment are significantly larger than those formed by the full-length protein (1.73 ± 0.08, n = 2623, *vs* 1.00 ± 0.03, n = 5858, p<0.001, *t*-test). Data are represented as mean +/- SEM. **B.** Graph plotting the temporal kinetics of condensate formation and dissolution, quantified as the mean relative cell area occupied by condensates, up to 20 min after two distinct photoactivation pulses (*black arrowheads*) in cells expressing NTD_{CC}-mCh-Cry2. The data shown for the first LLPS induction are the same shown in *Fig. 6F*. Note how, when decayed after the first photoactivation pulse, LLPS can be readily reinduced in the same cells after a second pulse. Such reversibility and re-inducibility are hallmark features of LLPS [76]. Data are represented as mean +/- SEM. **C.** Control experiment for *Fig. 6H*. Confocal fluourescence microscopy images of the nucleus of a HEK293 cell co-expressing FOXP2-GFP and DsRed. Note in the overlay image (*merge*) how DsRed alone, unlike FOXP1-DsRed (Fig. 6H) is not preferentially recruited into FOXP2 condensates. Calibration bar: 10 µm. **D-E.** Scatterplots illustrating the close correlation between FOXP2 polyQ lengths and the transcriptional activity activity of the protein, as determined in the luciferase assay experiments shown in *Fig. 7H* (luminescence values normalized to a vector-only (no FOXP2) *control* group; see ANOVA analysis in the *Results* section for significance assessment). Note how transcriptional activity correlates with FOXP2 Q_1+Q_2 length sum (*panel D*) and, for variants from species with prevalent Q_1 length variation (as found

in most mammalian species), with FOXP2 Q_1/Q_2 ratio (*panel E*). The indicated polyQ variants of human (*Hs*) FOXP2 in both panels, i.e. the *Cs*, *Ms* and $\Delta Q_1 Q_2$ variants, are those indicated *in Fig. 7A*.

SUPPLEMENTAL REFERENCES

[1] Habersetzer, J., and Storch, G. (1992). Cochlea size in extant chiroptera and middle eocene microchiropterans from messel. Naturwissenschaften 79, 462–466. 10.1007/BF01139198.

[2] Simmons, N.B., Seymour, K.L., Habersetzer, J., and Gunnell, G.F. (2008). Primitive Early Eocene bat from Wyoming and the evolution of flight and echolocation. Nature 451, 818–821. 10.1038/nature06549.

[3] Ekdale, E.G. (2013). Comparative Anatomy of the Bony Labyrinth (Inner Ear) of Placental Mammals. PLoS ONE 8, e66624. 10.1371/journal.pone.0066624.

[4] McDonnell, A.V., Jiang, T., Keating, A.E., and Berger, B. (2006). Paircoil2: improved prediction of coiled coils from sequence. Bioinformatics 22, 356–358. 10.1093/bioinformatics/bti797.

[5] Fiumara, F., Fioriti, L., Kandel, E.R., and Hendrickson, W.A. (2010). Essential Role of Coiled Coils for Aggregation and Activity of Q/N-Rich Prions and PolyQ Proteins. Cell 143, 1121–1135. 10.1016/j.cell.2010.11.042.

[6] Ramsier, M.A., Cunningham, A.J., Moritz, G.L., Finneran, J.J., Williams, C.V., Ong, P.S., Gursky-Doyen, S.L., and Dominy, N.J. (2012). Primate communication in the pure ultrasound. Biol. Lett. 8, 508–511. 10.1098/rsbl.2011.1149.

[7] Bakker, J., van Nijnatten, T.J.M., Louwerse, A.L., Baarends, G., Arndt, S.S., and Langermans, J.A.M. (2014). Evaluation of ultrasonic vocalizations in common marmosets (Callithrix jacchus) as a potential indicator of welfare. Lab Anim 43, 313–320. 10.1038/laban.568.

[8] Cherry, J.A., Izard, M.K., and Simons, E.L. (1987). Description of ultrasonic vocalizations of the mouse lemur (Microcebus murinus) and the fat-tailed dwarf lemur (Cheirogaleus medius). Am. J. Primatol. 13, 181–185. 10.1002/ajp.1350130208.

[9] Miard, P., Lim, L.-S., Abdullah, N.I., Elias, N.A., and Ruppert, N. (2019). Ultrasound use by Sunda colugos offers new insights into the communication of these cryptic mammals. Bioacoustics 28, 397–403. 10.1080/09524622.2018.1463294.

[10] Dice, L.R., and Barto, E. (1952). Ability of Mice of the Genus Peromyscus to Hear Ultrasonic Sounds. Science 116, 110–111. 10.1126/science.116.3005.110.

[11] Pasch, B., Tokuda, I.T., and Riede, T. (2017). Grasshopper mice employ distinct vocal production mechanisms in different social contexts. Proc. R. Soc. B. 284, 20171158. 10.1098/rspb.2017.1158.

[12] Colvin, M.A. (1973). Analysis of Acoustic Structure and Function in Ultrasounds of Neonatal Microtus. Behav 44, 234–262. 10.1163/156853973X00418.

[13] Yurlova, D.D., Volodin, I.A., Ilchenko, O.G., and Volodina, E.V. (2020). Rapid development of mature vocal patterns of ultrasonic calls in a fast-growing rodent, the yellow steppe lemming (Eolagurus luteus). PLoS ONE 15, e0228892. 10.1371/journal.pone.0228892.

[14] Hashimoto, H., Saito, T.R., Moritani, N., Komeda, K., and Takahashi, K.W. (2001). Comparative Study on Isolation Calls Emitted from Hamster Pups. Exp. Anim. 50, 313–318. 10.1538/expanim.50.313.

[15] Hashimoto, H., Moritani, N., Aoki-Komori, S., Tanaka, M., and Saito, T.R. (2004). Comparison of Ultrasonic Vocalizations Emitted bv Rodent Pups. Exp. Anim. 53, 409–416. 10.1538/expanim.53.409.

[16] He, K., Liu, Q., Xu, D.-M., Qi, F.-Y., Bai, J., He, S.-W., Chen, P., Zhou, X., Cai, W.-Z., Chen, Z.-Z., et al. (2021). Echolocation in soft-furred tree mice. Science 372, eaay1513. 10.1126/science.aay1513.

[17] Heffner, R.S., and Heffner, H.E. (1992). Hearing and sound localization in blind mole rats (Spalax ehrenbergi). Hearing Research 62, 206–216. 10.1016/0378-5955(92)90188-S.

[18] Mason, M.J. (2016). Structure and function of the mammalian middle ear. I: Large middle ears in small desert mammals. J. Anat. 228, 284–299. 10.1111/joa.12313.

[19] Caspar, K.R., Heinrich, A., Mellinghaus, L., Gerhardt, P., and Begall, S. (2021). Evoked auditory potentials from African mole-rats and coruros reveal disparity in subterranean rodent hearing. Journal of Experimental Biology 224, jeb243371. 10.1242/jeb.243371.

[20] Heffner, R., Heffner, H., and Masterton, B. (1971). Behavioral Measurements of Absolute and Frequency-Difference Thresholds in Guinea Pig. The Journal of the Acoustical Society of America 49, 1888–1895. 10.1121/1.1912596.

[21] Martin, K., Tucker, M.A., and Rogers, T.L. (2017). Does size matter? Examining the drivers of mammalian vocalizations: EVOLUTION OF VOCALIZATION IN MAMMALS. Evolution 71, 249–260. 10.1111/evo.13128.

[22] Volodin, I.A., Volodina, E.V., Frey, R., Karaseva, K.D., and Kirilyuk, V.E. (2021). Daurian pika (Ochotona dauurica) alarm calls: individual acoustic variation in a lagomorph with audible through ultrasonic vocalizations. Journal of Mammalogy 102, 947–959. 10.1093/jmammal/gyab048.

[23] Heffner, H., and Masterton, B. (1980). Hearing in Glires: Domestic rabbit, cotton rat, feral house mouse, and kangaroo rat. The Journal of the Acoustical Society of America 68, 1584–1599. 10.1121/1.385213.

[24] Heffner, R.S., Koay, G., and Heffner, H.E. (2020). Hearing and sound localization in Cottontail rabbits, Sylvilagus floridanus. J Comp Physiol A 206, 543–552. 10.1007/s00359-020-01424-8.

[25] Nevo, E. (1990). Evolution of Nonvisual Communication and Photoperiodic Perception in Speciation and Adaptation of Blind Subterranean Mole Rats. Behav 114, 249–276. 10.1163/156853990X00158.

[26] Sales, G.D. (2010). Ultrasonic calls of wild and wild-type rodents. In Handbook of Behavioral Neuroscience (Elsevier), pp. 77–88. 10.1016/B978-0-12-374593-4.00009-7.

[27] Mason, M.J. (2016). Internally coupled ears in living mammals. Biol Cybern 110, 345–358. 10.1007/s00422-015-0675-1.

[28] New, E.M., Li, B.-Z., Lei, T.C., and McCullagh, E.A. (2021). Hearing Ability of Prairie Voles (Microtus ochrogaster) (Neuroscience) 10.1101/2021.10.07.463519.

[29] Matrosova, V.A., Schneiderová, I., Volodin, I.A., and Volodina, E.V. (2012). Species-specific and shared features in vocal repertoires of three Eurasian ground squirrels (genus Spermophilus). Acta Theriol 57, 65–78. 10.1007/s13364-011-0046-9.

[30] Wilson, D.R., and Hare, J.F. (2004). Ground squirrel uses ultrasonic alarms. Nature 430, 523–523. 10.1038/430523a.

[31] Mourlam, M.J., and Orliac, M.J. (2017). Infrasonic and Ultrasonic Hearing Evolved after the Emergence of Modern Whales. Current Biology 27, 1776-1781.e9. 10.1016/j.cub.2017.04.061.

[32] Heffner, R.S., and Heffner, H.E. (1983). Hearing in large mammals: Horses (Equus caballus) and cattle (Bos taurus). Behavioral Neuroscience 97, 299–309. 10.1037/0735-7044.97.2.299.

[33] Heffner, R.S., and Heffner, H.E. (1990). Hearing in domestic pigs (Sus scrofa) and goats (Capra hircus). Hearing Research 48, 231–240. 10.1016/0378-5955(90)90063-U.

[34] Payne, K.B., Langbauer, W.R., and Thomas, E.M. (1986). Infrasonic calls of the Asian elephant (Elephas maximus). Behav Ecol Sociobiol 18, 297–301. 10.1007/BF00300007.

[35] Willi, U.B., Bronner, G.N., and Narins, P.M. (2006). Middle ear dynamics in response to seismic stimuli in the Cape golden mole (Chrysochloris asiatica). Journal of Experimental Biology 209, 302–313. 10.1242/jeb.01989.

[36] Narins, P.M., Stoeger, A.S., and O'Connell-Rodwell, C. (2016). Infrasonic and Seismic Communication in the Vertebrates with Special Emphasis on the Afrotheria: An Update and Future Directions. In Vertebrate Sound Production and Acoustic Communication Springer Handbook of Auditory Research., R. A. Suthers, W. T. Fitch, R. R. Fay, and A. N. Popper, eds. (Springer International Publishing), pp. 191–227. 10.1007/978-3-319-27721-9_7.

[37] Faurie, A.S., Dempster, E.R., and Perrin, M.R. (1996). Footdrumming patterns of southern African elephant-shrews. Mammalia 60. 10.1515/mamm.1996.60.4.567.

[38] Randall, J.A. (2001). Evolution and Function of Drumming as Communication in Mammals. Am Zool 41, 1143–1156. 10.1093/icb/41.5.1143.

[39] Ketten, D.R., Odell, D.K., and Domning, D.P. (1992). Structure, Function, and Adaptation of the Manatee Ear. In Marine Mammal Sensory Systems, J. A. Thomas, R. A. Kastelein, and A. Ya. Supin, eds. (Springer US), pp. 77–95. 10.1007/978-1-4615-3406-8_4.

[40] Marsh, H., O'Shea, T.J., and Reynolds III, J.E. (2011). Ecology and Conservation of the Sirenia: Dugongs and Manatees 1st ed. (Cambridge University Press) 10.1017/CBO9781139013277.

[41] Zeh, D.R., Heupel, M.R., Limpus, C.J., Hamann, M., Fuentes, M.M.P.B., Babcock, R.C., Pillans, R.D., Townsend, K.A., and Marsh, H. (2015). Is acoustic tracking appropriate for air-breathing marine animals? Dugongs as a case study. Journal of Experimental Marine Biology and Ecology 464, 1–10. 10.1016/j.jembe.2014.11.013.

[42] Koren, L. (2006). Vocalization as an indicator of individual quality in the rock hyrax (Doctoral dissertation, Tel-Aviv University).

[43] Jackson, L.L., Heffner, H.E., and Heffner, R.S. (1997). Audiogram of the fox squirrel (Sciurus niger). Journal of Comparative Psychology 111, 100–104. 10.1037/0735-7036.111.1.100.

[44] Brown, C. H. (1986). The perception of vocal signals by blue monkeys and grey-cheeked mangabeys. Experimental Biology 45, 145-165.

[45] Bergey, C., and Patel, E.R. (2008). A preliminary vocal repertoire of the greater bamboo lemur (Prolemur simus). Nexus 1, 69-84.

[46] Owen, M.A., Keating, J.L., Denes, S.L., Hawk, K., Boroski, J., Fiore, A., and Swaisgood, R.R. (2011). Behavioral audiogram of the giant panda (Ailuropoda melanoleuca): Preliminary results. The Journal of the Acoustical Society of America 130, 2460–2460. 10.1121/1.3654882.

[47] Frey, R., Reby, D., Fritsch, G., and Charlton, B.D. (2018). The remarkable vocal anatomy of the koala (Phascolarctos cinereus): insights into low-frequency sound production in a marsupial species. J. Anat. 232, 575–595. 10.1111/joa.12770.

[48] DiPaola, J.D., Yindee, M., and Plotnik, J.M. (2020). Investigating the use of sensory information to detect and track prey by the Sunda pangolin (Manis javanica) with conservation in mind. Sci Rep 10, 9787. 10.1038/s41598-020-65898-x.

[49] Ingram, D.J., Willcox, D., and Challender, D.W.S. (2019). Evaluation of the application of methods used to detect and monitor selected mammalian taxa to pangolin monitoring. Global Ecology and Conservation 18, e00632. 10.1016/j.gecco.2019.e00632.

[50] Guy, T.R., and Kirkpatrick, J.B. (2021). Environmental associations and effects of disturbances by common wombats in alpine Tasmania. Austral Ecology 46, 1392–1403. 10.1111/aec.13093.

[51] Thomas, J. A., and Jalili, M. S. (2004). Echolocation in insectivores and rodents. In: Echolocation in bats and dolphins (University of Chicago Press), pp. 547-564.

[52] Coleman, M.N., and Boyer, D.M. (2012). Inner Ear Evolution in Primates Through the Cenozoic: Implications for the Evolution of Hearing. Anat Rec 295, 615–631. 10.1002/ar.22422.

[53] Grothe, B., and Pecka, M. (2014). The natural history of sound localization in mammals â€“ a story of neuronal inhibition. Front. Neural Circuits. 8. 10.3389/fncir.2014.00116.

[54] Collen, A.L. (2012). The evolution of echolocation in bats: a comparative approach (Doctoral dissertation, University College London).

[55] Kitchener, A. C., Van Valkenburgh, B., Yamaguchi, N., Macdonald, D., and Loveridge, A. (2010). Felid form and function. Biology and conservation of wild felids 2010,83-106.

[56] Kruger, M.C. (2021). Ultrasonic Hearing in Cats and Other Terrestrial Mammals. Acoust. Today 17, 18. 10.1121/AT.2021.17.1.18.

[57] Ewer, R. F. (1998). The carnivores (Cornell University Press).

[58] Heffner, R.S., and Heffner, H.E. (1985). Hearing in Mammals: The Least Weasel. Journal of Mammalogy 66, 745–755. 10.2307/1380801.

[59] Kelly, J.B., Kavanagh, G.L., and Dalton, J.C.H. (1986). Hearing in the ferret (Mustela putorius): Thresholds for pure tone detection. Hearing Research 24, 269–275. 10.1016/0378-5955(86)90025-0.

[60] Ghoul, A., and Reichmuth, C. (2014). Hearing in the sea otter (Enhydra lutris): auditory profiles for an amphibious marine carnivore. J Comp Physiol A 200, 967–981. 10.1007/s00359-014-0943-x.

[61] Tubbert Clausen, K., Malmkvist, J., and Surlykke, A. (2008). Ultrasonic vocalisations of kits during maternal kit-retrieval in farmed mink, Mustela vison. Applied Animal Behaviour Science 114, 582–592. 10.1016/j.applanim.2008.03.008.

[62] Brandt, C., Malmkvist, J., Nielsen, R.L., Brande-Lavridsen, N., and Surlykke, A. (2013). Development of vocalization and hearing in American mink (Neovison vison). Journal of Experimental Biology, jeb.080226. 10.1242/jeb.080226.

[63] Heffner, R.S., Koay, G., and Heffner, H.E. (2014). Hearing in alpacas (Vicugna pacos): Audiogram, localization acuity, and use of binaural locus cues. The Journal of the Acoustical Society of America 135, 778–788. 10.1121/1.4861344.

[64] Poduschka, W. (1977). Insectivore communication. In: How animals communicate, T.A. Sebeok, ed. (Indiana University Press), pp. 600-633.

[65] Volodin, I.A., Volodina, E.V., and Rutovskaya, M.V. (2022). Camel whistling vocalisations: male and female call structure and context in Camelus bactrianus and Camelus dromedarius. Bioacoustics 31, 132–147. 10.1080/09524622.2021.1889403.

[66] Bednářová, R., Hrouzková-Knotková, E., Burda, H., Sedláček, F., and Šumbera, R. (2013). Vocalizations of the giant mole-rat (Fukomys mechowii), a subterranean rodent with the richest vocal repertoire. Bioacoustics 22, 87–107. 10.1080/09524622.2012.712749.

[67] Forsman, K.A., and Malmquist, M.G. (1988). Evidence for echolocation in the common shrew, Sorex araneus. Journal of Zoology 216, 655–662. 10.1111/j.1469-7998.1988.tb02463.x.

[68] Drexl, M., Faulstich, M., von Stebut, B., Radtke–Schuller, S., and Kössl, M. (2003). Distortion Product Otoacoustic Emissions and Auditory Evoked Potentials in the Hedgehog Tenrec, Echinops telfairi. JARO 4, 555–564. 10.1007/s10162-002-3043-5.

[69] Frydman, G., Goll, Y., Geffen, E., and Koren, L. (2023). Sex differences in frequencies in a species with modest sexual size dimorphism. Bioacoustics 32, 230–240. 10.1080/09524622.2022.2105954.

[70] Willi, U.B., Bronner, G.N., and Narins, P.M. (2006). Ossicular differentiation of airborne and seismic stimuli in the Cape golden mole (Chrysochloris asiatica). J Comp Physiol A 192, 267–277. 10.1007/s00359-005-0070-9.

[71] Ramos, E.A., Maust-Mohl, M., Collom, K.A., Brady, B., Gerstein, E.R., Magnasco, M.O., and Reiss, D. (2020). The Antillean manatee produces broadband vocalizations with ultrasonic frequencies. The Journal of the Acoustical Society of America 147, EL80–EL86. 10.1121/10.0000602.

[72] Gerstein, E.R., Gerstein, L., Forsythe, S.E., and Blue, J.E. (1999). The underwater audiogram of the West Indian manatee (Trichechus manatus). The Journal of the Acoustical Society of America 105, 3575–3583. 10.1121/1.424681.

[73] Berlioz, E., Cornette, R., Lenoir, N., Santin, M.D., and Lehmann, T. (2021). Exploring the ontogenetic development of the inner ear in Aardvarks. Journal of Anatomy 238, 1128–1142. 10.1111/joa.13361.

[74] Heffner, R.S., and Heffner, H.E. (1993). Degenerate hearing and sound localization in naked mole rats (Heterocephalus glaber), with an overview of central auditory structures. J. Comp. Neurol. 331, 418–433. 10.1002/cne.903310311.

[75] Pelassa, I., Corà, D., Cesano, F., Monje, F.J., Montarolo, P.G., and Fiumara, F. (2014). Association of polyalanine and polyglutamine coiled coils mediates expansion disease-related protein aggregation and dysfunction. Human Molecular Genetics 23, 3402–3420. 10.1093/hmg/ddu049.

[76] Shin, Y., Berry, J., Pannucci, N., Haataja, M.P., Toettcher, J.E., and Brangwynne, C.P. (2017). Spatiotemporal Control of Intracellular Phase Transitions Using Light-Activated optoDroplets. Cell 168, 159-171.e14. 10.1016/j.cell.2016.11.054.
